# Elucidating binding hot spots and structural stability in sirtuin family proteins for selective inhibitors: a computational approach

**DOI:** 10.3389/fbinf.2026.1786061

**Published:** 2026-04-10

**Authors:** Deepak Sharma, Rajiniraja Muniyan

**Affiliations:** School of Bio-Sciences and Technology, Vellore Institute of Technology, Vellore, Tamil Nadu, India

**Keywords:** conformation dynamics, key residue identification, molecular docking, molecular dynamics, sirtuin

## Abstract

**Background:**

The sirtuin (SIRT) family is an NAD^+^-dependent class III histone deacetylase protein that comprises seven members (SIRT1–7). SIRTs are involved in many cellular pathways, which enable them to act as significant regulators of critical diseases such as cancer, cardiovascular disease, respiratory disease, and diabetes. Despite extensive research conducted to understand SIRT biology, many areas remain unexplored, such as the lack of SIRT isoform selectivity and specificity, restrained potency, limited bioavailability, poor pharmacokinetic and pharmacodynamic properties, and insufficient clinical and preclinical trials. Our study focused on one of the major research gaps, i.e., “lack of SIRT isoform selectivity and specificity,” through extensive computational exploration.

**Methods:**

The workflow of our study included molecular docking and molecular dynamics studies to deeply explore conformational dynamics and binding hot spots selective to each SIRT isoform.

**Results:**

As an outcome of our study, we predicted the major flexible regions in each isoform, which may turn out to be selective for each SIRT isoform. Additionally, we predicted SIRT isoform-selective key residues that may regulate the inhibitory potential of SIRT proteins. The primary SIRT isoform-selective residues for SIRT1 were Phe273, Phe297, Tyr280, and His363; for SIRT2, they were Phe119, His187, Val233, Phe235, and Leu239; for SIRT3, they were Leu248, Glu296, and Arg301; for SIRT5, they were Phe70, Tyr102, Gln140, and His158; and for SIRT6, they were Asp61, Trp69, His131, Trp186, Ser214, Arg218, and Leu239.

**Conclusion:**

In this study, we provided a deep cognizance of SIRT biology and a fruitful initiative for *in vitro* exploration of SIRT-selective inhibitors and an *in silico* contribution toward their clinical trial success.

## Graphical Abstract

**Figure:**
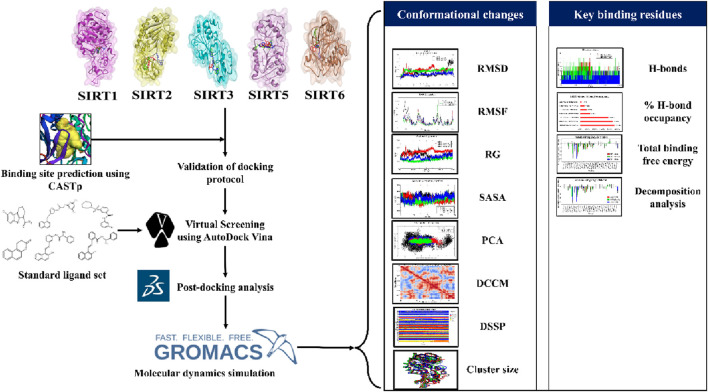

## 1 Background

Sirtuins (SIRTs) are the NAD^+^-dependent class III histone deacetylase proteins. SIRTs play a significant role in the deacetylation of various histone and non-histone proteins, such as p53, FOXO, Ku70, p300, Rb, E2F1, NF-kB, p3, PGC-1, H1, H3, and H4 (Yi and Luo, 2010). The mammalian SIRT family, consisting of seven members, is subclassified into four categories: class I (SIRT1, SIRT2, and SIRT3), class II (SIRT4), class III (SIRT5), and class IV (SIRT6 and SIRT7), based on molecular phylogenetic studies (Frye, 2000; Finkel et al., 2009). SIRTs are also classified based on their subcellular location. SIRT1, SIRT6, and SIRT7 are mainly found in the nucleus, SIRT2 in the cytoplasm, and SIRT3, SIRT4, and SIRT5 in the mitochondria. A fraction of SIRT1 may also be found in the cytoplasm and SIRT2 in the nucleus under certain conditions of cell types and cell cycle stages (Dang, 2014; Wu et al., 2022). The structure of an SIRT protein is composed of a small zinc-binding domain and a large Rossmann-fold domain. The groove that separates the aforementioned domain is the binding site for the acylated substrate and the NAD^+^ cofactor (Fiorentino et al., 2022). Specifically, an NAD^+^-binding region can be further differentiated into the adenine–ribose binding site (pocket A), the nicotinamide–ribose binding site (pocket B), and the nicotinamide moiety binding site (pocket C) (Yuan and Marmorstein, 2012). Steps such as NAD^+^ and acetyl group binding, cleavage of the glycosidic bond, acetyl group transfer, and formation of 2′-O-acetyl-ADP-ribose (2, O-acetyl-ADPR), NAM, and deacetylated lysine product take place during the mechanistic deacetylation by the SIRT family (Zhao et al., 2004). The SIRT family of proteins plays a significant role in many cellular biological events, such as inflammation, metabolism, oxidative stress, and apoptosis. Therefore, the SIRT family is considered a potential target in the pathophysiology of various systems and diseases, such as cancer, cardiovascular disease, respiratory disease, digestive system, nervous system, genitourinary system, diabetes, and others (Wu et al., 2022).

Among all the isoforms, SIRT1 is the most studied (with more than 11,000 articles indexed in PubMed), followed by SIRT2 and SIRT3 (combined, 3,700 articles). Other SIRTs are ignored or have not been much explored (Sinha et al., 2019; Curry et al., 2021). There is a need to understand more about the significance of all SIRT isoforms. The scope of this study is limited to the selective inhibition of SIRT isoforms. Many studies have been conducted to understand the inhibition of the human SIRT protein. For example, the upregulation of SIRT1 has been found to increase the chances of prostate and thyroid tumors by downregulating the tumor suppressor PTEN (phosphatase and tensin homolog) (Herranz et al., 2013). SIRT1 plays an influential role in enhancing drug resistance in chronic myeloid leukemia (CML) cells (Wang et al., 2013). In another study, the presence of SIRT1 worsened cardiac hypertrophy by encouraging membrane localization and upregulation of AKT and phosphoinositide-dependent protein kinase 1 (Sundaresan et al., 2011). Another example related to Huntington’s disease involves the inhibition of SIRT1 using an indole-based compound, i.e., EX-527. It was identified as safe and well tolerated by patients with Huntington’s disease. EX-527 also entered clinical trials, but it did not provide any significant benefits over the placebo control (Süssmuth et al., 2015). This long list of SIRT-inhibitory potential across different diseases suggests that inhibition of SIRT might be a novel approach to reduce drug resistance and provide a novel treatment option for different diseases when used alone or in conjunction with proven treatments. However, controversies regarding SIRT inhibition and activation across different diseases remain a major challenge. Despite significant research conducted to understand SIRT biology and its connection to different diseases, there remain many unexplored research gaps, such as a lack of SIRT-isoform selectivity and specificity, moderate potency, limited bioavailability, poor pharmacokinetic and pharmacodynamic properties, inadequate clinical and preclinical trials that have been conducted to identify SIRT modulators, allosteric modulation of the SIRT protein, and a deeper understanding regarding the role of SIRTs in disease progression (Curry et al., 2021; Sharma et al., 2025).

Computer-aided drug design (CADD) is a recently evolving trend that incorporates computational techniques such as molecular docking, molecular dynamics simulation (MDS), artificial intelligence (AI), and machine learning (ML) (Vemula et al., 2023; Jain et al., 2025). In this study, we emphasize “lack of SIRT isoform selectivity and specificity” by studying the conformational changes in SIRT isoforms with and without standard ligand binding. In addition, we focus on identifying key residues present in the binding pocket of each SIRT isoform. The workflow of this study involves the retrieval of SIRT-selective and -non-selective standard compounds and experimentally determined crystallographic structures of different SIRT isoforms, followed by their preparation. Molecular docking and longer MDS studies were conducted in subsequent steps. After MDS, analysis of all trajectories was also performed to address the identified research gap. For conformational change upon binding of the ligand, we have conducted root mean square deviation (RMSD), root mean square fluctuation (RMSF), radius of gyration (RG), solvent-accessible surface area (SASA), cluster analysis, principal component analysis (PCA), dynamic cross-correlation matrix (DCCM), and define secondary structure of proteins (DSSP), whereas for the identification of key residues for each isoform, analyses such as H-bond, H-bond percentage occupancy, and binding free energy calculation with special emphasis on the per-residue energy contributions have been conducted. This study aids in understanding the effect of conformational changes in SIRT isoforms (structural biology), structure-based drug design (SBDD), and ligand optimization to increase specificity and reduce off-target effects.

## 2 Materials and methods

### 2.1 Selection and retrieval of SIRT proteins

The PDB IDs of SIRT1, SIRT2, SIRT3, SIRT5, and SIRT6 proteins were selected via a thorough literature review (Sinha et al., 2019; Sharma et al., 2022), and their crystallographic structures were retrieved from the RCSB PDB database (https://www.rcsb.org/). Among the seven isoforms of SIRT, SIRT4 and SIRT7 were not considered in the study due to the unavailability of their exact experimentally determined 3D structures. The parameters, such as lower resolution, *Homo sapiens* as the organism, the gold standard X-ray crystallographic experimental method, and the absence of mutations, were considered while selecting the PDB IDs. The selected PDB IDs were as follows: SIRT1 (PDB ID: 4ZZI), SIRT2 (PDB ID: 1J8F), SIRT3 (PDB ID: 5D7N), SIRT5 (PDB ID: 2NYR), and SIRT6 (PDB ID: 3K35) (Finnin et al., 2001; Schuetz et al., 2007; Pan et al., 2011; Dai et al., 2015; Rumpf et al., 2015). A detailed description of each selected PDB ID, including its resolution, and experimental method, is provided in Supplementary Table S1. These retrieved protein structures were carried forward for further analysis.

### 2.2 Selection, retrieval, and preparation of the standard ligand dataset

There were many SIRT-isoform-selective and -non-selective inhibitors mentioned in the literature (Sinha et al., 2020; Sharma et al., 2025). The aim of this study was to identify the key residues involved in the inhibitory activity of each isoform. Therefore, we selected a few compounds with effective for SIRT-selective and -non-selective inhibitory activity. The selected compounds were retrieved from a fruitful resource in PubChem (https://pubchem.ncbi.nlm.nih.gov/) in SDF format. The detailed selected compound list, their PubChem ID, and chemical structures are briefly mentioned in Supplementary Table S2. The selected compounds were sirtinol, EX527 (selisistat), splitomicin, tenovin-1, tenovin-6, AGK2, salermide, HR-73, SirReal2, cambinol, aristoforin, nicotinamide, Inauhzin, AK-7, and AC93253 (Mai et al., 2005; Heltweg et al., 2006; Gey et al., 2007; Zhang et al., 2009; 2012; Pasco et al., 2010; McCarthy et al., 2012; Rotili et al., 2012; Suzuki et al., 2012; Yuan et al., 2012; Lancelot et al., 2013; Broussy et al., 2020). Furthermore, these ligands were prepared and minimized using the MMFF94 force field for 1000 steps with the OpenBabel 3.3.1 tool (O’Boyle et al., 2011). The final prepared ligand structures were saved in PDBQT format.

### 2.3 Binding site prediction and self-docking

Each SIRT isoform has a large Rossmann fold and a small zinc-binding domain. However, a few changes have been noticed in the catalytic binding site of each domain, leading to a similar catalytic mechanism but different sequences in each SIRT isoform (Fiorentino et al., 2022). Therefore, binding site detection was the most critical step in this study. As part of our observations, some of the selected PDB structures contained a co-crystallized ligand in the experimentally verified binding site, whereas other did not. To maintain the uniformity of the study, we chose a well-proven web tool, i.e., the Computed Atlas of Surface Topography of proteins (CASTp 3.0) (Tian et al., 2018). Recent theoretical and algorithmic findings in computational geometry serve as the foundation for this CASTp 3.0 web tool. The SIRT1 protein structures exhibited an embedded co-crystallized ligand (4-(4-{2-[(methylsulfonyl)amino]ethyl}piperidin-1-yl)thieno[3,2-d]pyrimidine-6-carboxamide (1NS)) as an inhibitor molecule of the SIRT1 protein. We performed self-docking to validate the docking protocol with the 1NS molecule in the catalytic pocket of the SIRT1 protein. Unfortunately, we could not perform self-docking for all SIRT1 isoforms due to the unavailability of inhibitor molecules for the other isoforms. This step helped authenticate the protocol followed for virtual screening. Furthermore, the selected key residues in the binding of each SIRT isoform were cross-checked through the literature.

### 2.4 Virtual screening using AutoDock Vina

Rigid protein and flexible ligand-based molecular docking were considered in this study. To do so, each protein structure was prepared in AutoDock 4.2 (Morris et al., 2009). As part of the protein preparation steps, water molecules, additional side chains, and any heteroatoms were removed from the protein structure, whereas polar hydrogen and Kollman charges were added to the protein structure. After these steps, charges were spread to charge-deficient atoms, and each prepared protein was saved in PDBQT format. This created a uniform apo-like starting state across isoforms. A site-specific grid box was constructed around the predicted key residues, with its center coordinates (center x × center y × center z) and grid dimensions (size x × size y × size z) detailed in Supplementary Table S1. Furthermore, these site-specific grid boxes were used to perform virtual screening of selected standard-known compounds using the AutoDock Vina tool (Jaghoori et al., 2016). As the outcome of this step, we generated a .pdbqt_log file, and out.pdbqt files were further evaluated in post-docking analysis.

### 2.5 Post-docking analysis

All ligand molecules docked against SIRT1, SIRT2, SIRT3, SIRT5, and SIRT6 proteins were deeply investigated in terms of docking score, number of hydrogen bonds (H-bonds), other types of bonds, and size of binding pocket using the Discovery Studio Visualizer (DSV) (https://www.3ds.com/products/biovia/discovery-studio). In this study, we identified the common compounds that exhibited the most prominent effects across all five SIRT isoforms to maintain uniformity for comparison in subsequent steps. Such compounds were further considered for Molecular dynamics simulation (MDS) studies.

### 2.6 Molecular dynamics simulation studies

MDS studies for the apoprotein and the selected protein–ligand complexes were conducted using the GROMACS 23.1 version (Abraham et al., 2015). The ligand topology, for each docked complex, was calculated using the SwissParam web tool (Zoete et al., 2011), whereas the protein topology was generated using the “pdb2gmx” GROMACS utility by placing the protein in a triclinic box of 1 nm dimension, along with TIP3P water molecules and the CHARMM27 force field. The prepared topology of the respective protein and ligand was manually merged to form the protein–ligand complex, whereas this step was excluded in the apoprotein. All the systems were neutralized by sodium (Na^+^) and chlorine (Cl^−^) counter ions, followed by the execution of solvation, minimization, and equilibration of the system. The steepest descent algorithm was used in the energy minimization step for 50,000 steps, which is the first stage of MDS. In subsequent steps, the protein–ligand systems were subjected to two stages of equilibration, each for 100 ps. In the first equilibration, the NVT ensemble was conducted using the Berendsen protocol, whereas in the second equilibration, the NPT ensemble using the Parrinello–Rahman barostat (1 bar pressure and 300 K temperature) approach was followed. Final MD production of respective apoprotein and protein–ligand complexes was executed for 200 ns, followed by trajectory analysis such as RMSD, RMSF, RG, and SASA.

To assess the global motion of C-alpha (C-α) atoms of the backbone, PCA studies were conducted using gmx_covar GROMACS utilities for all SIRT apo and holo trajectories. Additionally, to identify the correlated and anti-correlated residues throughout the simulation period, we have also executed DCCM analysis. It enabled us to calculate the percentage of correlated, anti-correlated, and neutral residues among different trajectories. Furthermore, to confirm the conformational changes in the protein’s secondary structure elements, we conducted DSSP analysis. Within DSSP, two different analyses, secondary structure timeline plot (SSTP) and change in the total number of structured residues (NSRs), were performed using GROMACS and Python scripts. To gain deeper insight into the conformational changes in the protein in the apo and holo states, we conducted cluster analysis using the gmx_cluster package in GROMACS. This analysis allowed us to identify the number of clusters sampled by each trajectory, along with their representative structures. We have also superimposed these representative structures to notice the considerable change in the C-alpha atoms of each SIRT isoform.

The second part of the study involved identifying the binding hot spot of each SIRT isoform. To do so, we conducted H-bond analysis and calculated the percentage of H-bond occupancy throughout the simulation period using VMD software (Humphrey et al., 1996). This approach provided the dual advantage of assessing the stability of the SIRT–top3 ligand complex and identifying the key residues involved in H-bond formation in the dynamic state. Furthermore, total binding free energy (TBFE) or molecular mechanics Poisson–Boltzmann surface area (MMPBSA) of all protein–ligand systems was calculated using the gmx_MMPBSA package (Valdés-Tresanco et al., 2021). This package utilized MMPBSA.py with AMBER to calculate the binding free energy using GROMACS-generated files (Miller et al., 2012). The final 20 ns (2,000 frames) of the MDS trajectory were selected to compute the binding free energy. This maintained the uniformity in all the protein–ligand systems for the calculation of TBFE. A decomposition analysis was also performed to identify the per-residue energy contribution in each SIRT–top3 ligand complex, providing a comprehensive basis for selecting SIRT-isoform-selective key residues involved in the inhibitory activity of the SIRT protein.

The plots generated in the study were visualized using the XMgrace (https://plasma-gate.weizmann.ac.il/Grace/) tool in a Linux environment. The interpretation of most of the plots and the methodology followed have already been described in our previous studies (Karunakaran and Muniyan, 2023a; 2023b; Sharma and Muniyan, 2024; Bell I and Muniyan, 2025; Jena et al., 2025). Various Python packages, such as MDA analysis (Michaud-Agrawal et al., 2011; Gowers et al., 2016), Matplotlib (Hunter, 2007), seaborn (Waskom, 2021), Pandas (McKinney, 2010), and NumPy (Harris et al., 2020), were used for plotting different data in this study. The complete scripts, along with the corresponding analyses, are provided in our GitHub Repository.

## 3 Results

### 3.1 Binding site prediction and self-docking

To address the most critical step of binding site prediction, we used the CASTp 3.0 tool. This way, we could treat all the protein–ligand complexes uniformly. Additionally, self-docking for the SIRT1 protein with 1NS as the co-crystallized inhibitor was performed to validate the docking protocols. A calculated RMSD value of 2.339 Å after superimposition of the docked complex and co-crystallized ligand revealed the authenticity of the docking protocol of our study (Supplementary Figure S1). Although values below 2.0 Å are ideal, RMSD values up to ∼2.5 Å are commonly considered reliable.

### 3.2 Virtual screening

All SIRT PDB structures were prepared using the AutoDock tool to conduct a molecular docking study, as described earlier. Once the site-specific grid box was constructed for each protein, the standard ligand dataset was docked on SIRT1, SIRT2, SIRT3, SIRT5, and SIRT6 proteins using the AutoDock Vina tool. The advantage of using AutoDock Vina is the easy and efficient screening algorithm. All the protein–ligand complexes (15 ligands and 5 proteins, i.e., 75 complexes) were further subjected to the post-docking analysis step.

### 3.3 Post-docking analysis

Post-docking analysis is the most critical step. The docking scores in the virtual screening step ranged from −4.1 kcal/mol to −11.6 kcal/mol. Supplementary Table S3 describes the complete docking score details, types of interactions, number of H-bonds, and size of the binding pocket of all 15 standard compounds against SIRT1, SIRT2, SIRT3, SIRT5, and SIRT6 proteins. In the subsequent section, we discuss the three major predicted inhibitor molecules for each isoform and the key residues found to interact with the ligands via various types of bonds. Since the study aimed to identify isoform-selective key residues, we developed an in-house Python script to determine the occurrence of key residues in at least 50% of the compounds against each SIRT isoform. The outcome of the occurrence count of the key residues was provided in each case with annotation as residue number (occurrence in number of compounds). For example, His363 (13) indicates that His363 interaction was observed in 13 compounds out of 15 compounds. The same script is provided in our GitHub repository.

#### 3.3.1 SIRT1 and standard ligand dataset analysis

Overall docking scores of standard compounds against the SIRT1 protein ranged from −5.1 kcal/mol to −11.4 kcal/mol. Among all 15 compounds, the top three were salermide, Inauhzin, and sirtinol, with the docking scores of −11.6 kcal/mol, −10.8 kcal/mol, and −11.1 kcal/mol, respectively. The bond formation description against the SIRT1 protein for salermide was as follows: H-bond: His363 and Val412; C–H bond: His363; van der Waals interactions (VWs): Ile270, Asp272, Arg274, Gln345, Asn346, Ile411, Phe414, and Leu418; and Pi bond: Ala262, Phe273, Phe297, Ile347, His363, and VAl445; that for Inauhzin was as follows: H-bond: Gln345; VW: Lys203, Ile270, Pro271, Asp272, Arg274, Phe297, Asn346, Asp348, His363, and Phe414; Pi bond: Ala262, Phe273, Ile347, and Val445; and other bonds: Tyr280; that for sirtinol was as follows: H-bond: Tyr280; C–H bond: His363; VW: Ile270, Asp272, Arg274, Gln345, Asn346, Ile411, Val412, and Phe414; and Pi bond: Ala262, Phe273, Phe297, Ile347, His363, and Val445.

The occurrence counts of the key residues in at least 50% of the compounds against the SIRT1 protein were as follows: His363 (13), Phe273 (12), Phe414 (12), Ile347 (12), Gln345 (11), Phe297 (11), Val445 (10), Val412 (10), Ala262 (9), Asn346 (8), Tyr280 (7), Ile270 (7), Phe413 (7), Arg446 (7), and Leu418 (7).

#### 3.3.2 SIRT2 and standard ligand dataset analysis

The docking score for the SIRT2 protein ranged from −4.6 kcal/mol to −9.7 kcal/mol. The top three compounds against the SIRT2 protein were salermide, HR-73, and sirtinol, with their docking scores of −9.3 kcal/mol, −9.3 kcal/mol, and −9.7 kcal/mol, respectively. The interactions formed by salermide were as follows: VW: Pro99, Glu108, and Ile169; Pi bond: Phe96, Arg97, Tyr104, Leu107, Leu112, Ile118, Phe119, Leu134, and Leu138; those formed by HR-73 were as follows: VW: Pro115 and Leu134; Pi bond and alkyl bond: Phe96, Tyr104, Leu107, Leu112, Ile118, Phe119, Leu138, and Ile169; and those formed by sirtinol were as follows: C–H bond: Tyr104; VW: Leu107. Leu112, Pro115, Leu134, Ile169, Ile232, Val233, Phe234, and Phe235; Pi bond: Phe96, Tyr104, Ile118, Phe119, Leu138, and His187.

The occurrence counts of the key residues in at least 50% of the compounds against the SIRT2 protein were as follows: Phe96 (10), Phe119 (9), Ile169 (9), Leu107 (8), Ile118 (8), Tyr104 (8), Leu112 (8), Leu134 (8), Leu138 (7), and Pro115 (7).

#### 3.3.3 SIRT3 and standard ligand dataset analysis

For the SIRT3 protein, the docking scores ranged from −4.7 kcal/mol to −10 kcal/mol. The top three ligands against SIRT3 were AGK2, SirReal2, and salermide, with docking scores of −8.9 kcal/mol, −9.1 kcal/mol, and −10 kcal/mol, respectively. The details regarding the formation of bonds for AGK2 were as follows: C–H bond: Leu211 and Thr255; VW: Arg235, Val245, Thr250, Ser253, Thr257, Asp290, Ile291, Pro297, Pro299, and Gln300; Pi bond and alkyl bond: Glu246, Lys288, Val292, Glu296, and Arg301; those for SirReal2 were as follows: VW: Glu246, Gly249, Thr250, Thr255, Thr257, Asp290, Val292, Pro297, and Gln300; Pi bond and alkyl bond: Leu182, Phe186, Lys288, Glu296, Pro299, and Arg301; those for salermide were as follows: H-bond: Lys288 and Pro289; VW: Arg235, Leu244, Val245, Glu246, Gly249, Thr250, Thr255, Thr257, Gln260, Ile291, and Gln300; Pi bond and alkyl bond: Lys288, Asp290, Val292, Glu296, Pro299, and Arg301.

The occurrence counts of the key residues in at least 50% of the compounds against the SIRT3 protein were as follows: Glu296 (11), Val292 (10), Pro299 (10), Thr255 (10), Asp290 (10), Lys288 (9), Thr250 (8), Arg301 (7), Glu246 (7), Gly249 (7), and Thr257 (7).

#### 3.3.4 SIRT5 and standard ligand dataset analysis

Similarly, the docking score for SIRT5 protein was in the range of −4.1 kcal/mol to −9.7 kcal/mol, and the top three complexes were SirReal2, salermide, and Inauhzin, with docking scores of −9.6 kcal/mol, −9.7 kcal/mol, and −9 kcal/mol, respectively. The bond formations occurred for SirReal2 were as follows: H-bond: Arg105; VW: Ala59, Thr69, Tyr102, Gln140, Asn141, Val220, Val221, Trp222, and Gly224; Pi bond and alkyl bond: Phe70, Arg71, Phe101, Ile142, His158, Phe223, and Tyr255; those for salermide were as follows: H-bond: Gln140 and Tyr255; VW: Ala59, Ph70, Phe101, Arg105, Asn141, Val221, Trp222, Phe223, Gly224, Glu225, and Asn226; Pi bond: Ile142, His158, Leu227, and Tyr255; and those for Inauhzin were as follows: H-bond: Arg71; VW: Val67, Thr69, Phe70, Ala82, Gln83, Arg105, Gln140, Asn141, Ile142, His158, and Phe223; Pi bond and alkyl bond: Ala59, Arg71, and Tyr255; other bond: Arg71.

The occurrence counts of the key residues in at least 50% of the compounds against the SIRT5 protein were as follows: Val221 (14), Phe223 (14), Trp222 (13), Tyr255 (13), His158 (12), Ile142 (10), Ala82 (10), Phe70 (10), Tyr102 (9), Ala86 (8), Val220 (7), and Glu225 (7).

#### 3.3.5 SIRT6 and standard ligand dataset analysis

The docking score for the SIRT6 protein was in the range of −5.1 kcal/mol to −10.8 kcal/mol. The top three complexes against the SIRT6 protein were SirReal2, AK-7, and sirtinol, with docking scores of −10.8 kcal/mol, −9.8 kcal/mol, and −10.1 kcal/mol, respectively. The interactions recorded for SirReal2 were as follows: H-bond: Ala51 and Ser214; C–H bond: Gly64; VW: Glu20, Gly50, Gly52, Arg63, Trp69, Gln111, His131, Gly212, Thr213, Asn238, and Gln240; Pi bond and alkyl bond: Ala51, Asp61, Phe62, Pro65, Val113, Ile217, and Leu239; those for AK-7 were as follows: H-bond: Arg63, Trp69, and Trp186; Pi donor H bond: Gln111; VW: Lys13, Gly50, Asn112, His131, Ile183, Leu184, Asp185, Thr213, and Ser214; Pi bond and alkyl bond: Ala51, Phe62, Val113, Trp186, and Ile217; and those for sirtinol were as follows: H-bond: His131; Pi donor H-bond: Gln111; VW: Gly50, Arg63, Asn112, Val113, Ile183, Trp186, and Ile217; Pi bond: Ala51, Phe62, Trp69, His131, and Leu184.

The occurrence counts of the key residues in at least 50% of the compounds against the SIRT6 protein were as follows: Val221 (14), Phe223 (14), Trp222 (13), Tyr255 (13), His158 (12), Ile142 (10), Ala82 (10), Phe70 (10), Tyr102 (9), Ala86 (8), Val220 (7), and Glu225 (7).

**FIGURE 1:**
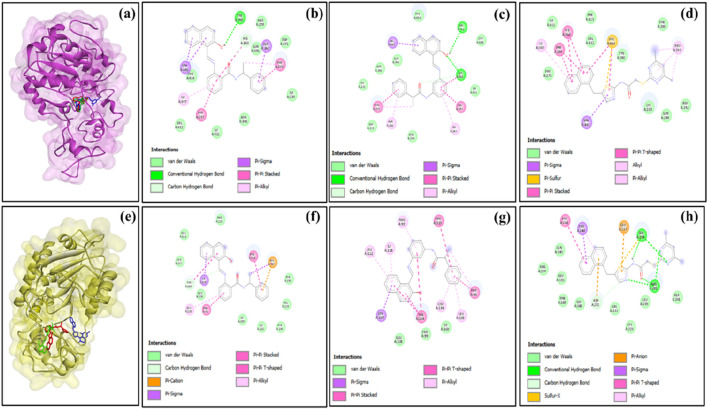
**(a)** Superimposition of sirtinol (red), salermide (green), and SirReal2 (blue) in the binding pocket of SIRT1 and 2D representation of the docked complexes **(b)** SIRT1–sirtinol, **(c)** SIRT1–salermide, and **(d)** SIRT1–SirReal2; **(e)** superimposition of sirtinol (red), salermide (green), and SirReal2 (blue) in the binding pocket of SIRT2 and 2D representation of the docked complexes **(f)** SIRT2–Sirtinol, **(g)** SIRT2–salermide, and **(h)** SIRT2–SirReal2.

**FIGURE 2:**
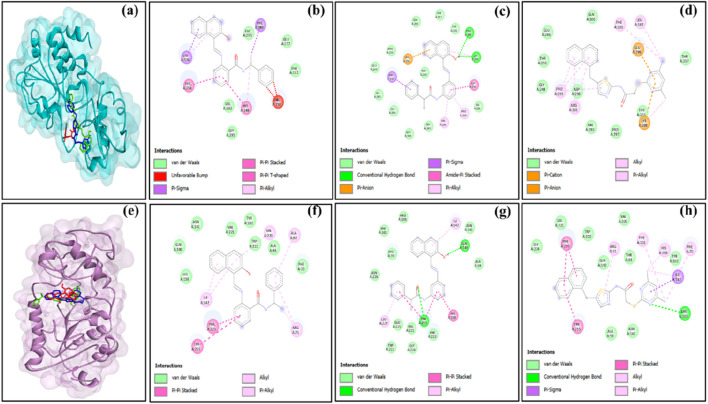
**(a)** Superimposition of sirtinol (red), salermide (green), and SirReal2 (blue) in the binding pocket of SIRT3 and 2D representation of the docked complexes **(b)** SIRT3–sirtinol, **(c)** SIRT3–salermide, and **(d)** SIRT3–SirReal2; **(e)** superimposition of sirtinol (red), salermide (green), and SirReal2 (blue) in the binding pocket of SIRT5 and 2D representation of the docked complexes **(f)** SIRT5–sirtinol, **(g)** SIRT5–salermide, and **(h)** SIRT5–SirReal2.

**FIGURE 3:**
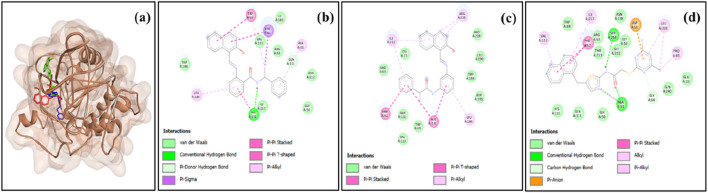
**(a)** Superimposition of sirtinol (red), salermide (green), and SirReal2 (blue) in the binding pocket of SIRT6 and 2D representation of the docked complexes **(b)** SIRT6–sirtinol, **(c)** SIRT6–salermide, and **(d)** SIRT6–SirReal2.

To streamline the study flow, we focused on the compounds that exhibited positive outcomes for more than one SIRT isoform. This step provided uniform treatment and easy comparison across the SIRT isoforms for different protein–ligand complexes. Based on the molecular docking analysis of 15 standard compounds against SIRT1, SIRT2, SIRT3, SIRT5, and SIRT6 proteins, we observed that the standard ligand sirtinol (PubChem ID: 2827646) produced significant results on SIRT1, SIRT2, and SIRT3. Sirtinol exhibited the lowest docking score on SIRT2 targets among all other standard ligands. Another standard compound, salermide, also exhibited potential outcome on multiple SIRT isoforms, i.e., SIRT1, SIRT2, SIRT3, and SIRT5. Salermide exhibited the lowest docking score and favorable binding interactions with SIRT1, SIRT3, and SIRT5. Similarly, another standard ligand, i.e., SirReal2, outperformed on SIRT3, SIRT5, and SIRT6 protein targets. SirReal2 showed the lowest docking score on the SIRT6 isoform. From all these observations, it could be noted that a few other standard ligands also appeared in the top-three list of protein–ligand complexes, but sirtinol, salermide, and SirReal2 were the compounds that predominantly performed better in maintaining the uniformity of the protocol. The 2D interactions of the multi-target selected compounds (sirtinol, salermide, and SirReal2) with five SIRT isoforms are shown in Figures 1–3. Furthermore, key interacting residues, stability, and conformational changes in each SIRT isoform upon binding of these three ligands were confirmed using the MDS approach.

### 3.4 Molecular dynamics simulation

MDS enables us to mimic a realistic protein–ligand complex environment by analyzing the dynamic nature of both the protein and ligand. Sirtinol, salermide, and SirReal2 standard ligands bound with SIRT1, SIRT2, SIRT3, SIRT5, and SIRT6 were further considered for MDS studies. In addition to these protein–ligand complexes, all five SIRT isoforms were also considered in the apo state (without any bound ligand) for comparison with other systems. This step was performed using the GROMACS 23.1 tool for 200 ns for all systems, and the trajectories were analyzed using various methods, such as RMSD, RMSF, RG, SASA, cluster analysis, PCA, DCCM, DSSP, number of H-bonds, percentage of H-bond occupancy, and per-residue energy decompositions.

#### 3.4.1 RMSD trajectory analysis

An RMSD plot represents the deviation of backbone atoms from their initial positions throughout the simulation. In general, lower RMSD values correspond to greater stability of the system.

**FIGURE 4:**
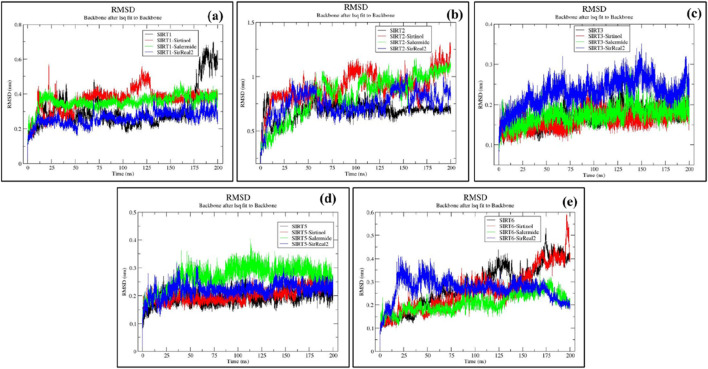
Representation of RMSD plots for **(a)** SIRT1, **(b)** SIRT2, **(c)** SIRT3, **(d)** SIRT5, and **(e)** SIRT6 in apo form and the top three standard ligand-bound states.

For the SIRT1 protein, the ranges of all four trajectories (SIRT1, SIRT1–sirtinol, SIRT1–salermide, and SIRT1–SirReal2) were from 0.1 to 0.6 nm SIRT1 apo form and SIRT1–sirtinol were found to have unstable trajectories with many major and minor peaks, whereas SIRT1–salermide and SIRT1–SirReal2 trajectories were comparatively much more stable. However, a few minor peaks could be observed in SIRT1–salermide and SIRT1–SirReal2 complexes. The order of stability from lower to higher on the SIRT1 protein could be concluded based on the average RMSD values, such as SIRT1 (0.3076 ± 0.110) nm, SIRT1–sirtinol (0.3641 ± 0.065) nm, SIRT1–salermide (0.3532 ± 0.040) nm, and SIRT1-SirReal2 (0.2595 ± 0.036) nm (Supplementary Table S4). From the above analysis, SIRT1–SirReal2 was found to be more stable than SIRT1 apo form and holo form complexes. The detailed representation of SIRT1 trajectories is shown in Figure 4a.

The trajectories on the SIRT2 protein ranged from 0.3 to 1.3 nm SIRT2 in the apo state exhibited an unstable trajectory until 50 ns, after which it remained stable until 200 ns, with RMSD values even better than those of other holoform complexes. The holoform trajectories (SIRT2–sirtinol, SIRT2–salermide, and SIRT2–SirReal2) exhibited many major and minor peaks, resulting in fluctuations throughout the simulation. The average RMSD values of the SIRT2 protein were estimated as SIRT2 (0.6859 ± 0.069) nm, SIRT2–sirtinol (0.9017 ± 0.142) nm, SIRT2–salermide (0.8294 ± 0.193) nm, and SIRT2–SirReal2 (0.7568 ± 0.114) nm (Supplementary Table S4). Based on the above analysis, SIRT2 in the apo state was found to be more stable, followed by SIRT2–SirReal2, SIRT2–salermide, and SIRT2–sirtinol (Figure 4b).

The trajectories on the SIRT3 protein ranged from 0.1 to 0.3 nm. Here, SIRT3–SirReal2 was observed as the most unstable trajectory, whereas the remaining three trajectories overlapped with each other with minute differences. The same observation could be noted with estimated average RMSD values as SIRT3 (0.1692 ± 0.022) nm, SIRT3–sirtinol (0.1603 ± 0.022) nm, SIRT3–salermide (0.1739 ± 0.022) nm, and SIRT3–SirReal2 (0.2296 ± 0.034) nm (Supplementary Table S4). Upon comparing all four trajectories, average RMSD values of SIRT3 in apo form and SIRT3–sirtinol are almost equal, making them equally stable, followed by SIRT3–salermide with a minute difference in average RMSD values from the former two trajectories and SIRT3–SirReal2 holo form with considerable differences from other systems (Figure 4c).

For the SIRT5 protein target, the trajectories ranged from 0.1 to 0.4 nm. SIRT5 apo form and SIRT5–sirtinol holo form acquired similar trajectories, with minor ignorable peaks, whereas SIRT5–salermide and SIRT5–SirReal2 exhibited considerable changes throughout the simulation. Among SIRT5–salermide and SIRT5–SirReal2, the former complex produced a trajectory with a higher RMSD value than the latter complex. The average RMSD values for apo and holo systems were found as follows: SIRT5 (0.1950 ± 0.025) nm, SIRT5–sirtinol (0.2008 ± 0.024) nm, SIRT5–salermide (0.2687 ± 0.040) nm, and SIRT5–SirReal2 (0.2210 ± 0.025) nm (Supplementary Table S4). Based on average RMSD values of these systems, it can be observed that SIRT5 in apo form and SIRT5–sirtinol are almost equally stable, followed by SIRT5–SirReal2 and SIRT5–salermide (Figure 4d).

For the SIRT6 protein, all apo and holo trajectories fluctuated a lot, ranging from 0.1 to 0.6 nm. In SIRT1 apo and SIRT6–sirtinol, no stable phase was attained during the simulation. The RMSD plots showed a continuous increase over time, reaching a maximum RMSD value of 0.6 nm. Among SIRT6–salermide and SIRT6-SirReal2 holo forms, SIRT6–salermide exhibited stable trajectory than SIRT6–SirReal2. The average RMSD values were observed as follows: SIRT6 (0.2809 ± 0.092) nm, SIRT6–sirtinol (0.2607 ± 0.091) nm, SIRT6–salermide (0.2083 ± 0.040) nm, and SIRT6–SirReal2 (0.2687 ± 0.046) nm (Supplementary Table S4). Upon comparing all these trajectories, the SIRT6–salermide holo system was found to be more stable, followed by SIRT6–SirReal2, SIRT6–sirtinol, and SIRT6 apo form (Figure 4e).

#### 3.4.2 RMSF trajectory analysis

RMSF analysis allows us to assess the protein’s flexibility and the fluctuations of each residue throughout the simulation period. Unstructured loops are generally observed to fluctuate more than the secondary structural elements, such as alpha-helix and beta-sheets. In general, lower RMSF values correspond to lesser fluctuations in c-alpha atoms of a protein and favor the system’s stability.

**FIGURE 5:**
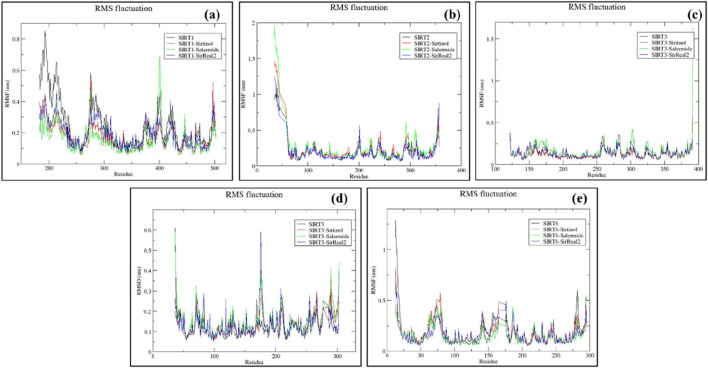
Representation of RMSF plots for **(a)** SIRT1, **(b)** SIRT2, **(c)** SIRT3, **(d)** SIRT5, and **(e)** SIRT6 in apo form and the top-three standard ligand-bound states.

In the SIRT1 target, the RMSF plots ranged from 0.06 to 0.8 nm in all four systems. Residue numbers 183–244 were observed as a flanking region of the protein, with most of this region located away from the binding pocket in a loop conformation. Therefore, large fluctuations were observed in the initial part of the RMSF plots. Subsequently, residues 272–295 and 394–403 were identified as additional fluctuating regions of the SIRT protein, and these regions were located away from the key binding residues. The average RMSF values were noted as follows: SIRT1 (0.2465 ± 0.151) nm, SIRT1–sirtinol (0.1732 ± 0.084) nm, SIRT1–salermide (0.1590 ± 0.087) nm, and SIRT1–SirReal2 (0.1876 ± 0.084) nm (Supplementary Table S4). Based on the above outcomes, it can be observed that SIRT1–salermide exhibited minimum residual fluctuations, followed by SIRT1–sirtinol, SIRT1–SirReal2, and SIRT1 apo form (Figure 5a).

Residues 34–59, located in the terminal region, showed fluctuations; beyond this region, fluctuations were reduced in all four trajectories. The RMSF values of the SIR2 apo form and holo form ranged from 0.06 to 0.6. The overall RMSF plots for all four trajectories were within an acceptable range. The major fluctuating region comprised residues 290–314, which were located away from the binding pocket as none of the compounds interacted with the residues in this region. The average RMSF values obtained during the simulation were as follows: SIRT2 (0.2111 ± 0.179) nm, SIRT2–sirtinol (0.2342 ± 0.229) nm, SIRT2–salermide (0.2584 ± 0.279) nm, and SIRT2–SirReal2 (0.2023 ± 0.184) nm (Supplementary Table S4). Based on average RMSF values and trends in the trajectory, it can be concluded that SIRT1–SirReal2 and SIRT2 apo form were almost equally stable, with lesser residual fluctuations, followed by SIRT2–sirtinol and SIRT2–salermide (Figure 5b).

For the SIRT3 target, RMSF values were found in an acceptable range from 0.06 to 0.4 nm. The common fluctuating region across all four trajectories was found between 254 and 307 residues, with a few minor dips and peaks in between. The average RMSF values for the SIRT3 protein were observed as follows: SIRT3 (0.1308 ± 0.060) nm, SIRT3–sirtinol (0.1288 ± 0.058) nm, SIRT3–salermide (0.1645 ± 0.110) nm, and SIRT3–SirReal2 (0.1391 ± 0.054) nm (Supplementary Table S4). Based on these observations, the stability of different apo and holo forms belonging to the SIRT3 protein was as follows: SIRT3–sirtinol was the most stable, followed by the SIRT3 apo form, SIRT3–SirReal2, and SIRT3–salermide. Notably, the average RMSF values for SIRT3–sirtinol, SIRT3 apo form, and SIRT3–SirReal2 were close enough (Figure 5c).

The RMSF graph on the SIRT5 protein ranged from 0.05 to 0.59 nm. However, there was no well-defined fluctuating region, but residue 175 exhibited maximum peaks in all four trajectories. The average RMSF plots were calculated as follows: SIRT5 (0.1268 ± 0.058) nm, SIRT5–sirtinol (0.1342 ± 0.055) nm, SIRT5–salermide (0.1483 ± 0.068) nm, and SIRT5–SirReal2 (0.1331 ± 0.062) nm (Supplementary Table S4). On comparing these values and trends in RMSF plots, it can be concluded that all four trajectories followed the same trend of fluctuations, making the SIRT5 apo form the least fluctuating, followed by SIRT5–sirtinol, SIRT5–SirReal2, and SIRT5–salermide (Figure 5d).

For the SIRT6 protein, the RMSF graph fluctuated in the range of 0.05–0.57 nm. Residues 62–82 and 139–188 were identified as the two fluctuating regions observed in all four trajectories. Overall, RMSF values were within the acceptable limits. The calculated RMSF values were as follows: SIRT6 (0.1749 ± 0.147) nm, SIRT6–sirtinol (0.1817 ± 0.112) nm, SIRT6–salermide (0.1512 ± 0.090) nm, and SIRT6–SirReal2 (0.1659 ± 0.096) nm (Supplementary Table S4. The trend of stability and least fluctuations can be predicted as follows: SIRT6–salermide is the most stable, followed by SIRT6–SirReal2, SIRT6 apo form, and SIRT6–sirtinol (Figure 5e).

Overall, the residues identified during the docking stage exhibited low to moderate fluctuations in the RMSF analysis. This indicated persistent docking interactions and the stable dynamic environment across the SIRT isoforms.

#### 3.4.3 RG analysis

RG determines the distribution of a system’s mass around its rotational axis, which further aids in estimating the compactness and folding pattern of the system. In general, lower RG values favor a more compact and stable system.

**FIGURE 6:**
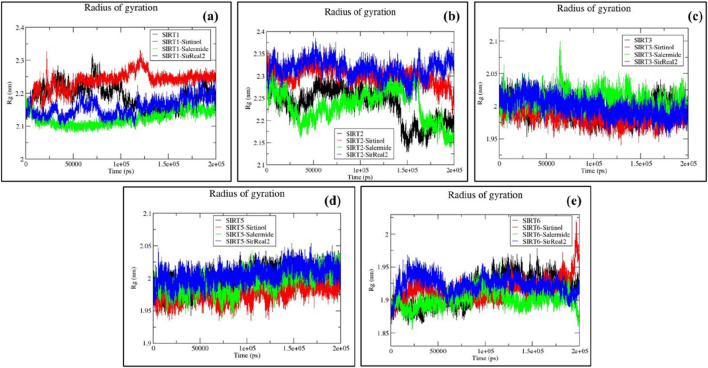
Representation of RG plots for **(a)** SIRT1, **(b)** SIRT2, **(c)** SIRT3, **(d)** SIRT5, and **(e)** SIRT6 in apo forms and the top three standard ligand-bound states.

For the SIRT1 target, the average RG values were obtained as follows: SIRT1 (2.1915 ± 0.035) nm, SIRT1–sirtinol (2.2366 ± 0.029) nm, SIRT1–salermide (2.1230 ± 0.021) nm, and SIRT1–SirReal2 (2.1562 ± 0.025) nm (Supplementary Table S4). The average RG values were similar, allowing us to assess the compactness of the system, although the trends in the RG plots were slightly comparative. We observed that SIRT1–salermide exhibited the minimum compactness and maximum stability, followed by SIRT1–SirReal2, SIRT1 apo form, and SIRT1–sirtinol (Figure 6a).

Similarly, the average RG values on the SIRT2 protein were as follows: SIRT2 (2.2350 ± 0.038) nm, SIRT2–sirtinol (2.2999 ± 0.018) nm, SIRT2–salermide (2.2332 ± 0.035) nm, and SIRT2–SirReal2 (2.3164 ± 0.020) nm (Supplementary Table S4). The trajectories for SIRT2 apo form and SIRT2–salermide were highly fluctuating, with many major and minor peaks, whereas trajectories for SIRT2–sirtinol and SIRT–SirReal2 overlapped with each other with similar patterns and lesser fluctuations. Upon comparing average values and trajectory patterns of apo form and holo forms, SIRT2 apo form and SIRT2–salermide exhibited equal compactness and stability. SIRT2–salermide behaved in the same way as the apo form. SIRT2–sirtinol and SIRT2–SirReal2 exhibited stable trajectories, but with higher average RG values, making these systems less compact and stable (Figure 6b).

For the SIRT3 protein, the average RG values were estimated as follows: SIRT3 (1.9954 ± 0.013) nm, SIRT3–sirtinol (1.9852 ± 0.011) nm, SIRT3–salermide (2.0104 ± 0.014) nm, and SIRT3–SirReal2 (1.9982 ± 0.014) nm (Supplementary Table S4). The RG plots for the SIRT3 apo and holo forms also exhibited a similar pattern. SIRT3–salermide was the only trajectory showing a major peak at 64 ns; the remaining systems exhibited minor peaks within acceptable ranges. Therefore, we could conclude that compactness and stability of the SIRT3 apo form, SIRT3–sirtinol, and SIRT3–SirReal2 were almost the same, whereas SIRT3–salermide was comparably more unstable than the former systems (Figure 6c).

For the SIRT5 protein, all four systems exhibited a similar pattern, although the RG values differed from one another. The average RG values were obtained as follows: SIRT5 (2.0003 ± 0.014) nm, SIRT5–sirtinol (1.9778 ± 0.013) nm, SIRT5–salermide (1.9946 ± 0.016) nm, and SIRT5–SirReal2 (2.0051 ± 0.013) nm (Supplementary Table S4). The average RG values were very close to each other, so it was difficult to assess compactness and stability across different systems in the SIRT5 protein (Figure 6d).

The average RG values on the SIRT6 protein were estimated as follows: SIRT6 (1.9151 ± 0.022) nm, SIRT6–sirtinol (1.9154 ± 0.017) nm, SIRT6–salermide (1.9011 ± 0.012) nm, and SIRT6–SirReal2 (1.9231 ± 0.013) nm (Supplementary Table S4). Here, SIRT6–salermide trajectories exhibited similar patterns to that of SIRT6 apo form, whereas the others remained little different, with a few minor peaks. Similar to SIRT5, average RG values were close to each other, making it difficult to assess the stability and compactness of the systems (Figure 6e).

#### 3.4.4 SASA analysis

SASA analysis estimates the surface area of any system that is accessible to solvent molecules. It also provides insights into the behavior of biomolecules in solution, including aspects such as folding, stability, and interactions with other molecules. A lower SASA value determines a more stable system.

**FIGURE 7:**
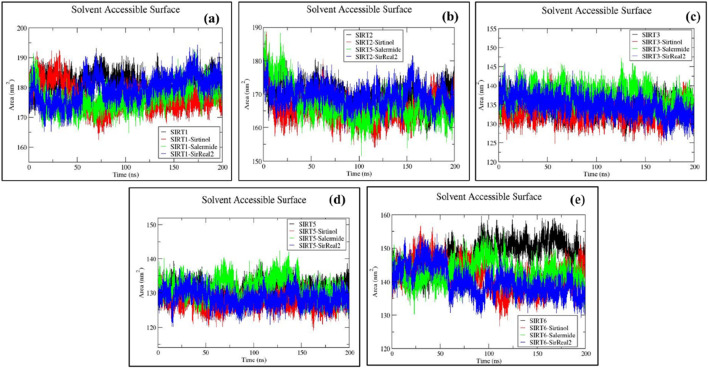
Representation of SASA plots for **(a)** SIRT1, **(b)** SIRT2, **(c)** SIRT3, **(d)** SIRT5, and **(e)** SIRT6 in apo forms and the top three standard ligand-bound states.

The average SASA values for the SIRT1 protein were determined as follows: SIRT1 (181.43 ± 3.27) nm^2^, SIRT1–sirtinol (176.73 ± 4.31) nm^2^, SIRT1–salermide (176.79 ± 3.98) nm^2^, and SIRT1–SirReal2 (179.47 ± 4.30) nm^2^ (Supplementary Table S4). Based on average SASA values, we could conclude that SIRT1–sirtinol and SIRT1–salermide were equally stable, followed by SIRT1–SirReal2 and SIRT1 apo form (Figure 7a).

For the SIRT2 protein, the average SASA values were reported as follows: SIRT2 (169.11 ± 3.56) nm^2^, SIRT2–sirtinol (165.76 ± 3.98) nm^2^, SIRT2–salermide (166.88 ± 5.02) nm^2^, and SIRT2–SirReal2 (169.45 ± 3.14) nm^2^ (Supplementary Table S4). Based on average SASA values, it could be concluded that SIRT2–sirtinol and SIRT2–salermide were the most stable complexes, followed by SIRT2 apo form and SIRT2–salermide (Figure 7b).

Similarly, SIRT3 (134.43 ± 2.08)n m^2^, SIRT3–sirtinol (133.07 ± 2.32) nm^2^, SIRT3–salermide (137.12 ± 2.51) nm^2^, and SIRT3–SirReal2 (134.76 ± 2.60) nm^2^ were the average values against SIRT3 protein systems (Supplementary Table S4). Upon comparing average SASA values, it could be concluded that SIRT3–sirtinol was the most stable complex, whereas SIRT3–SirReal2 and SIRT3 apo form were equally stable, with close average SASA values, followed by the SIRT3–salermide complex (Figure 7c).

The average SASA values for the SIRT5 protein were estimated as follows: SIRT5 (131.02 ± 2.12) nm^2^, SIRT5–sirtinol (127.60 ± 2.18) nm^2^, SIRT5–salermide (131.50 ± 2.94) nm^2^, and SIRT5–SirReal2 (128.54 ± 2.32) nm^2^ (Supplementary Table S4). Based on average SASA values and the trajectory trends, it can be concluded that SIRT5–sirtinol was most stable, followed by SIRT5–SirReal2. At the same time, SIRT5 apo form and SIRT5–salermide were considered equally stable complexes (Figure 7d).

Additionally, fluctuating SASA plots were obtained against the SIRT6 protein target, with average SASA values of SIRT6 (147.74 ± 4.44) nm^2^, SIRT6–sirtinol (141.92 ± 4.25) nm^2^, SIRT6–salermide (142.55 ± 3.31) nm^2^, and SIRT6–SirReal2 (140.07 ± 4.12) nm^2^ (Supplementary Table S4). Based on average SASA values and the trajectory patterns, it could be observed that SIRT6 in holo form exhibited lower and more consistent average SASA values in all three cases, i.e., SIRT6–SirReal2, SIRT6–sirtinol, and SIRT6–salermide. However, the SIRT6 apo form exhibited a comparably higher average SASA value, making it more unstable than the holo forms (Figure 7e).

#### 3.4.5 PCA

PCA confirms the overall movements of C-alpha (C-α) atoms that signify the deformation modes and activity of a protein (Pant et al., 2020). In general, a narrow conformational space in a PCA plot corresponds to a more stable system. PCA was conducted to identify the eigenvectors responsible for the global motion of the protein, thereby ensuring the dynamic nature of different systems. The global motion of a protein is primarily determined by the first few eigenvectors. To identify the eigenvectors, we performed diagonalization of the matrix and selected and plotted PC1 and PC2, which accounted for more than 80% of the protein’s motion.

**FIGURE 8:**
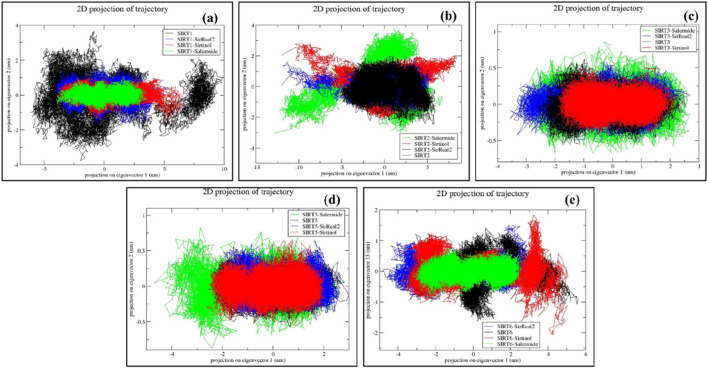
Representation of PCA plots for **(a)** SIRT1, **(b)** SIRT2, **(c)** SIRT3, **(d)** SIRT5, and **(e)** SIRT6 in apo form and the top three standard ligand-bound states.

For SIRT1 protein targets, the order of conformational space formed (lower to higher) was as follows: SIRT1–salermide, SIRT1–sirtinol, SIRT1–SirReal2, and SIRT1 apo form. Among all the systems, SIRT1–salermide could be considered more stable based on PCA (Figure 8a). For SIRT2, the order and stability of different apo forms and holo forms were as follows: SIRT2 in apo form was most stable, followed by SIRT2–SirReal2, SIRT2–sirtinol, and SIRT2–salermide. This suggests that when any of these three standard ligands bind with the SIRT2 protein, the stability is reduced (Figure 8b). The order of lower conformational states on the SIRT3 protein was as follows: SIRT3–Sirtinol was most stable, followed by SIRT3 apo form, SIRT3–SirReal2, and SIRT3–salermide. Surprisingly, the order of stability on SIRT3 and SIRT5 was the same as described above (Figures 8c,d). Furthermore, SIRT6–salermide, SIRT6–SirReal2, SIRT6 apo form, and SIRT6–sirtinol were used to assess the stability of different standard ligands on the SIRT6 protein (Figure 8e).

#### 3.4.6 DCCM analysis

The critical residues belonging to the ligand-binding site (hot spots) may be responsible for modulating the protein’s activity and substrate specificity (Yu and Dalby, 2020). To examine these critical residues, DCCM plots were generated to understand the relative motion and the degree of correlated and anti-correlated motion of C-alpha residues for all apo- and holoform trajectories. In a DCCM plot, the red region (+1 to 0) indicates positively correlated motion of the residues, where the residues are expected to move in the same direction. The blue region (−1 to 0) indicates the negatively correlated or anti-correlated motion of the protein residues, where the residues are expected to move in opposite directions. Finally, the white (0) region represents the neutral or zero-correlated residues.

To interpret the DCCM plots, we used a novel strategy by calculating the percentage area of correlated, anti-correlated, and neutral residues and comparing them among different systems. To calculate the percentage area of correlated, anti-correlated, and neutral regions in a DCCM, each matrix element, representing the correlation between residue pairs, was classified based on its correlation value. Values greater than +0.05 were considered correlated, values less than −0.05 were anti-correlated, and values between −0.05 and +0.05 were considered neutral. After categorizing all matrix values, the total count in each category was divided by the total number of matrix elements and multiplied by 100 to obtain the respective percentages. The complete protocol for plotting DCCM and calculating the percentage area is provided in our GitHub repository. The detailed percentages of correlated, anti-correlated, and neutral areas occupied by different systems are mentioned in Supplementary Table S5. A system can be stabilized by either correlated or anti-correlated motion of the protein. Unfortunately, we could not draw definitive conclusions about the stability of the system based on DCCM plots. In the subsequent section, we report the obtained percentage area of correlated, anti-correlated, and neutral residues in different dynamic systems.

In the first isoform of SIRTs, i.e., SIRT1, the percentage area of the correlated region was found to be maximum in the SIRT1 apo form (53.96%), followed by SIRT1–sirtinol (51.75%), SIRT1–salermide (50.97%), and SIRT1–SirReal2 (50.64%), whereas the percentage area of the anti-correlated region was found to be SIRT1 apo form (41.11%), followed by SIRT1–sirtinol (43.54%), SIRT1–salermide (44.38%), and SIRT1–SirReal2 (44.41%). It could be predicted that the SIRT1–salermide complex achieved stability due to less correlated motion and more anti-correlated motion during the simulation period. The same observation can be applied to other SIRT1-based systems also (Supplementary Figure S2a–S2d).

For the SIRT2 protein, the percentage area of correlation was observed as SIRT2 apo (53.82%), SIRT2–sirtinol (51.75%), SIRT2–salermide (52.89%), and SIRT2–SirReal2 (52.33%), whereas the percentage area of anti-correlation was observed as SIRT2 apo (41.610%), SIRT2–sirtinol (44.08%), SIRT2–salermide (42.70%), and SIRT2–SirReal2 (43.72%). From the previous analysis, we concluded that the apo form of SIRT2 performed better than the holo forms. The above-mentioned DCCM trends indicated that the SIRT2 protein was stabilized more by correlated motion than by anti-correlated motions during the simulation period (Supplementary Figure S2e–S2h).

For the SIRT3 protein, the percentage area of correlation was observed as SIRT3 apo (50.52%), SIRT3–sirtinol (49.86%), SIRT3–salermide (50.33%), and SIRT3–SirReal2 (50.34%), whereas the percentage area of anti-correlation was observed as SIRT3 apo (45.27%), SIRT3–sirtinol (46.24%), SIRT3–salermide (44.79%), and SIRT3–SirReal2 (45.81%). According to the previous analysis, sirtinol acted as a more stable standard compound on the SIRT3 target. This might be due to less correlated and more anti-correlated motion in the SIRT3 protein during the simulation period. However, the differences in the percentage of correlated and anti-correlated motion in DCCM plots were minor for other SIRT3-based systems (Supplementary Figure S3a–S3d).

For the SIRT5 protein, the percentage area of correlation was observed as SIRT5 apo (49.31%), SIRT5–sirtinol (50.02%), SIRT5–salermide (49.12%), and SIRT5–SirReal2 (49.13%), whereas the percentage area of anti-correlation was observed as SIRT5 apo (47.09%), SIRT5–sirtinol (45.47%), SIRT5–salermide (45.81%), and SIRT5–SirReal2 (47.11%). From previous analyses, sirtinol exhibited better performance on the SIRT5 target than the other standard compounds, which may be attributed to greater correlated motion than anti-correlated motion on the SIRT5 target during the simulation period. However, the differences in the percentage of correlated and anti-correlated motion in DCCM plots were minor for other SIRT5-based systems (Supplementary Figure S3e–S3h).

Similarly, on the SIRT6 protein, the percentage area of correlation was observed as SIRT6 apo (51.41%), SIRT6–sirtinol (52.41%), SIRT6–salermide (51.22%), and SIRT6–SirReal2 (51.17%), whereas the percentage area of anti-correlation was observed as SIRT6 apo (43.78%), SIRT6–sirtinol (42.24%), SIRT6–salermide (43.83%), and SIRT6–SirReal2 (43.75%). Based on previous analysis, we concluded that SirReal2 exhibited greater stability in the binding pocket of the SIRT6 protein; this is likely because the reduced correlated motion stabilized the SIRT6 protein more than the anti-correlated motion during the simulation period. Again, the differences in the percentage of correlated and anti-correlated motion in DCCM plots were minor for other SIRT6-based systems (Supplementary Figure S4a–S4d). Overall, the DCCM analysis helped answer whether the correlation or the anti-correlation motion of the protein provided more stabilization to each SIRT isoform.

#### 3.4.7 DSSP analysis

DSSP analysis represents the change in secondary structures throughout the simulation period. To compare the extent of change in the secondary structure in each apo and holo form, we calculated the total number of structured residues and changes throughout the simulation period. We also plotted secondary structure timeline plots. Additionally, using SSTP, we can examine which regions or key residues of the protein undergo major changes in secondary structure elements.

##### 3.4.7.1 Secondary structure timeline plots

A typical SSTP represents how secondary structures, such as alpha-helix, beta-strand, beta-bridge, bend, turns, 3-helix, and coil, change with time. We have used SSTP to compare different protein–ligand complexes, and the most stable and least stable protein–ligand complexes have been identified.

For the SIRT1 protein, the major changes in secondary elements were observed in the core domain (360–410 residues) and tail region (440–513 residues), which show most ligand-induced differences. Residues 273–283 were completely stabilized from 40 ns onward until the end of the simulation upon binding of sirtinol to the SIRT1 protein. In the SIRT1–sirtinol complex, stabilized structured regions were identified by reducing coil/turn content. It may enhance rigidity and favor inhibition. The SIRT1–salermide complex maintained approximately the native state. The SIRT1–SirReal2 complex introduced flexibility and turns in the catalytic region (Supplementary Figure S5a–S5d).

In the SIRT2 protein, we could observe that residues 180–230 appeared essential to structural changes, and this region might be linked to conformational regulation. Specifically, SIRT2–sirtinol complex binding led to the destabilization of the complex, which reflected a weaker binding mode between them. Additionally, SIRT2–salermide preserved secondary structure elements with better stability, but less so than the SIRT2–SirReal2 complex. SIRT2–SirReal2 was the most stable complex with widespread β-sheet and structured region stabilization (Supplementary Figure S6a–S6d).

In the SIRT3 target, we identified that residues 220–290 were the structural hot spot of the SIRT3 protein. This region might be involved in ligand-induced changes. Among the different systems, the SIRT3–SirReal2 complex exhibited the strongest conservation of the secondary structure, especially β-sheets and helices, indicating greater stability and specific binding. SIRT3–salermide exhibited moderate stabilization by maintaining essential helices and some sheets. The SIRT3–sirtinol complex induced significant structural destabilization, indicating a weaker binding affinity (Supplementary Figure S7a–S7d).

In the SIRT5 protein, we found that residues 76–120 and 120–190 were significant for ligand-induced secondary element transitions. In addition, some structural changes were observed in the N-terminal region. SIRT5–sirtinol exhibited major structural changes in the mentioned residues, indicating this complex’s instability over others. The SIRT5–SirReal2 complex preserved secondary structures, mainly helices, and β-sheet in the mentioned region. This observation suggested that SIRT5–SirReal2 was the most stable complex with tight and ordered binding. The SIRT5–salermide complex offered moderate structural stabilization by retaining key elements with localized flexibility (Supplementary Figure S8a–S8d).

In the SIRT6 protein, we identified that residues 120–180 and 240–283 constantly showed ligand sensitivity, which was significant for ligand binding. SIRT6 in apo form exhibited many unstable structures, such as loops and turns, specifically in the regions 163–173 and 263–273. When the ligand was bound to the SIRT6 apo form, it was stabilized. In SIRT6–sirtinol, β-sheets and α-helices were maintained, specifically in regions 153–163, indicating a strong stabilizing interaction, possibly inhibitory. SIRT6–salermide offered moderate secondary structural preservation and moderate stabilization. SIRT6–SirReal2 exhibited more flexible loops and breakage in helices, especially in residues 120–180 and the C-terminal zones, suggesting ligand-induced structural modulation or partial destabilization (Supplementary Figure S9a–S9d).

##### 3.4.7.2 Number of structured residue analysis

Furthermore, we have calculated total residues (including coils), initial structures present at time t = 0 ns (initial structured residues), average structured residues, standard deviation (Std Dev), change from the initial state, overall percentage of structured residues (structured_%), and percentage of coils (Coil_%) (Supplementary Table S6). Since coils are considered under unstructured residues, they were excluded from the comparison across different systems in this study. Generally, for a system to be stable, it should have initial structured residues nearly equal to the average structured residues (change ≈0) (Chakraborty et al., 2013).

**FIGURE 9:**
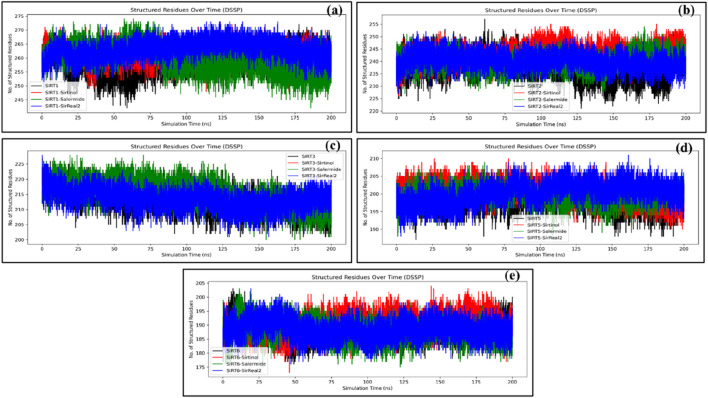
Representation of change in the total number of structured residues observed during the simulation period for **(a)** SIRT1, **(b)** SIRT2, **(c)** SIRT3, **(d)** SIRT5, and **(e)** SIRT6 in apo form and the top three standard ligand-bound states.

For the SIRT1 protein, changes in the structured residues from the initial state were found as follows: SIRT1 (−0.878), SIRT1–sirtinol (0.122), SIRT1–salermide (0.077), and SIRT1–SirReal2 (4.248). Based on the changes in structured residues from the initial state, the order of stability of different systems was SIRT1–salermide, being closer to the initial state, i.e., more stable, followed by SIRT1–sirtinol, SIRT1 apo form, and SIRT1–SirReal2. The change in the structured residues from the initial state was almost equal for SIRT1–Sirtinol and SIRT1 apo form but varied greatly for SIRT1–SirReal2 (Figure 9a). For the SIRT2 protein, the change in the total number of structured residues from the initial state was observed as follows: SIRT2 (6.013), SIRT2–sirtinol (7.453), SIRT2–salermide (5.134), and SIRT2–SirReal2 (3.508). Based on this, we could predict the order of stability as SIRT2–SirReal2 with a minimum difference from the initial state, followed by SIRT2–salermide, SIRT2 apo form, and SIRT2–sirtinol. The stability of the SIRT2 protein could clearly be observed based on changes in the total number of structured residues (Figure 9b).

Similarly, for the SIRT3 protein, the change in the total number of structured residues was found to be SIRT3 (−4.806), SIRT3–sirtinol (−2.376), SIRT3–salermide (−2.376), and SIRT3–SirReal2 (−2.531). It was difficult to draw conclusions on the stability of the apo and holo forms on the SIRT3 target because the changes in the total number of structured residues in SIRT3–SirReal2, SIRT3–salermide, and SIRT3–sirtinol were close to each other, whereas in the SIRT3 apo form, they could be well differentiated. We could conclude that SIRT3 became stable upon ligand binding based on this analysis (Figure 9c). The change in the total number of structured residues on the SIRT5 protein was observed as follows: SIRT5 (−4.956), SIRT5–sirtinol (−2.487), SIRT5–salermide (−1.453), and SIRT5–SirReal2 (−0.615). The order of stability based on this analysis could be represented as follows: SIRT5–SirReal2 being most stable, followed by SIRT5–salermide, SIRT5–sirtinol, and SIRT5 apo form (Figure 9d). Against the SIRT6 protein, SIRT6–SirReal2 was considered the most stable, followed by the SIRT6 apo form, SIRT6–salermide, SIRT6–SirReal2, and SIRT6–sirtinol. The stability order was defined based on the change in the total number of structured residues on the SIRT6 protein as SIRT6 (0.832), SIRT6–sirtinol (2.211), SIRT6–salermide (0.846), and SIRT6–SirReal2 (−0.663) (Figure 9e).

#### 3.4.8 Cluster analysis

**FIGURE 10:**
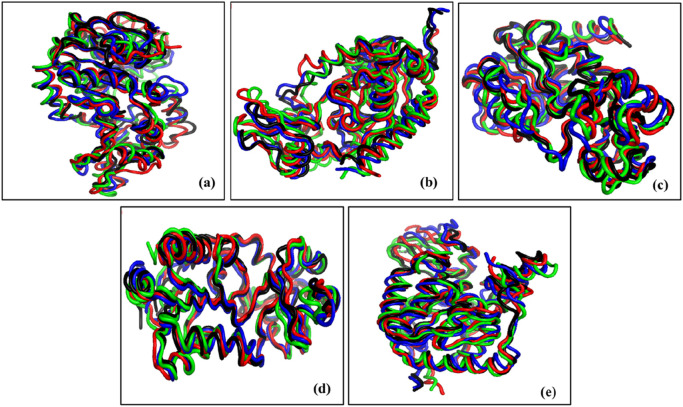
Conformational changes in the C-alpha atoms of **(a)** SIRT1, **(b)** SIRT2, **(c)** SIRT3, **(d)** SIRT5, and **(e)** SIRT6 in apo and standard ligand-bound states. In all the representations, conformation belonging to the apo state is shown in black, whereas holo forms, i.e., sirtinol, salermide, and SirReal2 ligands, are superimposed in red, green, and blue, respectively.

The gmx_cluster package in the GROMACS tool was used to analyze the different clusters formed in apo and holo forms studied in our study. We considered an RMSD cutoff of 0.2 nm for cluster analysis of all the trajectories. Generally, a stable trajectory can be considered, which has a minimum number of visited clusters, lower RMSD values, and a maximum number of similar conformations observed in the first cluster. In Supplementary Table S7, a detailed description of cluster analysis is provided. The representative structure of the first cluster from each trajectory was retrieved and superimposed with the apo and holo forms. This step was performed to observe the conformational changes of the C-alpha atoms in the respective systems during the simulation period (Figure 10).

**FIGURE 11:**
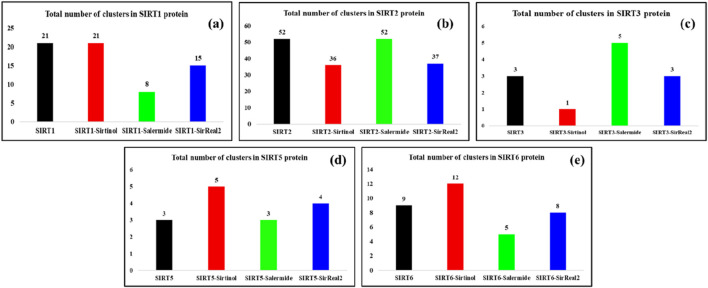
Representation of the total number of clusters formed during the simulation in **(a)** SIRT1, **(b)** SIRT2, **(c)** SIRT3, **(d)** SIRT5, and **(e)** SIRT6 in apo and standard ligand-bound states.

In the SIRT1 protein, the total numbers of clusters obtained by SIRT1 apo form, SIRT1–sirtinol, SIRT1–salermide, and SIRT1–SirReal2 were reported as 21, 21, 8, and 15, with average RMSD values of 0.0838, 0.0435, 0.0419, and 0.0419, respectively (Figure 11a). In addition, the numbers of structures in the first cluster were identified as 6,992, 15,197, 16,655, and 11,160 for SIRT1 apo form, SIRT1–sirtinol, SIRT1–salermide, and SIRT1–SirReal2, respectively. Among these systems, we could clearly observe the order of stability as SIRT1–salermide being the most stable complex, followed by SIRT1–SirReal2, SIRT1–sirtinol, and SIRT1 apo form.

In the SIRT2 protein, the number of clusters formed was more than that in SIRT1. The total number of clusters formed was 52, 36, 52, and 37 for SIRT2 apo form, SIRT2–sirtinol, SIRT2–salermide, and SIRT2–SirReal2, respectively, and the total numbers of structures in the first clusters were 6,463, 7,608, 4,914, and 7,739 in the same order (Figure 11b). Additionally, the average RMSD values in clusters were obtained as 0.0548, 0.0838, 0.0838, and 0.0437 for SIRT2 apo form, SIRT2–sirtinol, SIRT2–salermide, and SIRT2–SirReal2, respectively. Based on cluster analysis, we identified that SIRT2 in the apo state showed a greater number of conformations, indicating reduced stability. When the ligand binds to SIRT2, it gets stabilized except in the SIRT–salermide complex. This interpretation differs notably from the outcomes of other analyses. Therefore, SIRT2–SirReal2 and SIRT2–sirtinol could be considered equally stable, followed by SIRT2 apo form, and SIRT2–salermide is most unstable.

In the SIRT3 target, the conformational structures visited were minimal compared to those of other isoforms. The total number of clusters formed on the SIRT3 protein was 3, 1, 5, and 3, and the total number of structures in the first cluster was 19,956, 20,001, 18,916, and 19,549 for SIRT3 apo form, SIRT3–sirtinol, SIRT3–salermide, and SIRT3–SirReal2, respectively (Figure 11c). The average RMSD values were identified as 0.0230, 0.0213, 0.0419, and 0.0258, respectively. Based on the cluster analysis, we found that SIRT3–sirtinol was the most stable complex by attaining all the structures (20,000) throughout the simulation in a single cluster. SIRT3 in the apo form and the SIRT–SirReal2 complex were equally stable, with nearly equal clusters, average RMSD values, and total number of structures in the first cluster. SIRT–salermide did not deviate much in terms of stability from other systems.

For the SIRT5 protein, the number of clusters received in different systems was also lower. The total numbers of clusters acquired were 3, 5, 3, and 4, and the total numbers of structures in the first cluster were observed as 19,946, 19,925, 18,702, and 19,957 for SIRT5 apo form, SIRT5–sirtinol, SIRT5–salermide, and SIRT5–SirReal2, respectively (Figure 11d). The average RMSD values were 0.0258, 0.0241, 0.0419, and 0.0260, respectively. Based on cluster analysis, we observed that the number of clusters in SIRT5, SIRT5–sirtinol, and SIRT5–SirReal2 differed slightly, but the total number of structures in the first cluster and the average RMSD values were very similar. Therefore, all three complexes can be considered equally stable. However, the SIRT5–salermide complex exhibited minor variations, but these were not significant enough to warrant further focus.

Similarly, for SIRT6, the number of clusters obtained was also lower. These were 9, 12, 5, and 8, with total number structures in the first cluster as 9,381, 13,488, 18,881, and 15,123 for SIRT6 apo form, SIRT6–sirtinol, SIRT6–salermide, and SIRT6-SirReal2, respectively (Figure 11e). Furthermore, the average RMSD values were obtained as 0.0422, 0.0462, 0.0419, and 0.0419 in the same order. Based on cluster analysis, we concluded that SIRT6–salermide was the most stable complex with the maximum number of structures in the first cluster. SIRT6–SirReal2 was the second-best complex with 15,123 structures in the first cluster. Furthermore, SIRT6–SirReal2 and SIRT6 apo form obtained nearly equal numbers of clusters, but the total number of structures in the first cluster made SIRT6–SirReal2 more stable than the SIRT6 apo form.

#### 3.4.9 Estimation of the number of H-bonds

The total number of H-bonds formed during the simulation period was calculated for all SIRT holo forms using VMD software. We considered a 3.5-Å distance and a 30^◦^ angle with respective residues to analyze H-bond formation and percentage occupancy. A greater number of H-bonds indicates stable protein–ligand complexes. The total number of H-bonds formed throughout the simulation period is mentioned in Supplementary Table S4.

**FIGURE 12:**
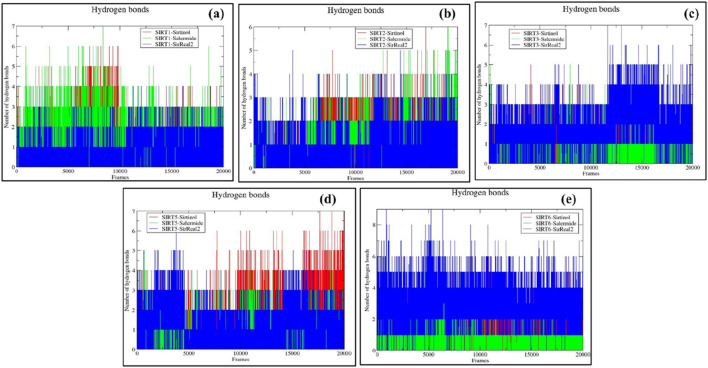
Representation of hydrogen bond analysis for **(a)** SIRT1, **(b)** SIRT2, **(c)** SIRT3, **(d)** SIRT5, and **(e)** SIRT6 in the top three standard holo states.

The total number of H-bonds formed during the simulation period for the SIRT1 protein was as follows: SIRT1–sirtinol (0–6), SIRT1–salermide (0–7), and SIRT1–SirReal2 (0–4). Based on this, we could provide the stability order as follows: SIRT1–salermide formed the maximum and consistent number of H-bonds, which favored its stability, followed by SIRT1–sirtinol and SIRT1–SirReal2 (Figure 12a). For the SIRT2 protein, the order of stability and the formation of H-bonds were found as follows: SIRT2–sirtinol (0–6), SIRT2–salermide (0–6), and SIRT2–SirReal2 (0–5) (Figure 12b). In the SIRT3 protein, SIRT3–SirReal2 (0–7) exhibited the maximum and most consistent number of H-bonds, followed by SIRT3–sirtinol (0–5) and SIRT3–salermide (0–5) (Figure 12c). SIRT5–sirtinol (0–7), SIRT5–salermide (0–5), and SIRT5-SirReal2 (0–6) were the formed hydrogen bonds in the SIRT5 target. SIRT5–sirtinol was more stable than the other two complexes (Figure 12d). Similarly, for the SIRT6 protein, the following numbers of H-bonds were formed: SIRT6–sirtinol (0–6), SIRT6–salermide (0–5), and SIRT6–SirReal2 (0–9). Among these holo forms, SIRT6–SirReal2 was more stable than the other two (Figure 12e). Based on H-bond analysis on all SIRT isoforms, it can be concluded that sirtinol formed a more stable complex on SIRT2 and SIRT5, salermide formed a stable complex with SIRT1, and SirReal2 formed a stable complex with SIRT3 and SIRT6. Furthermore, H-bond percentage occupancy was calculated using the VMD tool to evaluate the involvement of each residue in H-bond formation during the simulation period.

#### 3.4.10 H-bond percentage occupancy analysis

The H-bond percentage occupancy was calculated for all protein–ligand complexes. This step allowed us to identify the key residues involved in H-bond formation and quantify the contribution of each residue in the binding pocket throughout the simulation period in terms of percentage.

**FIGURE 13:**
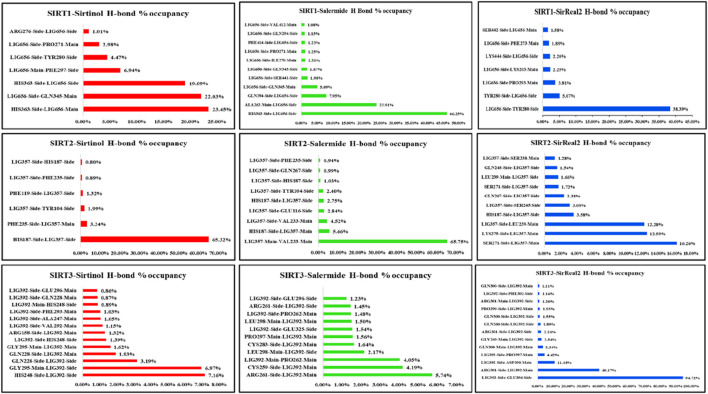
H-bond percentage occupancy analysis for SIRT1, SIRT2, and SIRT3 (the Y-axis shows the donor–acceptor pair, and the X-axis shows the percentage of H-bond occupancy).

The key residues with higher percentage occupancy were observed as follows: His363(with ligand main ring) (23.45%), Gln345 (22.03%), His363 (with ligand side ring) (19.9%), Phe297 (6.94%), and Tyr280 (2.98%) in the SIRT1–sirtinol complex. In the SIRT1–salermide complex, the major H-bond contributing residues were His363 (46.25%), Ala262 (23.91%), Gln294 (7.95%), and Gln345 (5.09%). In the SIRT1–SirReal2 complex, the key H-bond contributing residues were Tyr280 as the H-bond acceptor (38.39%), Tyr280 as the H-bond donor (5.07%), and Pro293 (3.81%). After comparing the percentage occupancy of all three SIRT1 holo forms, it could be concluded that Ala262, Tyr280, Pro293, Gln294, Phe297, Gln345, and His363 were common key residues for H-bond formation (Figure 13).

In the SIRT2–sirtinol complex, His187 (65.32%) and Phe235 (3.24%) were the major key residues in H-bond formation. In SIRT2–salermide, Val233 (35.75%), His187 with the main chain of the ligand (5.66%), Val233 (4.52), Glu116 (2.84%), His187 with the side chain of the ligand (2.75%), and Tyr104 (2.40%) were the major key residues in H-bond formation. In SIRT2–salermide, the major key residues in H-bond formation were Ser271 (16.26%), Lys275 (12.59%), Leu239 (12.28%), His187 (3.58%), and Ser245 (3.09%). After comparing all three SIRT2 holo forms, we concluded that the common key residues involved in the formation of H-bonds were Glu116, His187, Val233, Phe235, Leu239, Ser245, Ser271, and Lys275 (Figure 13).

In SIRT3–sirtinol, His248 (7.16%), Gly295 (6.97%), and Gln228 (3.19%) were the major key residues involved in H-bond formation. In SIRT3–salermide, the major H-bond contributing residues were Arg261 (5.74%), Cys259 (4.19%), Pro262 (4.05%), and Leu298 (2.17%). In SIRT1–SirReal2, the major key residues involved in H-bond formation were Glu296 (94.72%), Arg301 (40.17%), Asp290 (11.15%), Pro297 (4.42%), Gln300 (3.34%), Gly249 (2.54%), and Arg301 (2.24%). After comparing all three SIRT3 holo forms, we were able to identify the common residues involved in H-bond formation, such as Gln228, His248, Cys259, Arg261, Pro262, Asp290, Gly295, Leu296, Pro297, Leu298, Gln300, and Arg301(Figure 13).

**FIGURE 14:**
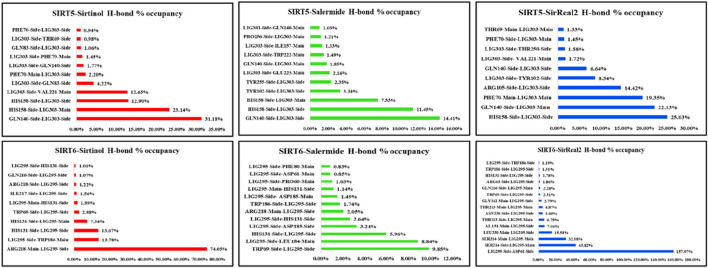
H-bond percentage occupancy analysis for SIRT5 and SIRT6 (the Y-axis shows the donor–acceptor pair, and the X-axis shows the percentage of H-bond occupancy).

In SIRT5–sirtinol, Gln140 (31.18%), His158 with the main chain of the ligand (23.14%), His158 with the side chain of the ligand (12.90%), Val221 (12.65%), Gln83 (4.22%), and Phe70 (2.20%) were residues involved in H-bond formation. In SIRT5–salermide, the major key residues for H-bond formation were Gln140 (14.41%), His158 with the side chain of the ligand (11.45%), His158 with the main chain of the ligand (7.53%), Tyr102 (3.34%), Tyr255 (2.35%), and Glu225 (2.16%). Similarly, in SIRT5–SirReal2, the key residues for H-bond formation were His158 (25.03%), Gln140 with the main chain of the ligand (22.13%), Phe70 (19.35%), Arg105 (14.42%), Tyr102 (8.54%), and Gln140 with the side chain of the ligand (6.64%). Upon comparing all the SIRT5-based holo forms, we identified the common key residues prominently involved in H-bond formation on the SIRT5 target as Phe70, Gln83, Tyr102, Arg105, Gln140, His158, Val221, Glu225, and Tyr255 (Figure 14).

In SIRT6–sirtinol, Arg218 (74.05%), Trp186 (13.78%), His131 with the side chain of ligand (13.67%), His131 with the main chain of ligand (7.34%), and Trp69 (2.88%) were observed as the key residues responsible for H-bond formation. In SIRT6–salermide, the residues involved in the formation of H-bonds were Trp69 (9.85%), Leu184 (8.84%), His131 as the donor (5.96%), Asp185 (3.24%), His131 as the acceptor (2.64%), and Arg218 (2.05). In SIRT6–SirReal2, Asp61 (157.97%), Ser214 residue’s side chain (43.82%), Ser214 residue’s main chain (32.08%), Leu239 (15.58%), Ala51 (7.04%), Thr213 residue’s side chain (4.87%), Asn238 (5.05%), Thr213 residue’s main chain (4.87%), Gly212 (3.79%), and Gln216 (2.28%) were identified as major residues responsible for the formation of H-bonds. Upon comparing the percentage occupancy in the SIRT6 protein, we identified the major key residues as Ala51, Asp61, Trp69, His131, Leu184, Asp185, Trp186, Gly212, Thr213, Ser214, Gln216, Arg218, Asn238, and Leu239 (Figure 14).

Overall, the H-bond occupancy pattern defined the isoform-specific interaction patterns that enabled answering the selectivity mechanism across SIRT isoforms. For example, SIRT1 complexes formed a stable H-bond with residues Ala262, Tyr280, Pro293, Gln294, Phe297, Gln345, and His363 present in the catalytic core of the SIRT1 isoform. In contrast, H-bonds in SIRT2 complexes were formed in the peripheral region with residues Glu116, His187, Val233, Phe235, Leu239, Ser245, Ser271, and Lys275, not specifically in the catalytic binding pocket of the SIRT2 protein, despite the same set of compounds. This observation suggests that selectivity is achieved when a standard compound forms a stable H-bond with catalytic core residues (SIRT1) and is reduced when the H-bonds shift toward non-catalytic regions (SIRT2). Similarly, other isoforms can also be observed to understand the selectivity nature based on the H-bond formations and conclude the predictive selective nature of the standard ligand set.

#### 3.4.11 MMPBSA and decomposition analysis

##### 3.4.11.1 TBFE analysis

Furthermore, TBFE was calculated for the top three standard ligands bound with SIRT1, SIRT2, SIRT3, SIRT5, and SIRT6. This analysis was performed to obtain the TBFE and the contribution of each SIRT-isoform-specific residue bound to the standard ligand molecule. Generally, a lower TBFE represents a stable protein–ligand complex. The complete information regarding different types of energies calculated in the MMPBSA analysis is mentioned in Supplementary Table S8.

For the SIRT1 protein, the observed TBFE values were SIRT1–sirtinol (−25.74 ± 2.45) kcal/mol, SIRT1–salermide (−27.24 ± 2.26) kcal/mol, and SIRT1–SirReal2 (−24.79 ± 2.59) kcal/mol. SIRT1–salermide, SIRT1–sirtinol, and SIRT1–SirReal2 represented the order of stability based on the calculated TBFE values. For the SIRT2 protein, the TBFE values were reported as follows: SIRT2–sirtinol (−22.06 ± 2.44) kcal/mol, SIRT2–salermide (−25.39 ± 4.94) kcal/mol, and SIRT2–SirReal2 (−30.49 ± 4.58) kcal/mol. Based on this, the predicted order of stability for the SIRT2 protein was SIRT2–SirReal2, SIRT2–salermide, and SIRT2–sirtinol.

The TBFE values for the SIRT3 target were as follows: SIRT3–sirtinol (−20.43 ± 2.20) kcal/mol, SIRT3–salermide (−18.84 ± 3.29) kcal/mol, and SIRT3–SirReal2 (−41.41 ± 3.70) kcal/mol. The TBFE of the SIRT3–SirReal2 complex was very low, making it much more stable, followed by SIRT3–sirtinol and SIRT3–salermide. Similarly, SIRT5–sirtinol (−42.24 ± 2.67) kcal/mol, SIRT5–salermide (−28.08 ± 3.30) kcal/mol, and SIRT5–SirReal2 (−26.83 ± 3.26) kcal/mol were the estimated TBFE values for the SIRT5 protein. Here, SIRT5–sirtinol exhibited a much lower TBFE value, making it more stable than the other two complexes.

For the SIRT6 protein, SIRT6–sirtinol (−26.17 ± 3.15) kcal/mol, SIRT6–salermide (−19.89 ± 2.47) kcal/mol, and SIRT6–SirReal2 (−58.55 ± 3.76) kcal/mol were the calculated TBFE values. The SIRT6–SirReal2 complex exhibited the lowest TBFE value of the other two. This trend was similar to that of the SIRT3 protein.

##### 3.4.11.2 Per-residue binding energy contribution by decomposition analysis

In addition to TBFE calculation, we also evaluated the contribution of each residue to TBFE using decomposition analysis. The gmx_MMPBSA tool was used for this purpose, which selected residues within a 4 Å distance between the receptor and ligand for decomposition analysis.

The selected residues for decomposition analysis in the binding pocket of the SIRT1 protein were noted as Pro207, Gly261, Ala262, Asp272, Phe273, Arg274, Tyr280, Phe297, Gln345, Asn346, Ile347, His363, Ile411, Val412, Phe413, Phe414, Leu418, Ser441, Lys444, Val445, and Arg446 for SIRT–sirtinol; Pro207, Ala262, Ser265, Ile270, Pro271, Asp272, Phe273, Arg274, Phe297, Gln345, Asn346, Ile347, Asp348, His363, Ile411, Val412, Phe413, Phe414, Leu418, Val445, Arg446, and Pro447 for SIRT1–salermide; and Thr200, Lys203, Phe273, Arg274, Tyr280, Pro293, Phe297, Gln345, Asn346, Ile347, His363, Ile411, Val412, Phe413, Phe414, and Val445 for SIRT1–SirReal2 complexes. Among all these residues, common residues that contributed significantly were Ala262, Phe273, Tyr280, Phe297, Ile347, His363, Ile411, Phe413, and Phe414. These residues could be considered major energy contributors in the top three SIRT1-bound complexes (Supplementary Figure S10a).

Similarly, for the SIRT2 target, the selected residues for decomposition analysis in the binding pocket were estimated as Phe96, Arg97, Tyr104, Leu107, Leu112, Pro115, Ile118, Phe119, Leu134, Leu138, Ile169, His187, Ile232, Val233, Phe234, and Phe235 for SIRT2–sirtinol; Phe96, Arg97, Pro99, Tyr104, Leu107, Glu108, His111, Leu112, Pro115, Glu116, Ile118, Phe119, Leu134, and Phe235 for SIRT2–salermide; and Arg174, Gln180, Glu185, Gly188, Thr189, His194, Arg201, Lys229, Asp231, Val233, Gly236, Glu237, Ser238, Leu239, Pro240, Arg242, and Phe243 for SIRT2–SirReal2. Here, the SIRT2–SirReal2 complex occupied a slightly different position than the other two, as described earlier. After comparing Supplementary Figure S10b, we concluded that the residues that were identified as major energy contributors were Phe96, Pro115, Phe119, Phe235, and Leu239 on SIRT2 targets.

For the SIRT3 protein, the selected residues for decomposition analysis in the binding pocket were identified as Phe157, Arg158, Glu177, Phe180, His248, Val292, Phe293, Phe294, Leu298, Val324, Glu325, and Pro326 for SIRT3–sirtinol; Phe186, Arg235, Glu246, Gly249, Thr250, Ala254, Thr255, Thr257, Gln260, Lys288, Pro289, Asp290, Val292, Glu296, Pro297, Pro299, Gln300, and Arg301 for SIRT3–salermide; and Arg235, Glu246, Gly249, Thr250, Ser253, Thr255, Gln260, Lys288, Asp290, Val292, Glu296, Pro297, Leu298, Pro299, Gln300, and Arg301 for SIRT3–SirReal2. In Supplementary Figure S10c, it could be observed that the major energy contributing residues were Phe157, Arg158, Phe180, Pro297, His248, Val292, Phe294, Leu298, Thr250, Ser253, Gln260, Glu296, Pro297, Pro299, Gln300, and Arg301. These residues could be considered significant for SIRT3 inhibitory activity.

For the SIRT5 protein, the selected residues within the pocket were observed as Ala59, Thr69, Phe70, Arg71, Ala82, Gln83, Ala86, Phe101, Arg105, Gln140, Asn141, Ile142, His158, Val221, Trp222, Phe223, and Tyr255 for SIRT5–sirtinol; Phe70, Arg71, Ala82, Phe101, Tyr102, Arg105, Gln140, Asn141, Ile142, His158, Val221, Trp222, Phe223, Gly224, Glu225, Asn226, Leu227, and Tyr255 for SIRT5–salermide; and Ala59, Thr69, Phe70, Arg71, Ala82, Ala86, Phe101, Tyr102, Arg105, Gln140, Asn141, Ile142, His158, Val220, Val221, Trp222, Phe223, Gly224, and Tyr255 for SIRT5–SirReal2. Among these residues, the major energy contributors could be Phe70, Arg71, Gln140, His158, Phe223, and Tyr255. These residues may regulate SIRT5 inhibitory activity to a greater extent (Supplementary Figure S10d).

In another SIRT isoform, i.e., SIRT6, the selected residues for the decomposition analysis were Lys13, Gly50, Ala51, Phe62, Arg63, Trp69, Gln111, Asn112, Val113, His131, Leu184, Asp185, Trp186, Asp188, Ser189, Leu190, Thr213, Ile217, Arg218, and Pro219 for SIRT6–sirtinol; Lys13, Gly50, Ala51, Phe62, Arg63, Trp69, Gln111, Asn112, Val113, His131, Leu184, Asp185, Trp186, Asp188, Leu190, Thr213, Ile217, Arg218, and Pro219 for SIRT6–salermide, and Gly50, Ala51, Gly52, Thr55, Asp61, Phe62, Arg63, Gly64, Gln111, Asn112, Val113, His131, Gly212, Thr213, Ser214, Leu215, Gln216, Ile217, Val237, Asn238, Leu239, Gln240, Gly254, Tyr255, and Val256 for SIRT6–SirReal2. Among all these residues, the common energy contributors were Asp61, Phe62, Arg63, His131, Trp186, Thr213, Ile217, and Arg218. However, the energy contributor residues in SIRT6–SirReal2 were slightly different from those in the other two (Supplementary Figure S10e).

The per-residue energy decomposition revealed different energetic fingerprints across different SIRT isoforms. SIRT1 and SIRT2 were mainly composed of hydrophobic and van der Waals-driven energetic fingerprints (aromatic and hydrophobic residues—Phe, Tyr, and Ile); SIRT3 exhibited mixed energetic fingerprint contributors (hydrophobic and polar residues); SIRT5 exhibited predominantly acidic substrate recognition electrostatic contributors, e.g., Arg71, Gln140, and His158; and SIRT6 showed an interface-stabilized hybrid energetic profile involving aromatic, polar, and charged residues.

## 4 Discussion

The NAD^+^-dependent class III histone deacetylase SIRT family of proteins plays a significant role in many cellular events, such as inflammation, metabolism, oxidative stress, and apoptosis. The SIRT family is regarded as a potential target in the pathophysiology of numerous systems and disorders, including diabetes, cancer, cardiovascular disease, respiratory disease, digestive system disorders, neurological disorders, and genitourinary disorders, due to its important biological functions (Wu et al., 2022). Despite numerous studies conducted to understand SIRT biology and its role in important cellular processes, many areas remain unexplored, such as the lack of SIRT-isoform selectivity and specificity, limited bioavailability, poor pharmacokinetic and pharmacodynamic properties, and the need for successful clinical trials for SIRT modulators. In this study, we focused on one of the research gaps—the lack of SIRT-isoform selectivity and specificity—through a computational approach.

Our study was executed in two aspects to address this gap: first, by studying the conformational changes in the structure of each SIRT isoform upon ligand binding, and second, by identifying the hot spot region or key residues specifically responsible for the regulation of SIRT inhibitory activity. The overall workflow of the study includes molecular docking of SIRT isoforms with standard ligand compounds that are experimentally proven to be SIRT isoform-selective or -non-selective. Using the molecular docking approach, we were able to filter out the top three standard ligands that exhibited a multi-SIRT isoform-positive outcome. Additionally, our in-house created Python script helped us identify key residues that showed interaction in one or the other form with at least 50% of the compounds (Supplementary Table S9). Furthermore, the selected top three ligands (sirtinol, salermide, and SirReal2) were considered for longer MDS to understand the conformational changes in C-alpha atoms and the binding spots of each isoform. We performed rigorous MDS trajectory analyses, including RMSD, RMSF, RG, SASA, PCA, DCCM, SSTP, NSR, cluster analysis, H-bond formation, H-bond percentage occupancy, TBFE, and decomposition analysis. Among all these analyses, many helped us assess both the stability and conformational dynamics of the system throughout the simulation period.

**FIGURE 15:**
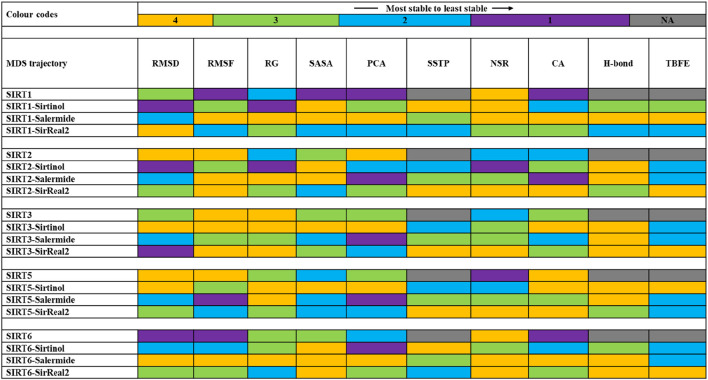
Color code comparison of all the trajectories among different analyses (note: to any equally stable systems, we have assigned a similar color to each other to show the closeness of the outcome. Abbreviations: RMSD, root mean square deviation; RMSF, root mean square fluctuation; RG, radius of gyration; SASA, solvent-accessible surface area; PCA, principal component analysis; DCCM, dynamic cross-correlation matrix; SSTP, secondary structure timeline plot; NSR, number of structured residues; CA, cluster analysis, H-bond; TBFE, total binding free energy; NA, not applicable.

A detailed outline of the structural biology outcomes of this study is shown in Figure 15. This color code representation provides a comprehensive view of the outcomes observed in our study. In a nutshell, in the SIRT1 protein, residues 272–295 and 344–403 were identified as the main flexible regions. These regions were probably responsible for ligand sensitivity and conformation dynamics. In the SIRT2 protein, apart from one flanking region (located away from the binding site), residues 180–230 and 290–314 were identified as the major regions responsible for conformational changes according to RMSF and SSTP analyses. For the SIRT3 target, the region responsible for the conformational dynamics was between residues 220 and 307, as indicated by RMSF and SSTP analyses. For the SIRT5 protein, no well-defined region was identified by RMSF analysis, but residues 120 to 190 could be considered a ligand-sensitive region according to SSTP. This observation was also slightly supported by RMSF analysis as residue 175 exhibited the highest peak. For the SIRT6 protein, residues 120–180 were commonly identified as responsible for the conformational dynamics of the SIRT6 target, as indicated by RMSF and SSTP analyses.

Apart from conformational dynamics, we were also able to assess the stabilization and destabilization of each SIRT isoform in holo forms. Based on the overall outcome of the SIRT1 protein, we concluded that the SIRT1 protein was more stabilized in the holo form than in the apo form. Among the three holo forms, SIRT1–salermide was the most stable complex, followed by SIRT1–sirtinol and SIRT1–SirReal2. The SIRT1–salermide complex outperformed eight out of ten analyses, as shown in Figure 15. For the SIRT2 protein, SIRT2–salermide performed better than SIRT2–SirReal2, followed by the SIRT1 apo form and SIRT1–sirtinol. However, SIRT1–SirReal2 was slightly deviated from the actual binding pocket. For the SIRT3 target, SIRT3–sirtinol and SIRT3–SirReal2 were evaluated to be equally stable as both of them performed similarly in the trajectory analyses. For the SIRT5 target, SIRT5–sirtinol was most stable, followed by SIRT5–salermide, and SIRT5–SirReal2 was equally stable. SIRT5–sirtinol outperformed seven out of ten analyses. For the SIRT6 protein, again, the SIRT6–salermide complex outperformed the other three systems in eight out of ten analyses. In a nutshell, salermide performed well on SIRT1, SIRT2, and SIRT6 (primarily nuclear SIRTs and cytoplasmic SIRT), whereas sirtinol performed well on SIRT3 and SIRT5 (mitochondrial SIRTs). This computational approach provides an extensive investigation with a reliable conclusion; however, this needs to be validated with *in vitro* experiments. The experimental proof will allow us to cross-check the prediction to identify SIRT-isoform-selective compounds.

As the second goal of the study, we have also identified the key residues or binding hot spots that may be responsible for the inhibitory activities of each SIRT isoform, as provided in Supplementary Table S9. We have consolidated the key residues using three different analyses, namely, molecular docking, H-bond percentage occupancy, and per-residue energy contribution (decomposition analysis). More priority was given to H-bond percentage occupancy and decomposition analysis as both belong to the dynamic environment. The SIRT1-isoform-selective key residues were identified as Ala262, Phe273, Tyr280, Pro293, Gln294, Phe297, Gln345, Ile347, His363, Ile411, Phe413, and Phe414. For SIRT2 targets, the selective key residues were observed as Phe96, Pro115, Glu116, Phe119, His187, Val233, Phe235, Leu239, Ser245, Ser271, and Lys275 in its catalytic pocket. For the SIRT3 protein, Phe157, Arg158, Phe180, Gln228, His248, Thr250, Ser253, Cys259, Gln260, Arg261, Pro262, Asp290, Val292, Phe294, Gly295, Leu296, Glu296, Pro297, Leu298, Pro299, Gln300, and Arg301 could be labeled as the selective residues for SIRT3 inhibition. For the SIRT5 protein, the selective key residues were Phe70, Arg71, Gln83, Tyr102, Arg105, Gln140, His158, Val221, Phe223, Glu225, and Tyr255. For the SIRT6 protein, Ala51, Asp61, Phe62, Arg63, Trp69, His131, Leu184, Asp185, Trp186, Gly212, Thr213, Ser214, Gln216, Ile217, Arg218, Asn238, and Leu239 were the key residues in selective inhibition.

Concisely, we concluded that the primary SIRT isoform-selective residues for SIRT1 were Phe273, Phe297, Tyr280, and His363; for SIRT2, they were Phe119, His187, Val233, Phe235, and Leu239; for SIRT3, they were Leu248, Glu296, and Arg301; for SIRT5, they were Phe70, Tyr102, Gln140, and His158; and for SIRT6, they were Asp61, Trp69, His131, Trp186, Ser214, Arg218, and Leu239.

Since our study was based on rigorous computational approaches, it was necessary to validate the results with experimental support. The experimentally determined 3D structure, along with the inhibitor molecule, was the best way to achieve this. We can analyze the crystallized structure, the binding pocket, and the key interaction of inhibitor molecules with the respective SIRT isoform. The experimentally proven structure (PDB ID: 4I5I), along with the co-crystallized inhibitor (4I5), exhibited key interactions with Ala262, Ser265, Ile270, Pro271, Asp272, Phe273, Ile279, Phe297, Ile316, Gln345, Asn346, Ile347, Asp348, His363, Val412, Ile411, and Phe413 residues (Zhao et al., 2013). Another co-crystallized SIRT1 structure (PDB ID: 4ZZI) contained both the inhibitor and the activator in the catalytic domain. The inhibitor molecule formed H-bonds with residues Phe273, Ile347, Asp348, His363, and Val412 (Dai et al., 2015), and the remaining interactions were also similar to the identified key residues in our study. SIRT2 had complete 3D structures, but these were not available in inhibitor-bound form. Disch et al. (2013) demonstrated that synthetic compounds 11c and 43 experimentally inhibited SIRT1, SIRT2, and SIRT3, and these compounds also exhibited interactions with Phe273, Tyr280, Phe297, His363, Phe413, and Phe414 of the SIRT1 protein and Phe157, Tyr165, Phe180, His248, Phe293, and Phe294 of the SIRT3 target. Most of the identified selective residues for the SIRT1 protein were consistent with the literature (Sinha et al., 2019; Ullah et al., 2023; Sharma and Muniyan, 2024).

Similarly, a crystallographic structure of SIRT3 (PDB ID: 4B3V) with the inhibitor EX-527 showed potential interactions with residues Ala146, Ser149, Ile154, Pro155, Phe157, Leu164, Phe180, Leu199, Gln228, Asn229, Ile230, Asp231, Ile291, Val292, and Phe293 (Gertz et al., 2013). The crystallographic structure of the SIRT5 protein (PDB ID: 2NYR), which contains suramin, a well-known SIRT inhibitor, formed H-bonds with Phe70, Tyr102, Arg105, and Tyr255 (Schuetz et al., 2007). The binding pocket of SIRT5 with the suramin inhibitor in the experimentally determined structure contained the same selective residues as suggested in our study. The crystallographic structure of the SIRT6 protein (PDB ID: 3K35) contains the ADP-ribose (ADPR) moiety of NAD^+^. However, ADPR is not directly involved in the inhibitory activity of the SIRT protein, but it can be considered for identifying the binding pocket of the SIRT6 protein. The ADPR molecule in the binding pocket of SIRT6 formed H-bonds with Ala51, Thr55, Phe62, Trp69, Gln111, His131, Thr213, Ser214, Asn238, Gln240, and Val256, along with other interactions. These interactions were in strong agreement with the outcome of SIRT6-selective residues (Pan et al., 2011). The crystallographic structures of different SIRT isoforms supported and validated our results.

A study conducted by Parenti et al. (2015) was based on the alignment of SIRT isoforms to identify selective binding hot spots in the catalytic pocket of SIRTs. This study listed a few key residues in Pocket A, Pocket B, Pocket C, and the substrate-binding regions that were predicted to be SIRT-isoform-selective. For the SIRT1 protein that was suggested to be selective, there were Val266, Arg466, Glu467, Asp481, and Cys482 in pocket A; Phe273 in pocket B; Ile279, Phe297, Phe312, Ala313, Ile316, and Ile347 in pocket C; and Pro293, Gln294, and Arg446 in the substrate binding pocket. For the SIRT2 protein, the selective residues were Thr89, Lys287, Glu288, Glu323, and Cys324 in pocket A; Phe96 in pocket B; Leu103, Phe119, Leu134, Leu183, and Ile169 in pocket C; and Pro115, Glu116, and Gln267 in the substrate binding pocket. For the SIRT3 protein, the selective residues were predicted as Thr150, Arg345, Asp346, Asp365, and Val366 in pocket A; Phe157 in pocket B; Leu164, Phe180, Leu195, Leu199, and Ile230 in pocket C; and Pro176, Glu177, and Glu325 in the substrate binding pocket. For the SIRT5 target, the selective residues were shown as Ala63, Thr276, Glu277, Pro292, and Cys293 in pocket A; Phe70 in pocket B; Gly75, Ala86, Phe101, Arg105, Met109, and Ile142 in pocket C; and Ala82, Gln83, and Tyr255 in the substrate binding pocket. For the SIRT6 protein, the selective residues were known as Thr57, Leu241, Gln242, Tyr257, and Val285 in pocket A; Phe64 in pocket B; Val70, Phe82, Phe86, and Val115 in pocket C; and Arg220 in the substrate binding pocket. For the SIRT7 protein, Thr112, Leu298, Gln299, Lys314, and Cys315 in pocket A; Tyr119 in pocket B; Val125, Ser136, Leu140, and Cys169 in pocket C; and Leu274 and Lys275 in the substrate binding pocket (Parenti et al., 2015). In this study, we focused on the entire structure of the protein, specifically in four regions, as mentioned earlier. Although our study focused primarily on the catalytic binding site of each SIRT isoform, we were able to identify a few similar residues that were predicted to be isoform-selective in both studies, mainly from the substrate-binding region. The common residues identified in our study and that of Parenti et al. (2015) were Phe273, Phe297, Ile347, Pro293, and Gln294 in SIRT1; Phe96, Phe119, and Glu116 in SIRT2; Phe157 and Phe180 in SIRT3; and Phe70 and Tyr255 in SIRT5. Additionally, catalytic histidine amino acids reported in the same study—His363, His187, His248, His158, and His131 for SIRT1, SIRT2, SIRT3, SIRT5, and SIRT6, respectively—were also identified as selective residues regulating SIRT isoform-predictive inhibitory activity. Therefore, the validity and authenticity of our outcomes were well-supported.

Another aspect to validate our study outcomes is based on the mutation in SIRT isoforms. Mutation of selective residues into other residues may provide broader insights to authenticate the outcomes. In one mutational study, Ser265, Gln345, Asn346, Ile347, Asp348, His363, and Phe414 key residues were reported to interact predominantly with the substrate and NAD^+^ cofactor. Furthermore, they have also investigated several mutations of these residues and experimentally compared the wild-type and mutant forms (Davenport et al., 2014). Another study investigated mutations at His187Ala and Tyr114Ala to study the dimer formation of hSIRT2 using biophysical experiments (Suzuki et al., 2024). Conversely, a point mutation at Val208Ile led to the loss of functional activity of the SIRT3 protein (Chen et al., 2013). Other mutations for the SIRT3 protein are yet to be explored. A study specific to the SIRT5 protein focused on mutating the Gln140, Asn141, and His158 residues. After comparing wild and mutant types using the Michaelis–Menten analysis and X-ray crystallography, they concluded that Asn141 was significantly responsible for the desuccinylation reaction (Yokoyama et al., 2024). Kugel et al. (2015) studied various mutations, such as Asp25Asn, Glu36Val, Asn46Ser, Asp63Tyr, Ala89Ser, Asp116Asn, Glu260Ter, Thr263Pro, and Pro274Leu, in the SIRT6 protein for cancer disease. Here, they concluded that Asp63Tyr greatly affected NAD^+^ binding, whereas Asp25Asn, Glu36Val, Ala89Ser, Thr263Pro, and Pro274Leu mutations were not directly responsible for binding the acetylated substrate or NAD^+^ in the SIRT6 protein.

These SIRT-based mutations have been described with different diseases. The mutation type may change as soon as we begin studying another disease. We have mentioned the major mutational studies on SIRT1, SIRT2, SIRT3, SIRT5, and SIRT6 proteins. Interestingly, most of these studies supported our identified SIRT isoform-selective residues to a greater extent. Therefore, the study’s outcomes are again validated from a different perspective. Furthermore, site-directed mutagenesis, enzyme inhibition assays for both wild-type and mutant forms using standard SIRT inhibitors, and structural analysis using cryo-EM and X-ray crystallography are suggested as future scopes of this study. A computational investigation of the wild and mutant forms of all possible combinations produced in our study, followed by *in vitro* evaluation, may also be conducted to understand the SIRT isoform-selective key residues. In brief, based on predictions and approximations, our study provides a foundation for developing an SIRT-isoform-selective modulator and outlines a predictive path to a successful, clinically approved SIRT inhibitor.

## 5 Conclusion

The extensive involvement of SIRTs in various pathological states has stimulated considerable interest in the design of SIRT isoform-specific inhibitors. This study attempts to address the gap of “lack of SIRT isoform-selectivity and specificity” by using advanced computational tools such as molecular docking and extended simulations with extensive analyses. As an outcome of this study, we gained insights into how an SIRT isoforms change upon standard ligand binding in a dynamic environment. We have also identified SIRT isoform-selective key residues in the catalytic binding pocket. The primary SIRT isoform-selective residues for SIRT1 were Phe273, Phe297, Tyr280, and His363; for SIRT2, they were Phe119, His187, Val233, Phe235, and Leu239; for SIRT3, they were Leu248, Glu296, and Arg301; for SIRT5, they were Phe70, Tyr102, Gln140, and His158; and for SIRT6, they were Asp61, Trp69, His131, Trp186, Ser214, Arg218, and Leu239. This study provides a basis for understanding the development of conformational dynamics in the SIRT family upon ligand binding and identifying SIRT isoform-selective compounds for successful clinical trials.

## Data Availability

The data produced in the study have been incorporated into the manuscript and the supplementary file. The codes and Python scripts used in our study have been provided on our publicly accessible GitHub repository: https://github.com/deepsharma26/SIRT-isoform_selective-.git.

## References

[B1] Abraham M. J. Murtola T. Schulz R. Páll S. Smith J. C. Hess B. (2015). Gromacs: high performance molecular simulations through multi-level parallelism from laptops to supercomputers. SoftwareX 1–2, 19–25. 10.1016/j.softx.2015.06.001 1

[B2] Bell I P. J. Muniyan R. (2025). Targeting the quorum sensing network in acinetobacter baumannii: a dual target structure-based approach for the development of novel antimicrobials. Comput. Biol. Med. 187, 109828. 10.1016/j.compbiomed.2025.109828 39938338

[B3] Broussy S. Laaroussi H. Vidal M. (2020). Biochemical mechanism and biological effects of the inhibition of silent information regulator 1 (SIRT1) by EX-527 (SEN0014196 or selisistat). J. Enzyme Inhib. Med. Chem. 35, 1124–1136. 10.1080/14756366.2020.1758691 32366137 PMC7241506

[B4] Chakraborty S. Levenson A. S. Biswas P. K. (2013). Structural insights into Resveratrol’s antagonist and partial agonist actions on estrogen receptor alpha. BMC Struct. Biol. 13, 27. 10.1186/1472-6807-13-27 24160181 PMC4015837

[B5] Chen I. C. Chiang W. F. Liu S. Y. Chen P. F. Chiang H. C. (2013). Role of SIRT3 in the regulation of redox balance during oral carcinogenesis. Mol. Cancer 12, 68. 10.1186/1476-4598-12-68 23800187 PMC3694519

[B6] Curry A. M. White D. S. Donu D. Cen Y. (2021). Human sirtuin regulators: the “success” stories. Front. Physiol. 12, 752117. 10.3389/fphys.2021.752117 34744791 PMC8568457

[B7] Dai H. Case A. W. Riera T. V. Considine T. Lee J. E. Hamuro Y. (2015). Crystallographic structure of a small molecule SIRT1 activator-enzyme complex. Nat. Commun. 6, 7645. 10.1038/ncomms8645 26134520 PMC4506539

[B8] Dang W. (2014). The controversial world of sirtuins. Drug Discov. Today Technol. 12, e9–e17. 10.1016/j.ddtec.2012.08.003 25027380 PMC4101544

[B9] Davenport A. M. Huber F. M. Hoelz A. (2014). Structural and functional analysis of human SIRT1. J. Mol. Biol. 426, 526–541. 10.1016/j.jmb.2013.10.009 24120939 PMC4211926

[B10] Disch J. S. Evindar G. Chiu C. H. Blum C. A. Dai H. Jin L. (2013). Discovery of thieno[3,2-d ]pyrimidine-6-carboxamides as potent inhibitors of SIRT1, SIRT2, and SIRT3. J. Med. Chem. 56, 3666–3679. 10.1021/jm400204k 23570514

[B11] Finkel T. Deng C. X. Mostoslavsky R. (2009). Recent progress in the biology and physiology of sirtuins. Nature 460, 587–591. 10.1038/nature08197 19641587 PMC3727385

[B12] Finnin M. Donigian J. biology N. P.-N. (2001). Structure of the histone deacetylase SIRT2. Nat. Struct. Biol. 8, 621–625. 10.1038/89668 11427894

[B13] Fiorentino F. Mautone N. Menna M. D’Acunzo F. Mai A. Rotili D. (2022). Sirtuin modulators: past, present, and future perspectives. Future Med. Chem. 14, 915–939. 10.4155/FMC-2022-0031 35583203 PMC9185222

[B14] Frye R. A. (2000). Phylogenetic classification of prokaryotic and eukaryotic Sir2-like proteins. Biochem. Biophys. Res. Commun. 273, 793–798. 10.1006/BBRC.2000.3000 10873683

[B15] Gertz M. Fischer F. Nguyen G. T. T. Lakshminarasimhan M. Schutkowski M. Weyand M. (2013). Ex-527 inhibits sirtuins by exploiting their unique NAD+-Dependent deacetylation mechanism. Proc. Natl. Acad. Sci. U. S. A. 110, E2772–E2781. 10.1073/PNAS.1303628110/SUPPL_FILE/PNAS.201303628SI.PDF 23840057 PMC3725051

[B16] Gey C. Kyrylenko S. Hennig L. Nguyen L. H. D. Büttner A. Pham H. D. (2007). Phloroglucinol derivatives guttiferone G, aristoforin, and hyperforin: inhibitors of human sirtuins SIRT1 and SIRT2. Angew. Chem. - Int. Ed. 46, 5219–5222. 10.1002/anie.200605207 17516596

[B17] Gowers R. Linke M. Barnoud J. Reddy T. Melo M. Seyler S. (2016). “MDAnalysis: a python package for the rapid analysis of molecular dynamics simulations,” in Proceedings of the 15th python in science conference. 10.25080/majora-629e541a-00e

[B18] Harris C. R. Millman K. J. van der Walt S. J. Gommers R. Virtanen P. Cournapeau D. (2020). Array programming with NumPy. Nature 585, 357–362. 10.1038/s41586-020-2649-2 32939066 PMC7759461

[B19] Heltweg B. Gatbonton T. Schuler A. D. Posakony J. Li H. Goehle S. (2006). Antitumor activity of a small-molecule inhibitor of human silent information regulator 2 enzymes. Cancer Res. 66, 4368–4377. 10.1158/0008-5472.CAN-05-3617 16618762

[B20] Herranz D. Maraver A. Cañamero M. Gómez-López G. Inglada-Pérez L. Robledo M. (2013). SIRT1 promotes thyroid carcinogenesis driven by PTEN deficiency. Oncogene 32, 4052–4056. 10.1038/onc.2012.407 22986535

[B21] Humphrey W. Dalke A. Schulten K. (1996). VMD: visual molecular dynamics. J. Mol. Graph 14, 33–38. 10.1016/0263-7855(96)00018-5 8744570

[B22] Hunter J. D. (2007). Matplotlib: a 2D graphics environment. Comput. Sci. Eng. 9, 90–95. 10.1109/MCSE.2007.55

[B23] Jaghoori M. M. Bleijlevens B. Olabarriaga S. D. (2016). 1001 ways to run AutoDock vina for virtual screening. J. Comput. Aided Mol. Des. 30, 237–249. 10.1007/s10822-016-9900-9 26897747 PMC4801993

[B24] Jain V. V. Anabala M. Sharma D. Muniyan R. (2025). Machine learning driven identification of therapeutic phytochemicals targeting hepatocellular carcinoma. Comput. Biol. Chem. 119, 108608. 10.1016/j.compbiolchem.2025.108608 40763563

[B25] Jena V. Naidu B. A. Sharma D. Bell P. J. Muniyan R. (2025). Computational discovery of novel SIRT4 inhibitors for cardiac hypertrophy treatment. Mitochondrion 85, 102076. 10.1016/j.mito.2025.102076 40834968

[B26] Karunakaran K. Muniyan R. (2023a). Identification of allosteric inhibitor against AKT1 through structure-based virtual screening. Mol. Divers 27, 2803–2822. 10.1007/s11030-022-10582-7 36522517

[B27] Karunakaran K. Muniyan R. (2023b). Integrating machine learning and high throughput screening for the discovery of allosteric AKT1 inhibitors. J. Biomol. Struct. Dyn. 43, 1893–1914. 10.1080/07391102.2023.2293265 38095558

[B28] Kugel S. Feldman J. L. Klein M. A. Silberman D. M. Sebastián C. Mermel C. (2015). Identification of and molecular basis for SIRT6 loss-of-function point mutations in cancer. Cell Rep. 13, 479–488. 10.1016/j.celrep.2015.09.022 26456828 PMC4618237

[B29] Lancelot J. Caby S. Dubois-Abdesselem F. Vanderstraete M. Trolet J. Oliveira G. (2013). Schistosoma mansoni sirtuins: characterization and potential as chemotherapeutic targets. PLoS Negl. Trop. Dis. 7, e2428. 10.1371/journal.pntd.0002428 24069483 PMC3772001

[B30] Mai A. Massa S. Lavu S. Pezzi R. Simeoni S. Ragno R. (2005). Design, synthesis, and biological evaluation of sirtinol analogues as class III histone/protein deacetylase (sirtuin) inhibitors. J. Med. Chem. 48, 7789–7795. 10.1021/jm050100l 16302818

[B31] McCarthy A. R. Pirrie L. Hollick J. J. Ronseaux S. Campbell J. Higgins M. (2012). Synthesis and biological characterisation of sirtuin inhibitors based on the tenovins. Bioorg Med. Chem. 20, 1779–1793. 10.1016/j.bmc.2012.01.001 22304848

[B32] McKinney W. (2010). “Data structures for statistical computing in python,” in Proceedings of the 9th python in science conference. 10.25080/majora-92bf1922-00a

[B33] Michaud-Agrawal N. Denning E. J. Woolf T. B. Beckstein O. (2011). MDAnalysis: a toolkit for the analysis of molecular dynamics simulations. J. Comput. Chem. 32, 2319–2327. 10.1002/jcc.21787 21500218 PMC3144279

[B34] Miller B. R. McGee T. D. Swails J. M. Homeyer N. Gohlke H. Roitberg A. E. (2012). MMPBSA.py: an efficient program for end-state free energy calculations. J. Chem. Theory Comput. 8, 3314–3321. 10.1021/ct300418h 26605738

[B35] Morris G. M. Huey R. Lindstrom W. Sanner M. F. Belew R. K. Goodsell D. S. (2009). AutoDock4 and AutoDockTools4: automated docking with selective receptor flexibility. AJ OlsonJournal Computational Chemistry, 2009•Wiley Online Libr. 16, 2785–2791. 10.1002/jcc.21256 19399780 PMC2760638

[B36] O’Boyle N. M. Banck M. James C. A. Morley C. Vandermeersch T. Hutchison G. R. (2011). Open babel: an open chemical toolbox. J. Cheminform 3, 33. 10.1186/1758-2946-3-33 21982300 PMC3198950

[B37] Pan P. W. Feldman J. L. Devries M. K. Dong A. Edwards A. M. Denu J. M. (2011). Structure and biochemical functions of SIRT6. J. Biol. Chem. 286, 14575–14587. 10.1074/jbc.M111.218990 21362626 PMC3077655

[B38] Pant R. Joshi A. Maiti P. Nand M. Pande V. Chandra S. (2020). Identification of potential mycolyltransferase Ag85C inhibitors of *Mycobacterium tuberculosis* H37Rv *via* virtual high throughput screening and binding free energy studies. J. Mol. Graph Model 98, 107584. 10.1016/j.jmgm.2020.107584 32200279

[B39] Parenti M. D. Bruzzone S. Nencioni A. Rio A. D. (2015). “Selectivity hot-spots of sirtuin catalytic cores. Mol. Biosyst. 11 2263–2272. 10.1039/C5MB00205B 26061123

[B40] Pasco M. Y. Rotili D. Altucci L. Farina F. Rouleau G. A. Mai A. (2010). Characterization of sirtuin inhibitors in nematodes expressing a muscular dystrophy protein reveals muscle cell and behavioral protection by specific sirtinol analogues. J. Med. Chem. 53, 1407–1411. 10.1021/jm9013345 20041717

[B41] Rotili D. Tarantino D. Nebbioso A. Paolini C. Huidobro C. Lara E. (2012). Discovery of salermide-related sirtuin inhibitors: binding mode studies and antiproliferative effects in cancer cells including cancer stem cells. J. Med. Chem. 55, 10937–10947. 10.1021/jm3011614 23189967

[B42] Rumpf T. Gerhardt S. Einsle O. Jung M. (2015). Seeding for sirtuins: microseed matrix seeding to obtain crystals of human Sirt3 and Sirt2 suitable for soaking. Acta Crystallogr. Section:F Struct. Biol. Commun. 71, 1498–1510. 10.1107/S2053230X15019986 26625292 PMC4666478

[B43] Schuetz A. Min J. Antoshenko T. Wang C. L. Allali-Hassani A. Dong A. (2007). Structural basis of inhibition of the human NAD+-dependent deacetylase SIRT5 by suramin. Structure 15, 377–389. 10.1016/j.str.2007.02.002 17355872

[B44] Sharma D. Muniyan R. (2024). Pharmacophore-guided *in-silico* discovery of SIRT1 inhibitors for targeted cancer therapy. Comput. Biol. Chem. 113, 108275. 10.1016/j.compbiolchem.2024.108275 39546858

[B45] Sharma A. Mahur P. Muthukumaran J. Singh A. K. Jain M. (2022). Shedding light on structure, function and regulation of human sirtuins: a comprehensive review. 3 Biotech. 2022 13 (1), 1–15. 10.1007/S13205-022-03455-1 36597461 PMC9805487

[B46] Sharma D. Panchaksaram M. Muniyan R. (2025). Advancements in understanding the role and mechanism of sirtuin family (SIRT1-7) in breast cancer management. Biochem. Pharmacol. 232, 116743. 10.1016/j.bcp.2025.116743 39761875

[B47] Sinha S. Patel S. Athar M. Vora J. Chhabria M. T. Jha P. C. (2019). Structure-based identification of novel sirtuin inhibitors against triple negative breast cancer: an *in silico* and *in vitro* study. Int. J. Biol. Macromol. 140, 454–468. 10.1016/j.ijbiomac.2019.08.061 31404596

[B48] Sinha S. Sharma S. Vora J. Shrivastava N. (2020). Emerging role of sirtuins in breast cancer metastasis and multidrug resistance: implication for novel therapeutic strategies targeting sirtuins. Pharmacol. Res. 158, 104880. 10.1016/j.phrs.2020.104880 32442721

[B49] Sundaresan N. R. Pillai V. B. Wolfgeher D. Samant S. Vasudevan P. Parekh V. (2011). The deacetylase SIRT1 promotes membrane localization and activation of Akt and PDK1 during tumorigenesis and cardiac hypertrophy. Sci. Signal 4, ra46. 10.1126/scisignal.2001465 21775285

[B50] Süssmuth S. D. Haider S. Landwehrmeyer G. B. Farmer R. Frost C. Tripepi G. (2015). An exploratory double-blind, randomized clinical trial with selisistat, a SirT1 inhibitor, in patients with Huntington’s disease. Br. J. Clin. Pharmacol. 79, 465–476. 10.1111/bcp.12512 25223731 PMC4345957

[B51] Suzuki T. Khan M. N. A. Sawada H. Imai E. Itoh Y. Yamatsuta K. (2012). Design, synthesis, and biological activity of a novel series of human sirtuin-2-selective inhibitors. J. Med. Chem. 55, 5760–5773. 10.1021/jm3002108 22642300

[B52] Suzuki N. Konuma T. Ikegami T. Akashi S. (2024). Biophysical insights into the dimer formation of human sirtuin 2. Protein Sci. 33, e4994. 10.1002/pro.4994 38647411 PMC11034489

[B53] Tian W. Chen C. Lei X. Zhao J. Liang J. (2018). CASTp 3.0: computed atlas of surface topography of proteins. Nucleic Acids Res. 46, W363–W367. 10.1093/nar/gky473 29860391 PMC6031066

[B54] Ullah A. Waqas M. Halim S. A. Daud M. Jan A. Khan A. (2023). Sirtuin 1 inhibition: a promising avenue to suppress cancer progression through small inhibitors design. J. Biomol. Struct. Dyn. 42, 9825–9841. 10.1080/07391102.2023.2252898 37661778

[B55] Valdés-Tresanco M. S. Valdés-Tresanco M. E. Valiente P. A. Moreno E. (2021). Gmx_MMPBSA: a new tool to perform end-state free energy calculations with GROMACS. J. Chem. Theory Comput. 17, 6281–6291. 10.1021/acs.jctc.1c00645 34586825

[B56] Vemula D. Jayasurya P. Sushmitha V. Kumar Y. N. Bhandari V. (2023). CADD, AI and ML in drug discovery: a comprehensive review. Eur. J. Pharm. Sci. 181, 106324. 10.1016/j.ejps.2022.106324 36347444

[B57] Wang Z. Yuan H. Roth M. Stark J. M. Bhatia R. Chen W. Y. (2013). SIRT1 deacetylase promotes acquisition of genetic mutations for drug resistance in CML cells. Oncogene 32, 589–598. 10.1038/onc.2012.83 22410779 PMC3376246

[B58] Waskom M. (2021). Seaborn: statistical data visualization. J. Open Source Softw. 6, 3021. 10.21105/joss.03021

[B59] Wu Q. J. Zhang T. N. Chen H. H. Yu X. F. Lv J.Le Liu Y. Y. (2022). The sirtuin family in health and disease. Signal Transduct. Target Ther. 7, 402. 10.1038/s41392-022-01257-8 36581622 PMC9797940

[B60] Yi J. Luo J. (2010). SIRT1 and p53, effect on cancer, senescence and beyond. Biochim. Biophys. Acta 1804, 1684–1689. 10.1016/J.BBAPAP.2010.05.002 20471503 PMC2989880

[B61] Yokoyama T. Takayama Y. Mizuguchi M. Nabeshima Y. Kusaka K. (2024). SIRT5 mutants reveal the role of conserved asparagine and glutamine residues in the NAD+-Binding pocket. FEBS Lett. 598, 2269–2280. 10.1002/1873-3468.14961 39031546

[B62] Yu H. Dalby P. A. (2020). A beginner’s guide to molecular dynamics simulations and the identification of cross-correlation networks for enzyme engineering. Methods Enzym. 643, 15–49. 10.1016/bs.mie.2020.04.020 32896280

[B63] Yuan H. Marmorstein R. (2012). Structural basis for sirtuin activity and inhibition. J. Biol. Chem. 287, 42428–42435. 10.1074/JBC.R112.372300 23086949 PMC3522243

[B64] Yuan H. Wang Z. Li L. Zhang H. Modi H. Horne D. (2012). Activation of stress response gene SIRT1 by BCR-ABL promotes leukemogenesis. Blood 119, 1904–1914. 10.1182/blood-2011-06-361691 22207735 PMC3293644

[B65] Zhang Y. Au Q. Zhang M. Barber J. R. Ng S. C. Zhang B. (2009). Identification of a small molecule SIRT2 inhibitor with selective tumor cytotoxicity. Biochem. Biophys. Res. Commun. 386, 729–733. 10.1016/j.bbrc.2009.06.113 19559674

[B66] Zhang Q. Zeng S. X. Zhang Y. Zhang Y. Ding D. Ye Q. (2012). A small molecule inauhzin inhibits SIRT1 activity and suppresses tumour growth through activation of p53. EMBO Mol. Med. 4, 298–312. 10.1002/emmm.201100211 22331558 PMC3376857

[B67] Zhao K. Harshaw R. Chai X. Marmorstein R. (2004). Structural basis for nicotinamide cleavage and ADP-Ribose transfer by NAD+-Dependent Sir2 histone/protein deacetylases. Proc. Natl. Acad. Sci. U. S. A. 101, 8563–8568. 10.1073/PNAS.0401057101/SUPPL_FILE/01057MOVIE1.MOV 15150415 PMC423234

[B68] Zhao X. Allison D. Condon B. Zhang F. Gheyi T. Zhang A. (2013). The 2.5 Å crystal structure of the SIRT1 catalytic domain bound to nicotinamide adenine dinucleotide (NAD +) and an indole (EX527 analogue) reveals a novel mechanism of histone deacetylase inhibition. J. Med. Chem. 56 963–969. 10.1021/JM301431Y/SUPPL_FILE/JM301431Y_SI_001 23311358

[B69] Zoete V. Cuendet M. A. Grosdidier A. Michielin O. (2011). SwissParam: a fast force field generation tool for small organic molecules. J. Comput. Chem. 32, 2359–2368. 10.1002/jcc.21816 21541964

