# Supplementary material for: Elucidating binding hot spots and structural stability in sirtuin family proteins for selective inhibitors: a computational approach
Source: Front Bioinform. 2026 Apr 10;6:1786061. doi: 10.3389/fbinf.2026.1786061 (PMC13106210; doi:10.3389/fbinf.2026.1786061)
Supplement: Supplementary file 1 [file Supplementaryfile1.docx]

**Elucidating binding hotspots and structural stability in sirtuin family proteins for selective inhibitors: A computational approach**

Deepak Sharma and Rajiniraja Muniyan*

School of Bio-Sciences and Technology, Vellore Institute of Technology, Vellore, Tamil Nadu 632014, India

Email: rajiniraja.m@vit.ac.in

**Table S1** Selection of PDB ID for different SIRT-isoforms and the binding site details.

| S. no. | SIRT isoform | PDB ID | Resolution (in Å) | Experimental method | Center x × center y × center z | Size x × size y × size z |
| --- | --- | --- | --- | --- | --- | --- |
|  | SIRT1 | 4ZZI | 2.73 | X-ray diffraction | 8.031 × 44.826 × -3.457 | 99 × 106 × 96 |
|  | SIRT2 | 1J8F | 1.70 | X-ray diffraction | 32.819 × 25.459 × 24.798 | 120 × 118 × 104 |
|  | SIRT3 | 5D7N | 1.83 | X-ray diffraction | 17.520 × -5.743 × 0.653 | 118 × 116 × 98 |
|  | SIRT5 | 2NYR | 2.06 | X-ray diffraction | 9.829 × -3.939 × 13.677 | 108 × 80 × 114 |
|  | SIRT6 | 3K35 | 2.00 | X-ray diffraction | 21.857 × 3.814 × 6.199 | 62 × 68 × 70 |

**Table S2** Standard-known compounds inhibiting different SIRT isoforms.

| **S. No.** | **Compound Name** | **PubChem ID** | **Chemical structures** |
| --- | --- | --- | --- |
| **1** | Sirtinol | 2827646 |  |
| **2** | EX527 (Selisistat) | 5113032 |  |
| **3** | Splitomicin | 5269 |  |
| **4** | Tenovin-1 | 1013376 |  |
| **5** | Tenovin-6 | 24772043 |  |
| **6** | AGK2 | 2130404 |  |
| **7** | Salermide | 135659046 |  |
| **8** | HR-73 | 6918866 |  |
| **9** | SirReal2 | 1096292 |  |
| **10** | Cambinol | 3246390 |  |
| **11** | Aristoforin | 98185391 |  |
| **12** | Nicotinamide | 936 |  |
| **13** | Inauhzin | 5494506 |  |
| **14** | AK-7 | 1328033 |  |
| **15** | AC93253 | 529988 |  |

**Table S3** Docking score of standard inhibitor molecules against different SIRT isoforms.

| S. No. | Compound Name | PubChem ID | SIRT1 | Interacting Residues | SIRT2 | Interacting Residues | SIRT3 | Interacting Residues | SIRT5 | Interacting Residues | SIRT6 | Interacting Residues |
| --- | --- | --- | --- | --- | --- | --- | --- | --- | --- | --- | --- | --- |
| 1 | Sirtinol | 2827646 | -11.1 | **H-bond:** Tyr280, **C-H bond:** His363, **VW:** Ile270, Asp272, Arg274, Gln345, Asn346, Ile411, Val412, Phe414, **Pi bond:** Ala262, Phe273, Phe297, Ile347, His363, Val445 | -9.7 | **C-H bond:** Tyr104, **VW:** Leu107. Leu112, Pro115, Leu134, Ile169, Ile232, Val233, Phe234, Phe235, **Pi bond:** Phe96, Tyr104, Ile118, Phe119, Leu138, His187 | -7.7 | **VW:** Phe157, Glu177, Val292, Phe293, Gly295, **Pi bond:** Phe180, His248, Phe294, Val324, **Other bond:** Arg158 | -8.6 | **VW**: Phe70, Ala86, Tyr102, Gln140, Asn141, His158, Val221, Trp222, **Pi bond and Alkyl bond**: Arg71, Ala82, Ile142, Val220, Phe223, Tyr255 | -10.1 | **H-bond**: His131, **Pi donor H-bond**: Gln111, **VW**: Gly50, Arg63, Asn112, Val113, Ile183, Trp186, Ile217, **Pi bond**: Ala51, Phe62, Trp69, His131, Leu184 |
| 2 | EX527 (Selisistat) | 5113032 | -7.7 | **H-bond:** Tyr280, **VW:** Ala262, Arg274, Gln345, Phe413, Val445, **Pi bond and Alkyl bond:** Phe273, Phe297, Ile347, His363, Val412, Phe414 | -7 | **H-bond:** Asn106, **VW:** Lys109, Tyr110, Lys136, Ile213, Val217, Thr218, **Pi bond:** Phe132, Ala133, Glu216 | -6.5 | **C-H bond:** Glu249, **VW:** Glu246, Thr250, Ser253, Thr255, Lys288, Ile291, Val292, Glu296, Arg301, **Pi bond:** Asp290, Pro299 | -7.4 | **H-bond:** Tyr102, Val221, **VW**: Phe70, Ph101, Ile142, His158, VAl220, Trp222, **Pi bond and Alkyl bond**: Ala82, Ala86, Phe223, Tyr255 | -7.6 | **H-bond**: Gln111, His131, **Pi donor H bond**: Gln111, **VW**: Gly50, Arg63, Trp69, Asn112, Val113, Thr213, Ser214, Ile217, **Pi bond**: Ala51, Phe62 |
| 3 | Splitomicin | 5269 | -9.6 | **VW:** Ile270, Asp272, Gln345, Asn346, Asp348, VAl445, **Pi bond:** Ala262, Phe273, Ile347, **Other bond:** Arg274 | -8.2 | **H-bond:** Arg201, **VW:** Gln180, Glu185, Gly188, Thr189, His194, Lys229, Val233, Glu237, Phe243, **Pi bond:** Asp231, Pro240 | -6.5 | **H-bond**: Lys288, **VW**: Gly249, Thr255, Ile291, Val292, Glu296, **Pi bond:** Asp290, Pro299 | -7.8 | **H-bond**: His158, Tyr255, **Pi-donor H-bond**: Tyr102, **VW**: Phe70, Phe101, Val221, Phe223, **Pi bond**: Ala82, Ala86, Ile142, His158, Val220 | -8.3 | **H-bond**: Arg63, **VW**: Ala51, Trp69, Gln111, His131, Thr213, Ile217, **Pi bond**: Phe62, Val113 |
| 4 | Tenovin-1 | 1013376 | -7.3 | **C-H bond:** Pro207, **VW:** Lys203, Leu206, Glu208, Thr209, Ile210, Phe273, Tyr280, Gln345, Ile347, ILe411, Val412, Val445, Arg446, **Pi bond:** Phe297, His363, Phe414 | -8.2 | **VW:** Ala85, Glu108, Leu112, Pro115, Phe119, Leu134, Leu138, Gln167, Asn168, **Pi bond and Alkyl bond:** Phe96, Tyr104, Leu107, Ile118, Ile169 | -7.8 | **H-bond:** Asp290, Glu296, **VW:** Leu182, Phe186, Glu246, Gly249, Thr250, Thr255, Thr257, Gln260, Arg301, **Pi bond and alkyl bond:** Asp290, Glu296, Pro299, **Other bond:** Lys288 | -8 | **H-bond**: Phe70, **VW**: Ser62, Val67, Thr69, Arg71, Ala82, Arg105, Asn141, Ile142, Asp143, His158, Leu161, Val221, Trp222, Gly224, Glu225, LEu227, **Pi bond**: Phe223, Tyr255 | -8.7 | **H-bond**: His131, Trp186, Asp188, **VW**: Gly50, Ala51, Arg63, Trp69, Gln111, Val113, Ile183, Asp185, Glu187, Ser189, Leu190, Thr213, Ser214, Arg218, Pro219, **Pi bond**: Phe62, Leu184, Ile217 |
| 5 | Tenovin-6 | 24772043 | -8.4 | **C-H bond:** His363, **VW:** Pro207, Ser265, Pro207, Asp272, Tyr280, Phe297, Gln345, Asn346, Asp348, Leu418, Pro447, **Pi bond and Alkyl bond:** Ala262, Ile270, Phe273, Ile347, His363, Phe414, Val445, Arg446 | -7.8 | **C-H bond:** Pro115, **VW:** Ala85, Leu107, Glu108, His111, Leu134, Leu138, Gln167, Asn168, **Pi bond and Alkyl bond:** Phe96, Tyr104, Leu112, Ile118, Phe119, Ile169 | -7 | **H-bond**: Glu296, **VW:** Phe186, Glu246, Gly249, Thr250, Thr255, Thr257, Gln260, Lys288, Arg301, **Pi bond and Alkyl bond**: Leu182, Asp290, Pro299, **Other bond**: Asp290 | -7.3 | **H-bond**: Glu225, **C-H bond**: Arg105, **VW:** Ala82, Ala86, Tyr102, Ile142, Val220, Val221, Trp222, Phe223, Gly224, Leu227, **Pi bond**: Phe70, His158, Tyr255, **Other bond**: Tyr255 | -9.5 | **H-bond**: Arg63, Gln111, Asn112, **C-H bond**: Ser214, Asn238 Val256, **VW**: Gly52, Thr55, Asp62, Gly64, Trp69, Leu184, Asp185, Gly212, Thr213, Gln240, Gly254, Tyr255, Val256, **Pi bond and Alkyl bond**: Ala51, Phe62, Arg63, His131, Trp186, Ile217, Leu239, **Other bond**: Arg63 |
| 6 | AGK2 | 2130404 | -10.5 | **VW:** Ala262, Phe297, Tyr280, Gln345, Asn346, Ile411, Val412, Phe413, Phe414, Val445, **Pi bond and Alkyl bond:** Phe273, Ile347, His363, Leu418, Arg446 | -8.7 | **VW:** Phe96, Glu108, Pro115, Leu134, Gln167, Ile232, **Pi bond and Alkyl bond**: Tyr104, Leu107, Leu112, Ile118, Phe119, Ile169, His18 | -8.9 | **C-H bond**: Leu211, Thr255, **VW**: Arg235, Val245, Thr250, Ser253, Thr257, Asp290, Ile291, Pro297, Pro299, Gln300, **Pi bond and Alkyl bond**: Glu246, Lys288, Val292, Glu296, Arg301 | -7.4 | **H-bond**: Arg217, **VW:** Pro88, Phe91, Gly159, Val220, Trp222, Asn226, Asp228, Pro229, **Pi bond and Alkyl bond**: Arg165, Thr167, Val221, His219, Glu225 | -9.6 | **VW:** Lys13, Gly50, Trp69, Gln111, Asn112, Val113, His131, Leu184, Asp185, Trp186, Leu190, Thr213, Ser214, Pro219, **Pi bond**: Ala51, Phe62, Arg63, Ile217 |
| 7 | Salermide | 135659046 | -11.6 | **H-bond:** His363, VAl412, **C-H bond:** His363, **VW:** Ile270, Asp272, Arg274, Gln345, Asn346, Ile411, Phe414, Leu418, **Pi bond**: Ala262, Phe273, Phe297, Ile347, His363, VAl445 | -9.3 | **VW:** Pro99, Glu108, Ile169, **Pi bond:** Phe96, Arg97, Tyr104, Leu107, Leu112, Ile118, Phe119, Leu134, Leu138 | -10 | **H-bond:** Lys288, Pro289, **VW:** Arg235, Leu244, Val245, Glu246, Gly249, Thr250, Thr255, Thr257, Gln260, Ile291, Gln300, **Pi bond and Alkyl bond**: Lys288, Asp290, Val292, Glu296, Pro299, Arg301 | -9.7 | **H-bond**: Gln140, Tyr255, **VW**: Ala59, Ph70, Phe101, Arg105, Asn141, Val221, Trp222, Phe223, Gly224, Glu225, Asn226, **Pi bond**: Ile142, His158, Leu227, Tyr255 | -9.2 | **VW**: Lys13, Arg63, Trp69, Gln111, Val113, Asp185, Trp186, Leu190, Pro219, **Pi bond**: Phe62, His131, Leu184, Ile217, Arg218, |
| 8 | HR-73 | 6918866 | -7 | **VW:** His363, Phe413, Phe414, Gly415, Asn417, His423, Arg446, **Pi bond:** Val412, Leu418 | -9.3 | **VW:** Pro115, Leu134, **Pi bond and Alkyl bond**: Phe96, Tyr104, Leu107, Leu112, Ile118, Phe119, Leu138, Ile169 | -7.5 | **VW**: Thr255, Thr257, Asp290, Val292, Leu298, Pro299, **Pi bond**: Phe186, Lys288, Glu296, Pro297 | -8.6 | **VW**: Arg71, Phe101, Tyr102, Ile142, Trp222, Glu225, **Pi bond and Alkyl bond**: Phe70, Ala82, Ala86, Val221, Phe223, Leu227, Tyr255, **Other bond**: Phe70 | -8.6 | **VW:** Lys13, Trp69, His131, Leu184, Asp185, Leu190, Arg218, **Pi bond**: Trp186, Ile217 |
| 9 | SirReal2 | 1096292 | -9.2 | **VW:** Thr200, Lys203, Phe273, Tyr280, Asp292, Gln294, Ile411, Val412, Phe413, **Pi bond and Alkyl bond:** Phe297, Pro293, Ile347, His363, Phe414, Val445 | -9.2 | **H-bond:** Arg201, Ser238, **C-H bond**: Asp231, **VW:** Arg174, Gln180, Glu185, Gly188, Thr189, Lys229, Val233, Leu239, Ala241, **Pi bond:** Asp231, Glu237, Pro240, Phe243, **Other bond:** Ser238 | -9.1 | **VW:** Glu246, Gly249, Thr250, Thr255, Thr257, Asp290, Val292, Pro297, Gln300, **Pi bond and Alkyl bond**: Leu182, Phe186, Lys288, Glu296, Pro299, Arg301 | -9.6 | **H-bond**: Arg105, **VW:** Ala59, Thr69, Tyr102, Gln140, Asn141, Val220, Val221, Trp222, Gly224, **Pi bond and Alkyl bond**: Phe70, Arg71, Phe101, Ile142, His158, Phe223, Tyr255 | -10.8 | **H-bond**: Ala51, Ser214, **C-H bond**: Gly64, **VW**: Glu20, Gly50, Gly52, Arg63, Trp69, Gln111, His131, Gly212, Thr213, Asn238, Gln240, **Pi bond and Alkyl bond**: Ala51, Asp61, Phe62, Pro65, Val113, Ile217, Leu239 |
| 10 | Cambinol | 3246390 | -6.8 | **H-bond:** Gly415, Glu416, Asn417, **C-H bond:** Phe414, **VW:** His363, Val412, Phe413, Val445, Arg446, Pro447, **Pi bond:** Leu418 | -7.5 | **VW:** Phe96, Arg97, Pro99, Glu108, Pro115, **Pi bond:** Tyr104, Leu107, Leu112, Ile118 | -6.2 | **VW:** Leu322, Glu323, Glu325, Ala328, Gly349, Ala352, **Pi bond:** Pro350, **Other bond**: Trp353 | -6.9 | **H-bond**: Gly224, Asn226, Leu227, **VW:** Val221, Trp222, Glu225, **Pi bond**: Phe223, Tyr255 | -9 | **H-bond**: Leu184, Asp185, **VW**: Arg63, Trp69, Gln111, His131, Gly156, Ile183, Ile217, **Pi bond**: Phe62, Val113, Met155, Trp186 |
| 11 | Aristoforin | 98185391 | -6.9 | **VW:** Asp204, Leu206, Glu208, Thr209, Phe297, His363, Phe413, Glu416, Asn417, Lys444, Val445, **Pi bond and Alkyl bond:** Lys203, Pro207, Ile210, Val412, Phe414, Leu418, Arg446 | -7.6 | **H-bond:** Ala85, **C-H bond:** His187, **VW:** Gly84, Gly86, Ser88, Pro94, Asp95, Arg97, Gln167, Asn168, Asp170, Gly261, Thr262, **Pi bond and Alkyl bond:** Ala85, Ile93, Phe96, Phe119, Ile169, His187, Ile232, Val266 | -6.7 | **H-bond**: Gln300, Arg301, **VW:** Glu246, Gly249, Thr250, Ser253, Thr255, Asp290, Ile291, Glu296, Pro297, **Alkyl bond**: Val245, Pro262, VAl292, Pro299, Arg301 | -5.8 | **H-bond**: Trp222, Glu225, **VW**: Gln83, Val221, Gly224, Asn226, Leu227, Tyr255, Met259, **Pi bond and Alkyl bond**: His158, Phe223, Ala258 | -7.4 | **H-bond**: Trp186, **VW**: Arg63, Val68, Glu72, Pro78, Met155, Ile183, Leu184, Asp185, **Pi bond and Alkyl bond**: Lys13, Phe62, Trp69, Phe80, Phe84, Val113, His131, Trp186, Ile217, |
| 12 | Nicotinamide | 936 | -5.5 | **H-bond:** Asn346, Ile347, Asp348, VW**:** Ile270, Asp272, Gln345, **Pi bond:** Ala262, Phe273, Ile347, **Other bond:** Ser365 | -4.6 | **H-bond:** Asp231, **VW:** Gly188, Thr189, Arg201, Lys229, Val233, Glu237, Phe243, **Pi bond:** Asp231, Pro240 | -4.8 | **H-bond**: His248, Val292, **C-H bond:** Val292, **VW:** Phe157, Arg158, Ile230, Ile292, Phe294, **Pi bond:** Phe180 | -4.5 | **H-bond**: His158, Val221, **VW**: Ala82, Ala86, Tyr102, Val220, Trp222, Phe223, Tyr255, **Pi bond**: His158 | -5.7 | **Pi donor H bond**: Gln111, **VW:** Gly50, Phe62, Arg63, Asn112, His131, Thr213, Leu215, Gln216, Ile217, **Pi bond**: Ala51, **Other bond**: Gln111, Ser214 |
| 13 | Inauhzin | 5494506 | -10.8 | **H-bond:** Gln345, **VW:** Lys203, Ile270, Pro271, Asp272, Arg274, Phe297, Asn346, Asp348, His363, Phe414, **Pi bond**: Ala262, Phe273, Ile347, Val445, **Other bond:** Tyr280 | -8.2 | **VW**: Asp95, Phe119, Leu134, Gln167, Asn168, Ile169, His187, Val266, **Pi bond**: Ala85, Phe96, Arg97, Leu138 | -7.9 | **Pi-donor H-bond**: Thr255, **VW**: Gly249, Thr250, Thr257, Gln260, Asp290, Val292, Pro297, Gln300, **Pi bond**: Lys288, Glu296, Pro299 | -9 | **H-bond:** Arg71, **VW:** Val67, Thr69, Phe70, Ala82, Gln83, Arg105, Gln140, Asn141, Ile142, His158, Phe223, **Pi bond and Alkyl bond**: Ala59, Arg71, Tyr255, **Other bond**: Arg71, | -9.4 | **VW**:Gly52, Asp60, Asp61, Gly64, Gly67, Lys79, Phe80, Asp81, Gly212, Asn238, Tyr255, **Pi bond**: Glu20, Thr55, Pro65, His66, Leu239, Val256 |
| 14 | AK-7 | 1328033 | -9.4 | **H-bond:** Val412, **VW:** Pro207, Phe297, Gln345, Ile411, Phe413, Leu418, Arg446, Pro447, **Pi bond and Alkyl bond:** Phe273, Ile347, His363, Phe414, Val445 | -8.7 | **VW:** Glu108, Pro115, Ile169, **Pi bond and Alkyl bond:** Phe96, Tyr104, Leu107, Leu112, Ile118, Phe119, Leu134, Leu138 | -5.8 | **C-H bond**: Pro326, **VW**: Arg158,Gly295, Glu296, Glu325, **Pi bond and Alkyl bond:** Phe180, His248, Val292, Phe294, Leu298, Val324 | -8.8 | **H-bond**: His158, Tyr255, **C-H bond**: Phe70, **VW**: Phe101, Tyr102, Ile142, VAl220, Val221, Trp222, Leu227, **Pi bond and Alkyl bond**: Ala82, Ala86, Phe223, Tyr255 | -9.8 | **H-bond**: Arg63, Trp69, Trp186, **Pi donor H bond**: Gln111 , **VW**: Lys13, Gly50, Asn112, His131, Ile183, Leu184, Asp185, Thr213, Ser214, **Pi bond and Alkyl bond**: Ala51, Phe62, Val113, Trp186, Ile217 |
| 15 | AC93253 | 529988 | -5.4 | **C-H bond:** Asn346, **VW:** Ala262, Ile270, Ile316, Gln345, Asp348, His363, Val412, **Pi bond and Alkyl bond**: Phe273, Phe297, Ile347, Ile411 | -4.6 | **H-bond**: Arg174, Gln180, **VW:** Glu185, Thr189, Thr192, His194, Arg201, Lys229, Asp231, Ile232, Val233, Glu237, **Alkyl bond**: Pro240 | -4.7 | **VW**: Pro155, Asp156, Arg158, Leu195, Leu199, Asn229, **Alkyl bond**: Phe157, Phe180, Ile230, His248, Phe294 | -4.1 | **H-bond**: Tyr102, **VW**: Arg105, Gln140, Val221, Trp222, Phe223, **Pi bond and Alkyl bond**: Phe70, Ala82, Ala86, Phe101, Ile158, His158, Val220 | -5.1 | **VW**: Gly50, Asp61, Gly64, Gln111, His131, Thr213, Ser214, **Pi bond and Alkyl bond**: Ala51, Phe62, Arg63, Trp69, Ile217 |

Note: All the docking scores have been considered in kcal/mol. Abbreviations: Conventional Hydrogen Bond (H-bond): , Carbon-Hydrogen bond (C-H bond): Pi-donor H-bond, Van der Waals (VW): , Pi bonds (Pi-sigma, Pi-Pi T-shaped, Pi-Pi stacked, Pi-alkyl, Alkyl, Pi-cation, Pi anion, Pi-sulphur, Amide-Pi-stacked, Pi-Lone pair ), Halogen bond, Other bonds (Unfavorable Bump, Unfavorable donner-acceptor, Unfavorable donner-donner, Unfavorable acceptor-acceptor, attractive charges, Sulphur -X, Salt bridge)

**Table S4** Average RMSD, RMSF, RG, SASA, and hydrogen bonds description of SIRT-isoform in apo state and top 3 standard ligand-bound states.

| S. No. | MDS trajectory | RMSD (nm) | RMSF (nm) | RG (nm) | SASA (nm^2^) | H- bonds |
| --- | --- | --- | --- | --- | --- | --- |
| 1 | SIRT1 | 0.3076 ± 0.110 | 0.2465 ± 0.151 | 2.1915 ± 0.035 | 181.43 ± 3.27 | - |
| 2 | SIRT1-Sirtinol | 0.3641 ± 0.065 | 0.1732 ± 0.084 | 2.2366 ± 0.029 | 176.73 ± 4.31 | 0-6 |
| 3 | SIRT1-Salermide | 0.3532 ± 0.040 | 0.1590 ± 0.087 | 2.1230 ± 0.021 | 176.79 ± 3.98 | 0-7 |
| 4 | SIRT1-SirReal2 | 0.2595 ± 0.036 | 0.1876 ± 0.084 | 2.1562 ± 0.025 | 179.47 ± 4.30 | 0-4 |
| 5 | SIRT2 | 0.6859 ± 0.069 | 0.2111 ± 0.179 | 2.2350 ± 0.038 | 169.11 ± 3.56 | - |
| 6 | SIRT2-Sirtinol | 0.9017 ± 0.142 | 0.2342 ± 0.229 | 2.2999 ± 0.018 | 165.76 ± 3.98 | 0-6 |
| 7 | SIRT2-Salermide | 0.8294 ± 0.193 | 0.2584 ± 0.279 | 2.2332 ± 0.035 | 166.88 ± 5.02 | 0-6 |
| 8 | SIRT2-SirReal2 | 0.7568 ± 0.114 | 0.2023 ± 0.184 | 2.3164 ± 0.020 | 169.45 ± 3.14 | 0-5 |
| 9 | SIRT3 | 0.1692 ± 0.022 | 0.1308 ± 0.060 | 1.9954 ± 0.013 | 134.43 ± 2.08 | - |
| 10 | SIRT3-Sirtinol | 0.1603 ± 0.022 | 0.1288 ± 0.058 | 1.9852 ± 0.011 | 133.07 ± 2.32 | 0-5 |
| 11 | SIRT3-Salermide | 0.1739 ± 0.022 | 0.1645 ± 0.110 | 2.0104 ± 0.014 | 137.12 ± 2.51 | 0-5 |
| 12 | SIRT3-SirReal2 | 0.2296 ± 0.034 | 0.1391 ± 0.054 | 1.9982 ± 0.014 | 134.76 ± 2.60 | 0-7 |
| 13 | SIRT5 | 0.1950 ± 0.025 | 0.1268 ± 0.058 | 2.0003 ± 0.014 | 131.02 ± 2.12 | - |
| 14 | SIRT5-Sirtinol | 0.2008 ± 0.024 | 0.1342 ± 0.055 | 1.9778 ± 0.013 | 127.60 ± 2.18 | 0-7 |
| 15 | SIRT5-Salermide | 0.2687 ± 0.040 | 0.1483 ± 0.068 | 1.9946 ± 0.016 | 131.50 ± 2.94 | 0-5 |
| 16 | SIRT5-SirReal2 | 0.2210 ± 0.025 | 0.1331 ± 0.062 | 2.0051 ± 0.013 | 128.54 ± 2.32 | 0-6 |
| 17 | SIRT6 | 0.2809 ± 0.092 | 0.1749 ± 0.147 | 1.9151 ±0.022 | 147.74 ± 4.44 | - |
| 18 | SIRT6-Sirtinol | 0.2607 ± 0.091 | 0.1817 ± 0.112 | 1.9154 ± 0.017 | 141.92 ± 4.25 | 0-6 |
| 19 | SIRT6-Salermide | 0.2083 ± 0.040 | 0.1512 ± 0.090 | 1.9011 ± 0.012 | 142.55 ± 3.31 | 0-5 |
| 20 | SIRT6-SirReal2 | 0.2687 ± 0.046 | 0.1659 ± 0.096 | 1.9231 ± 0.013 | 140.07 ± 4.12 | 0-9 |

**Table S5** Percentage area covered as correlated, anti-correlated, and neutral regions in DCCM plots.

| MDS trajectory | Percentage of correlated area | Percentage of anti-correlated area | Percentage of neutral area |
| --- | --- | --- | --- |
| SIRT1 | 53.96 | 41.11 | 4.92 |
| SIRT1-Sirtinol | 51.75 | 43.54 | 4.70 |
| SIRT1-Salermide | 50.97 | 44.38 | 4.64 |
| SIRT1-SirReal2 | 50.64 | 44.41 | 4.94 |
| SIRT2 | 53.82 | 41.61 | 4.56 |
| SIRT2-Sirtinol | 51.75 | 44.08 | 4.16 |
| SIRT2-Salermide | 52.89 | 42.70 | 4.40 |
| SIRT2-SirReal2 | 52.33 | 43.72 | 3.94 |
| SIRT3 | 50.52 | 45.27 | 4.20 |
| SIRT3-Sirtinol | 49.86 | 46.24 | 3.88 |
| SIRT3-Salermide | 50.33 | 44.79 | 4.86 |
| SIRT3-SirReal2 | 50.34 | 45.81 | 3.83 |
| SIRT5 | 49.31 | 47.09 | 3.58 |
| SIRT5-Sirtinol | 50.02 | 45.47 | 4.49 |
| SIRT5-Salermide | 49.12 | 45.81 | 5.06 |
| SIRT5-SirReal2 | 49.13 | 47.11 | 3.75 |
| SIRT6 | 51.41 | 43.78 | 4.79 |
| SIRT6-Sirtinol | 52.41 | 42.24 | 5.33 |
| SIRT6-Salermide | 51.22 | 43.83 | 4.93 |
| SIRT6-SirReal2 | 51.17 | 43.75 | 5.06 |

**Table S6** Change in total number of structured residues throughout the simulations for SIRT apo forms and holo forms

| System | Total Residues | Initial Structured | Average Structured | Std Dev | Change from Initial | Structured_% | Coil_% |
| --- | --- | --- | --- | --- | --- | --- | --- |
| SIRT1 | 335 | 260 | 259.12 | 4.09 | -0.88 | 77.35 | 22.65 |
| SIRT1-Sirtinol | 335 | 261 | 261.12 | 3.10 | 0.12 | 77.95 | 22.05 |
| SIRT1-Salermide | 335 | 260 | 260.08 | 4.81 | 0.08 | 77.63 | 22.37 |
| SIRT1-SirReal2 | 335 | 259 | 263.24 | 3.20 | 4.24 | 78.58 | 21.42 |
| SIRT2 | 312 | 232 | 238.01 | 4.80 | 6.01 | 76.29 | 23.71 |
| SIRT2-Sirtinol | 312 | 236 | 243.45 | 3.65 | 7.45 | 78.03 | 21.97 |
| SIRT2-Salermide | 312 | 235 | 240.13 | 3.50 | 5.13 | 76.97 | 23.03 |
| SIRT2-SirReal2 | 312 | 237 | 240.51 | 3.42 | 3.51 | 77.09 | 22.91 |
| SIRT3 | 271 | 218 | 213.19 | 3.92 | -4.81 | 78.67 | 21.33 |
| SIRT3-Sirtinol | 271 | 218 | 215.62 | 4.41 | -2.38 | 79.57 | 20.43 |
| SIRT3-Salermide | 271 | 218 | 215.62 | 4.41 | -2.38 | 79.57 | 20.43 |
| SIRT3-SirReal2 | 271 | 216 | 213.47 | 3.32 | -2.53 | 78.77 | 21.23 |
| SIRT5 | 259 | 203 | 198.04 | 2.44 | -4.96 | 76.46 | 23.54 |
| SIRT5-Sirtinol | 259 | 203 | 200.51 | 2.85 | -2.49 | 77.42 | 22.58 |
| SIRT5-Salermide | 259 | 201 | 199.55 | 2.41 | -1.45 | 77.04 | 22.96 |
| SIRT5-SirReal2 | 259 | 201 | 200.38 | 3.16 | -0.62 | 77.37 | 22.63 |
| SIRT6 | 274 | 187 | 187.83 | 3.45 | 0.83 | 68.55 | 31.45 |
| SIRT6-Sirtinol | 274 | 188 | 190.21 | 3.77 | 2.21 | 69.42 | 30.58 |
| SIRT6-Salermide | 274 | 187 | 187.84 | 3.71 | 0.84 | 68.56 | 31.44 |
| SIRT6-SirReal2 | 274 | 189 | 188.33 | 3.57 | -0.66 | 68.74 | 31.26 |

**Table S7** Cluster analysis of SIRT-isoform-based trajectories with RMSD cutoff 0.2 nm.

| S. No. | MDS trajectory | Range of RMSD (nm) | Average RMSD (nm) | Energy of the matrix | Total number of clusters found | Number of structures in the best clusters |
| --- | --- | --- | --- | --- | --- | --- |
| 1 | SIRT1 | 0.050353-0.899036 | 0.0838819 | 92.4605 | 21 | 6992 |
| 2 | SIRT1-Sirtinol | 0.0562564-0.679924 | 0.0435446 | 117.875 | 21 | 15197 |
| 3 | SIRT1-Salermide | 0.0523198-0.596657 | 0.0419409 | 108.601 | 8 | 16655 |
| 4 | SIRT1-SirReal2 | 0.0541965-0.567007 | 0.0419522 | 116.289 | 15 | 11160 |
| 5 | SIRT2 | 0.0571874-0.922198 | 0.0548841 | 129.689 | 52 | 6463 |
| 6 | SIRT2-Sirtinol | 0.0560881-1.3153 | 0.0838819 | 124.725 | 36 | 7608 |
| 7 | SIRT2-Salermide | 0.0544969-1.17185 | 0.0838819 | 123.544 | 52 | 4914 |
| 8 | SIRT2-SirReal2 | 0.0564298-1.0639 | 0.0437037 | 127.59 | 37 | 7739 |
| 9 | SIRT3 | 0.0484074-0.409654 | 0.0230033 | 87.7508 | 3 | 19956 |
| 10 | SIRT3-Sirtinol | 0.052116-0.341174 | 0.0213712 | 109.292 | 1 | 20001 |
| 11 | SIRT3-Salermide | 0.0527701-0.439772 | 0.0419409 | 113.26 | 5 | 18916 |
| 12 | SIRT3-SirReal2 | 0.0530989-0.433698 | 0.0258819 | 110.026 | 3 | 19549 |
| 13 | SIRT5 | 0.0462356-0.404205 | 0.0258607 | 85.629 | 3 | 19946 |
| 14 | SIRT5-Sirtinol | 0.0515502-0.365434 | 0.0241906 | 109.153 | 5 | 19925 |
| 15 | SIRT5-Salermide | 0.0523514-0.51659 | 0.0419409 | 108.683 | 3 | 18702 |
| 16 | SIRT5-SirReal2 | 0.0498085-0.385702 | 0.0260289 | 105.745 | 4 | 19957 |
| 17 | SIRT6 | 0.0475917-0.567183 | 0.0422434 | 79.6445 | 9 | 9381 |
| 18 | SIRT6-Sirtinol | 0.0501642-0.648556 | 0.0462568 | 95.4211 | 12 | 13488 |
| 19 | SIRT6-Salermide | 0.0505773-0.40106 | 0.0419409 | 94.0933 | 5 | 18881 |
| 20 | SIRT6-SirReal2 | 0.0488539-0.541069 | 0.0419416 | 93.7158 | 8 | 15123 |

**Table S8** MMPBSA calculations of SIRT-isoform with top 3 standard ligand molecules (ΔVDWAALS: Vander Waals contribution, ΔEEL: electrostatic energy, ΔEGB: Polar solvation-free energy, ΔESURF: non-polar solvation-free energy, ΔGGAS: Gas face free energy, ΔGSOLV: solvation energy, ΔTOTAL: total calculated free Binding energy, i.e., ΔGBinding (all units are reported in kcal/mol).

| Energy Component  (kcal/mol) | ΔVDWAALS | ΔEEL | ΔEGB | ΔESURF | ΔGGAS | ΔGSOLV | ΔTOTAL |
| --- | --- | --- | --- | --- | --- | --- | --- |
| SIRT1-Sirtinol | -46.27 ± 2.25 | -12.29 ± 4.36 | 39.16 ± 3.90 | -6.34 ± 0.21 | -58.56 ± 5.28 | 32.83 ± 3.78 | -25.74 ± 2.45 |
| SIRT1-Salermide | -47.08 ± 2.22 | -13.18 ± 2.56 | 39.42 ± 2.31 | -6.40 ± 0.21 | -60.26 ± 3.34 | 33.02 ± 2.23 | -27.24 ± 2.26 |
| SIRT1-SirReal2 | -40.61 ± 2.80 | -23.94 ± 5.28 | 44.70± 5.41 | -4.94 ± 0.32 | -64.54 ± 6.13 | 39.75 ± 5.28 | -24.79 ± 2.59 |
| SIRT2-Sirtinol | -40.94 ± 2.34 | -3.56 ± 2.41 | 27.91 ± 2.22 | -5.48 ± 0.24 | -44.50 ± 3.38 | 22.44 ± 2.15 | -22.06 ± 2.44 |
| SIRT2-Salermide | -39.00 ± 4.12 | -19.72 ± 10.50 | 38.94 ± 7.98 | -5.62 ± 0.57 | -58.72 ± 11.71 | 33.33 ± 7.61 | -25.39 ± 4.94 |
| SIRT2-SirReal2 | -39.32 ± 4.01 | -36.69 ± 9.93 | 50.87 ± 7.04 | -5.36 ± 0.43 | -76.00 ± 10.39 | 45.51 ± 7.00 | -30.49 ± 4.58 |
| SIRT3-Sirtinol | -38.97 ± 2.58 | -8.10 ± 3.56 | 31.71 ± 3.66 | -5.07 ± 0.30 | -47.07 ± 4.72 | 26.64 ± 3.50 | -20.43 ± 2.20 |
| SIRT3-Salermide | -32.66 ± 4.42 | -7.38 ± 3.65 | 25.58 ± 4.65 | -4.37 ± 0.52 | -40.05 ± 6.56 | 21.21 ± 4.30 | -18.84 ± 3.29 |
| SIRT3-SirReal2 | -48.91 ± 3.24 | -46.43 ± 6.68 | 60.04 ± 5.04 | -6.11 ± 0.17 | -95.34 ± 6.44 | 53.93 ± 5.05 | -41.41 ± 3.70 |
| SIRT5-Sirtinol | -50.67 ± 2.64 | -18.54 ± 4.73 | 33.12 ± 2.75 | -6.15 ± 0.19 | -69.21 ± 4.23 | 26.97 ± 2.75 | -42.24 ± 2.67 |
| SIRT5-Salermide | -41.35 ± 3.45 | -17.38 ± 3.69 | 36.36 ± 3.24 | -5.71 ± 0.53 | -58.73 ± 4.71 | 30.65 ± 3.18 | -28.08 ± 3.30 |
| SIRT5-SirReal2 | -38.86 ± 3.32 | -18.55 ± 5.60 | 35.59 ± 4.96 | -5.00 ± 0.42 | -57.41 ± 5.75 | 30.59 ± 4.99 | -26.83 ± 3.26 |
| SIRT6-Sirtinol | -40.07 ± 3.64 | -7.95 ± 7.65 | 26.91 ± 7.97 | -5.06 ± 0.39 | -48.02 ± 9.19 | 21.86 ± 7.82 | -26.17 ± 3.15 |
| SIRT6-Salermide | -33.63 ± 3.69 | -6.15 ± 4.47 | 24.39 ± 6.13 | -4.50 ± 0.48 | -39.78 ± 6.99 | 19.89 ± 5.80 | -19.89 ± 2.47 |
| SIRT6-SirReal2 | -58.35 ± 3.47 | -63.75 ± 5.09 | 70.76 ± 3.94 | -7.21 ± 0.28 | -122.10 ± 5.79 | 63.55 ± 3.91 | -58.55 ± 3.76 |

**Table S9** Overall comparison of key residues (binding hot spot) identified in different SIRT isoforms

| **Target** | **Common residues present in at least 50% of compounds among SIRT-isoforms and all standard ligands through molecular docking** | **Major residues present in the H-bond formation obtained by H-bond% occupancy** | **Major energy contributor residues obtained by decomposition analysis** |
| --- | --- | --- | --- |
| SIRT1 | Ala262, Ile270, Phe273, Tyr280, Phe297, Gln345, Asn346, Ile347, His363, Val412, Phe413, Phe414, Leu418, Val445, Arg446 | Ala262, Tyr280, Pro293, Gln294, Phe297, Gln345, His363 | Ala262, Phe273, Tyr280, Phe297, Ile347, His363, Ile411, Phe413, Phe414 |
| SIRT2 | Phe96, Tyr104, Leu107, Leu112, Pro115, Ile118, Phe119, Leu134, Leu138, Ile169 | Glu116, His187, Val233, Phe235, Leu239, Ser245, Ser271, Lys275 | Phe96, Pro115, Phe119, Phe235, Leu239 |
| SIRT3 | Glu246, Gly249, Thr250, Thr255, Thr257, Lys288, Asp290, Val292, Glu296, Pro299, Arg301 | Gln228, His248, Cys259, Arg261, Pro262, Asp290, Gly295, Leu296, Pro297, Leu298, Gln300, Arg301 | Phe157, Arg158, Phe180, Pro297, His248, Val292, Phe294, Leu298, Thr250, Ser253, Gln260, Glu296, Pro297, Pro299, Gln300, Arg301. |
| SIRT5 | Phe70, Ala82, Ala86, Tyr102, Ile142, His158, Val220, Val221, Trp222, Phe223, Glu225, Tyr255 | Phe70, Gln83, Tyr102, Arg105, Gln140, His158, Val221, Glu225, Tyr255 | Phe70, Arg71, Gln140, His158, Phe223, Tyr255 |
| SIRT6 | Gly50, Ala51, Phe62, Arg63, Trp69, Gln111, Asn112, Val113, His131, Leu184, Asp185, Trp186, Thr213, Ser214, Ile217 | Ala51, Asp61, Trp69, His131, Leu184, Asp185, Trp186, Gly212, Thr213, Ser214, Gln216, Arg218, Asn238, Leu239 | Asp61, Phe62, Arg63, His131, Trp186, Thr213, Ile217, Arg218 |


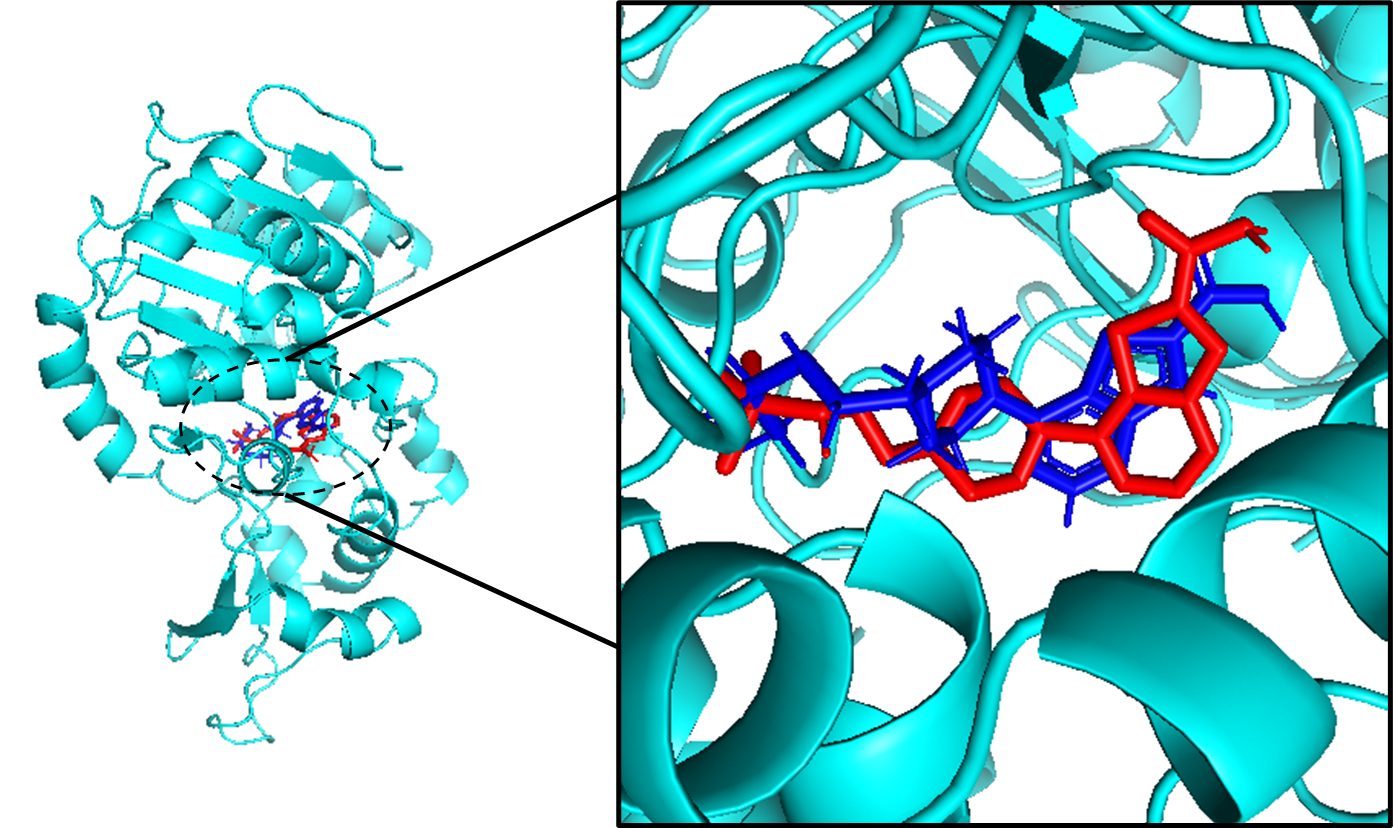


**Fig. S1** Superimposition of docked pose (red) with co-crystallized ligand (blue) to validate the docking procedure in the binding pocket of SIRT1 (RMSD =    2.339 Å)


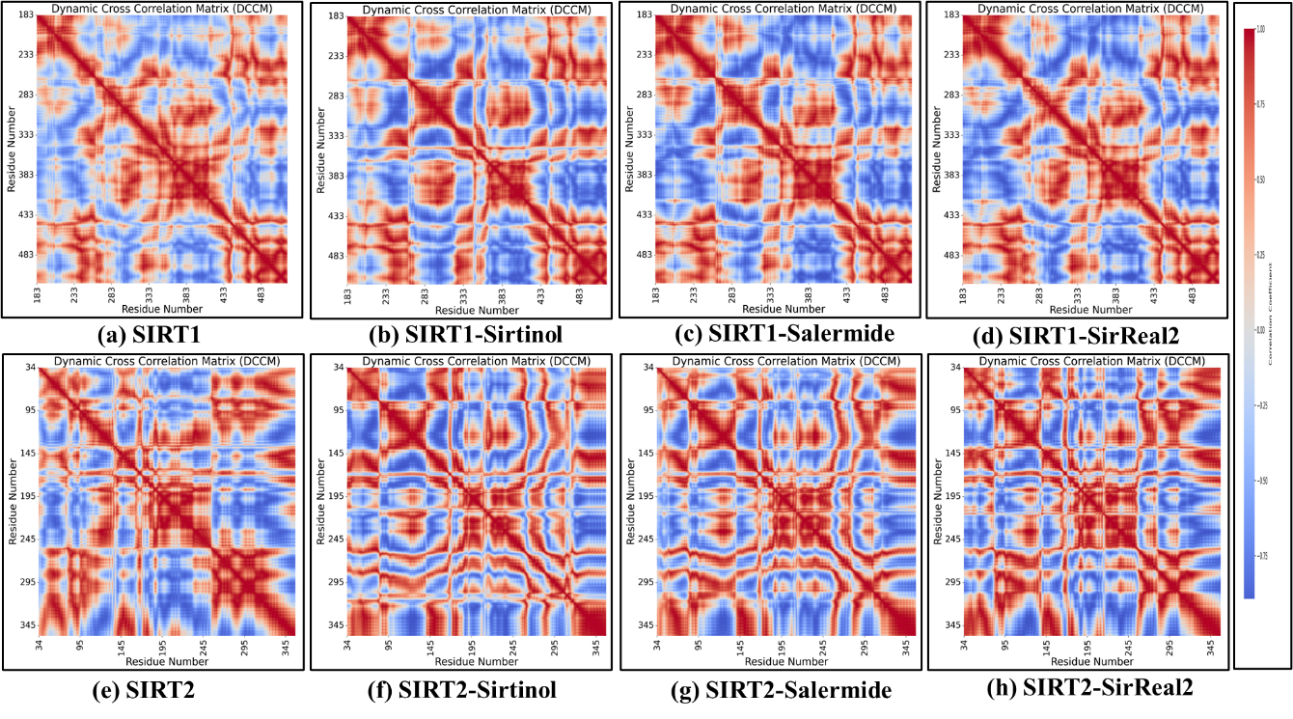


**Fig.** **S2** Representation of DCCM analysis showing the correlated and anticorrelated residues throughout the simulation for SIRT1 and SIRT2


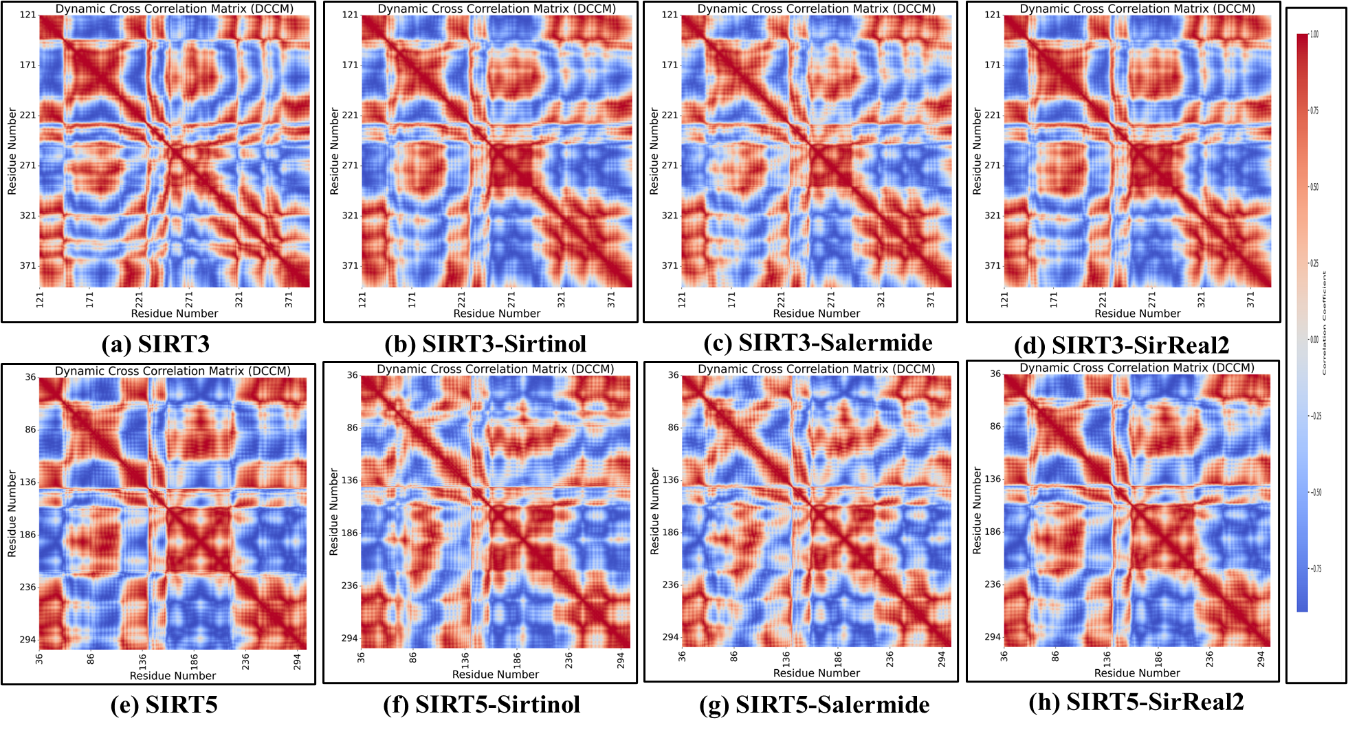


**Fig.** **S3** Representation of DCCM analysis showing the correlated and anticorrelated residues throughout the simulation for SIRT3 and SIRT5


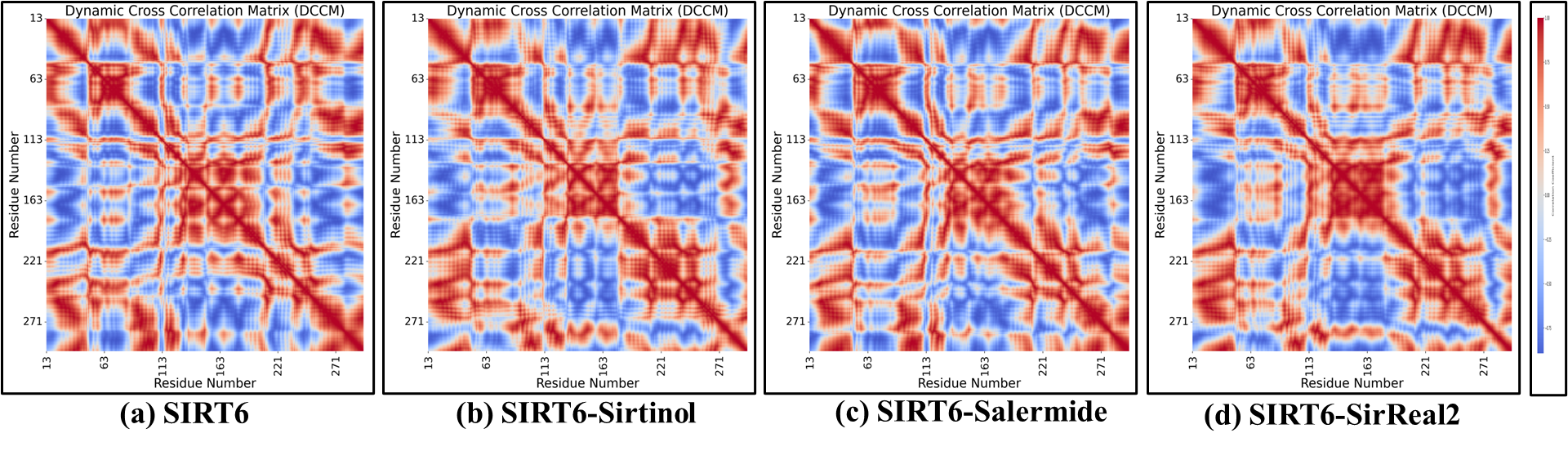


**Fig.** **S4** Representation of DCCM analysis showing the correlated and anticorrelated residues throughout the simulation for SIRT6


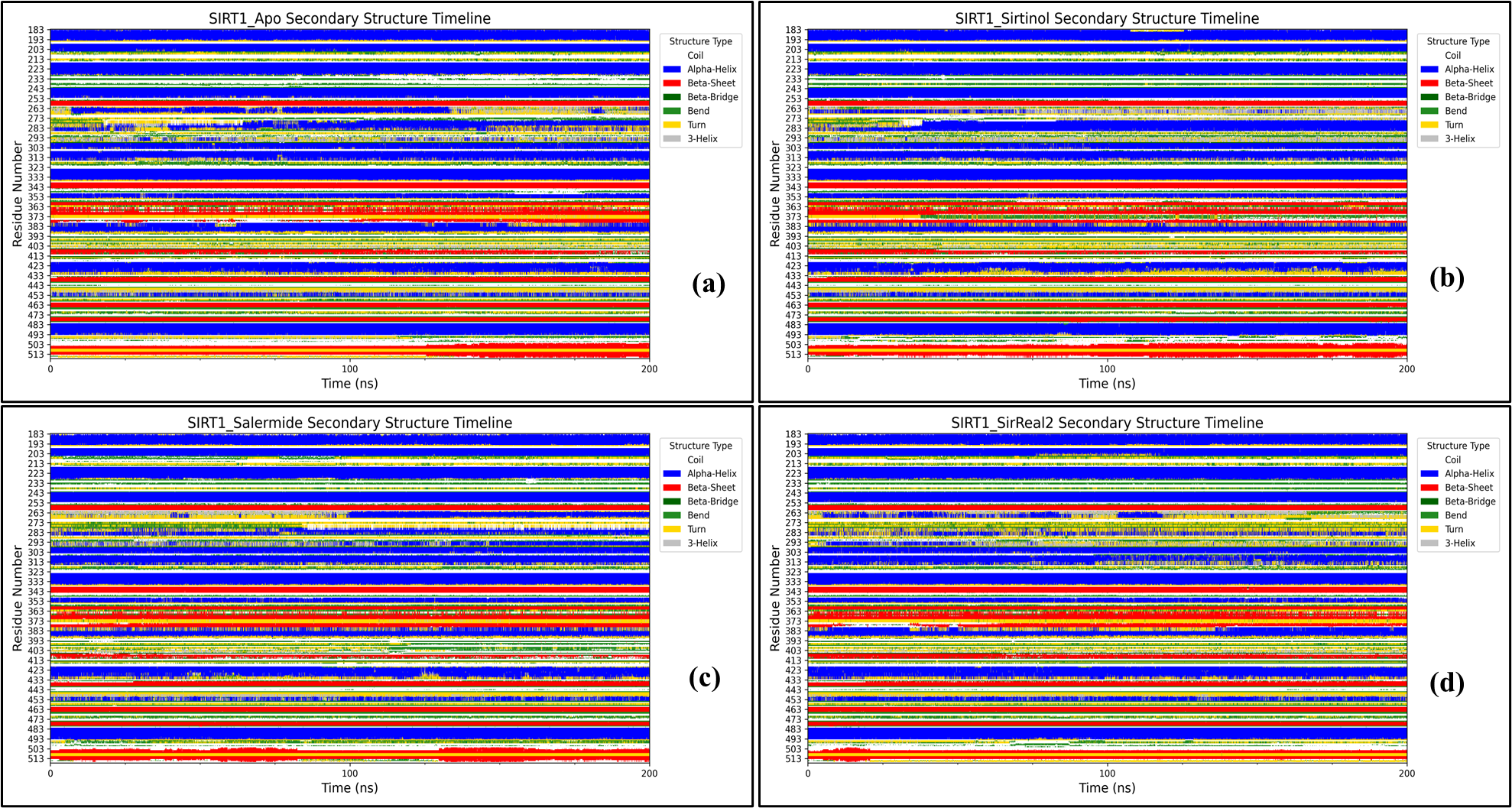


**Fig.** **S5** Pictorial representation of changes in secondary structures (alpha-helix, beta-sheet, beta-bridge, bend, turns, and 3-helix) during the simulation period for SIRT1.


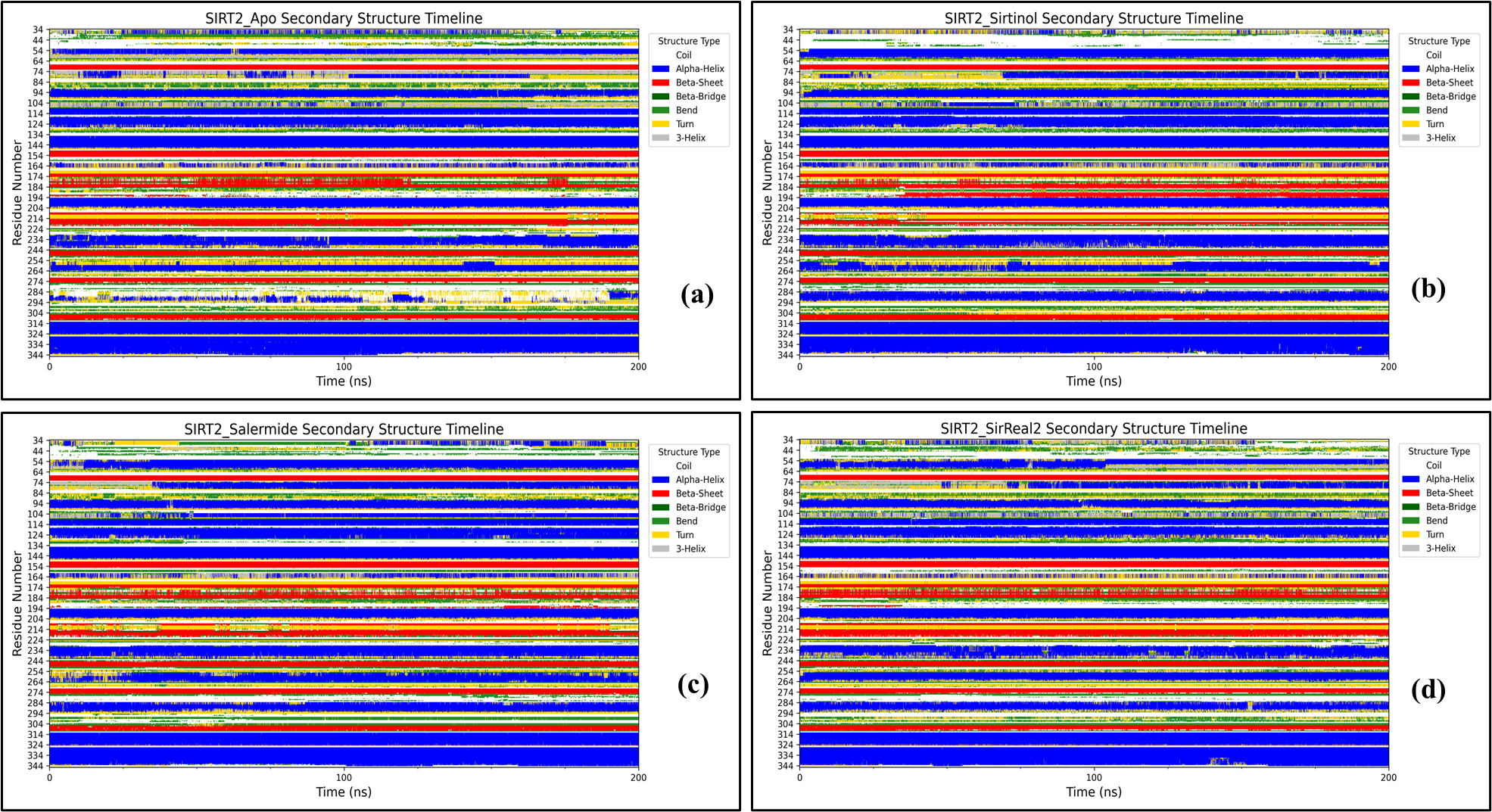


**Fig.** **S6** Pictorial representation of changes in secondary structures (alpha-helix, beta-sheet, beta-bridge, bend, turns, and 3-helix) during the simulation period for SIRT2.


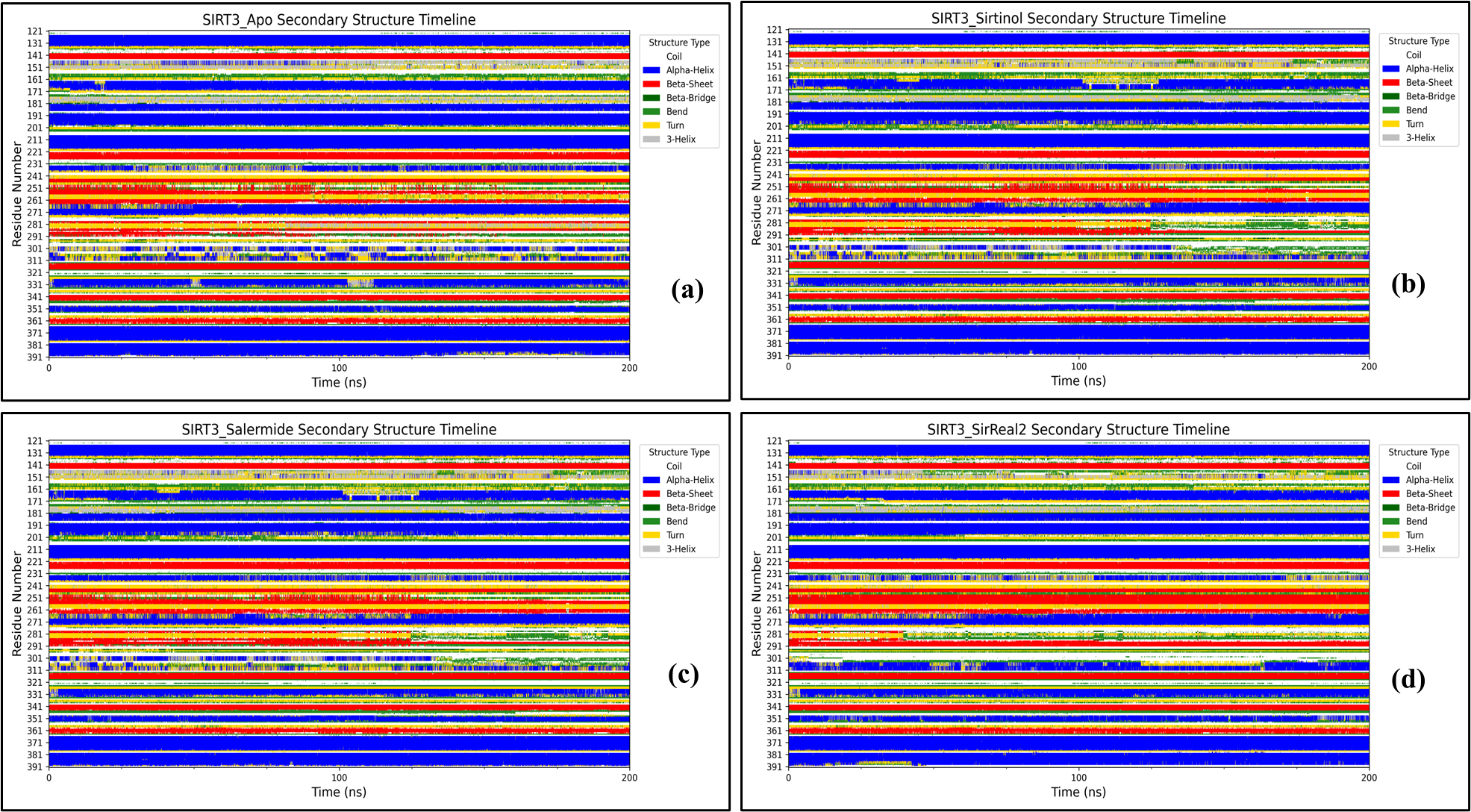


**Fig.** **S7** Pictorial representation of changes in secondary structures (alpha-helix, beta-sheet, beta-bridge, bend, turns, and 3-helix) during the simulation period for SIRT3.


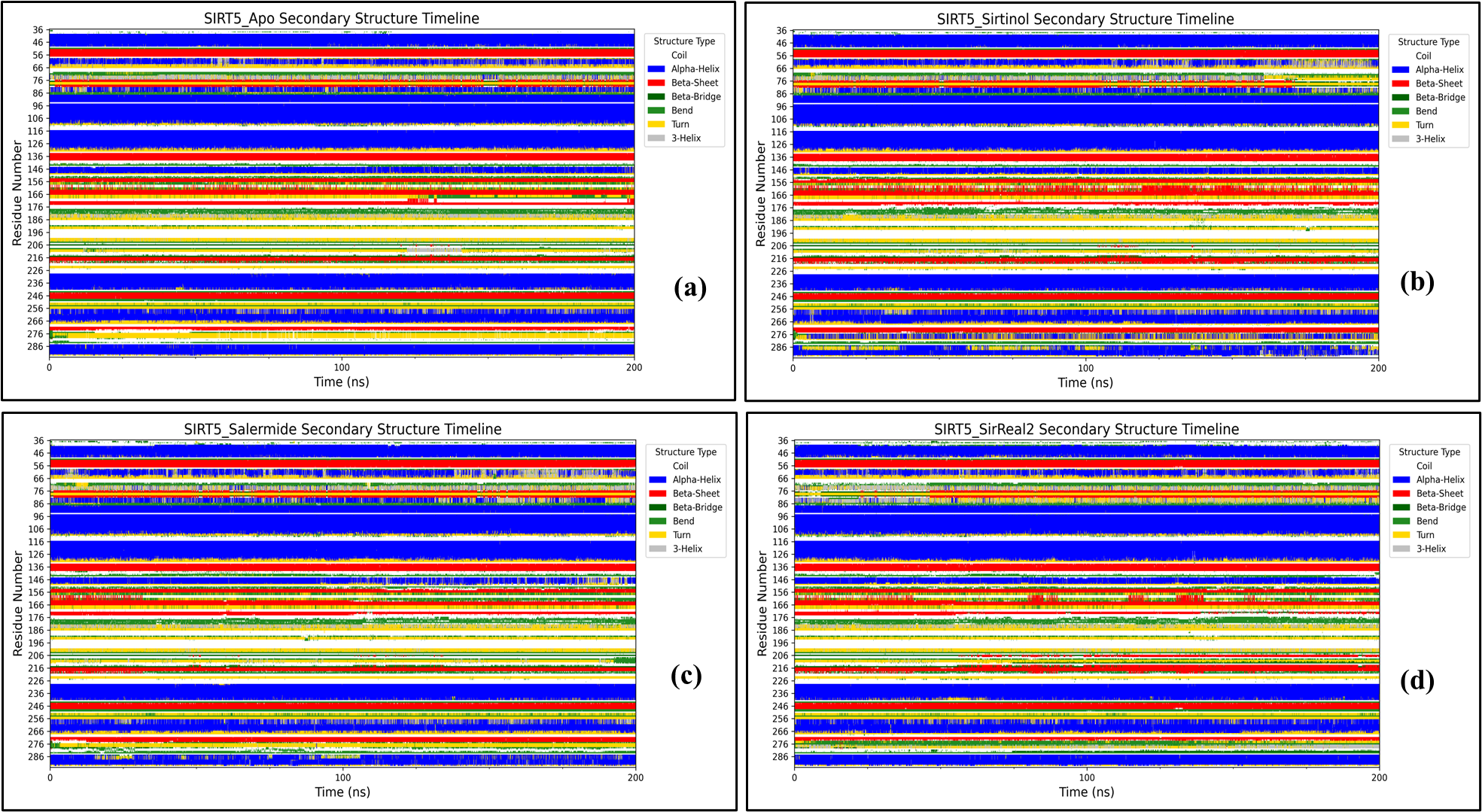


**Fig.** **S8** Pictorial representation of changes in secondary structures (alpha-helix, beta-sheet, beta-bridge, bend, turns, and 3-helix) during the simulation period for SIRT5.


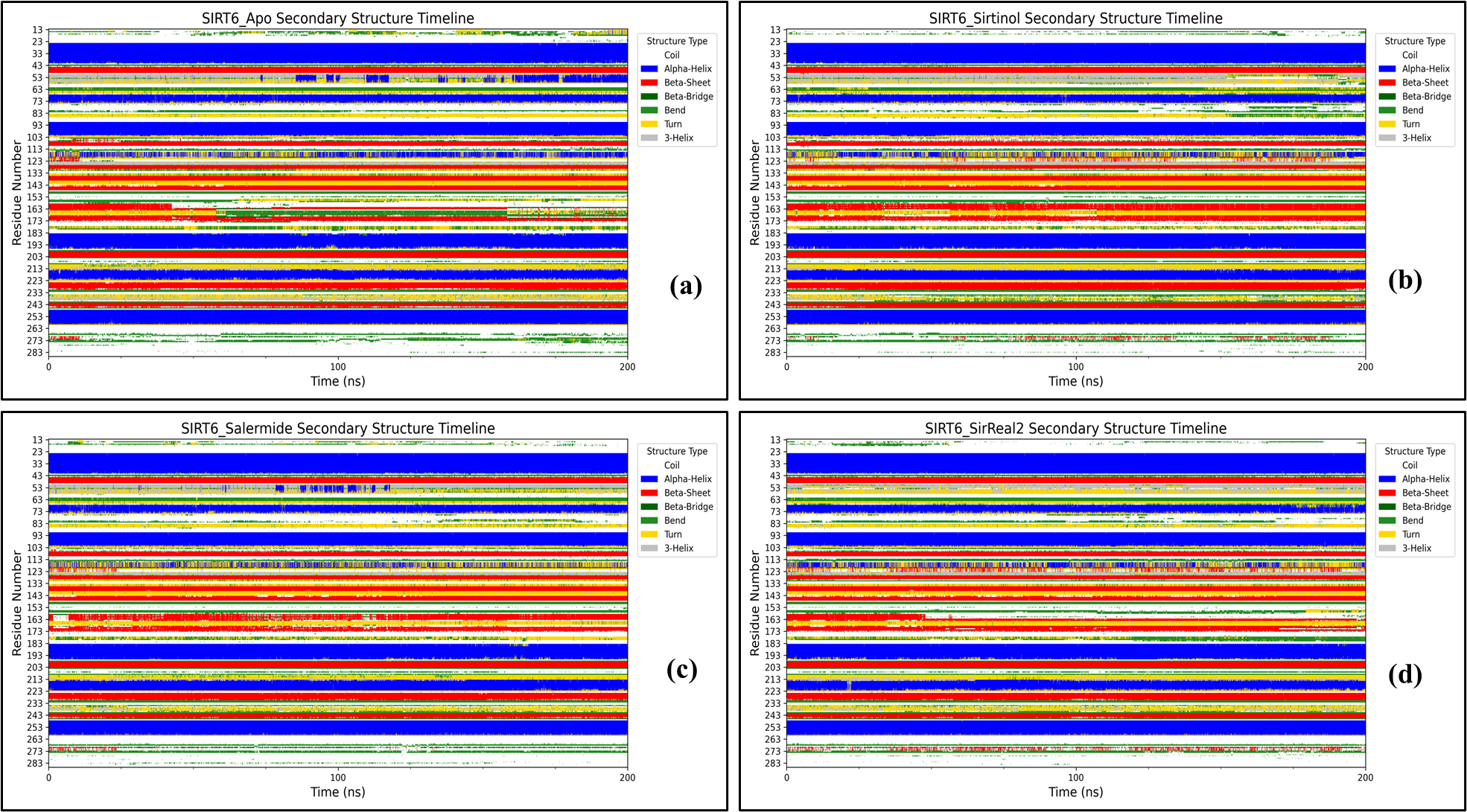


**Fig.** **S9** Pictorial representation of changes in secondary structures (alpha-helix, beta-sheet, beta-bridge, bend, turns, and 3-helix) during the simulation period for SIRT6.


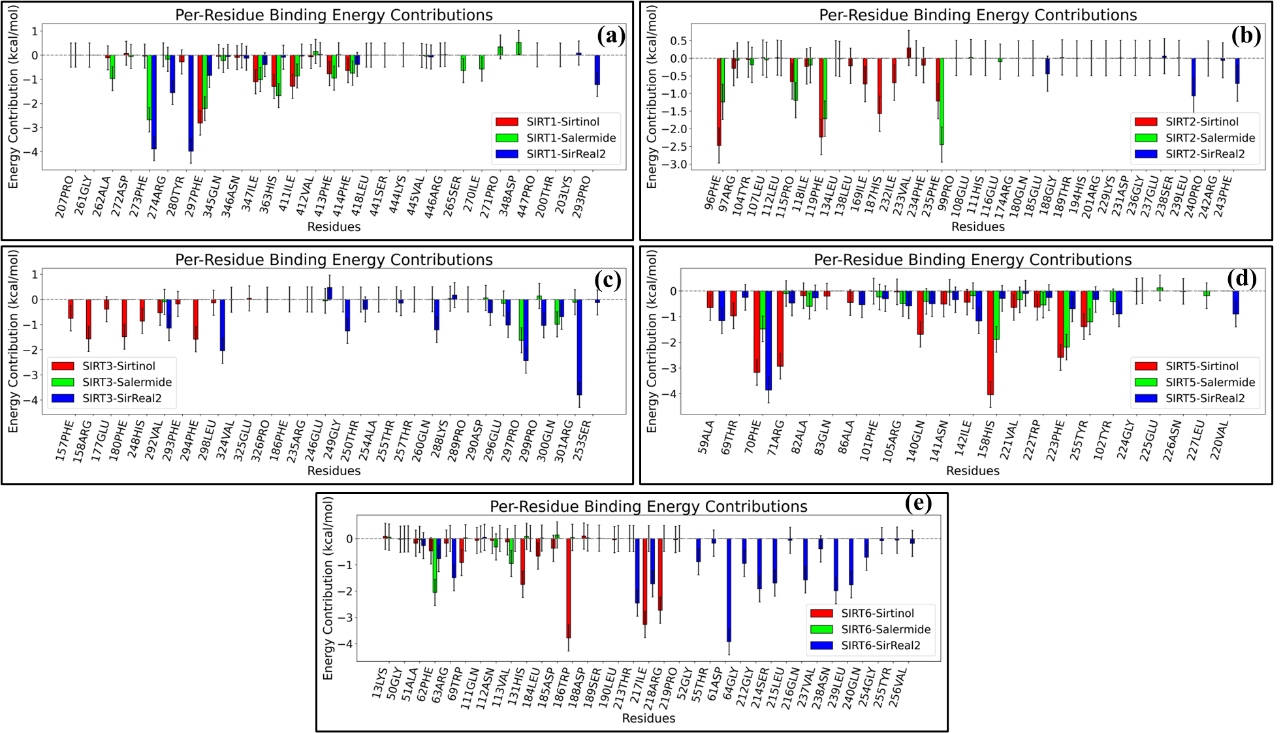


Fig. S10 Representation of per-residue energy contribution during the simulation period for (a) SIRT1, (b) SIRT2, (c) SIRT3, (d) SIRT5, (e) SIRT6 in the top 3 standard ligand-bound states
